# Alternative splicing of CARM1 regulated by *LincGET*-guided paraspeckles biases the first cell fate in mammalian early embryos

**DOI:** 10.1038/s41594-024-01292-9

**Published:** 2024-04-24

**Authors:** Jiaqiang Wang, Yiwei Zhang, Jiaze Gao, Guihai Feng, Chao Liu, Xueke Li, Pengcheng Li, Zhonghua Liu, Falong Lu, Leyun Wang, Wei Li, Qi Zhou, Yusheng Liu

**Affiliations:** 1 College of Life Science, Northeast Agricultural University, Harbin, China; 2 State Key Laboratory of Stem Cell and Reproductive Biology, Institute of Zoology, Chinese Academy of Sciences, Beijing, China; 3 State Key Laboratory of Molecular Developmental Biology, Institute of Genetics and Developmental Biology, Chinese Academy of Sciences, Beijing, China; 4 University of Chinese Academy of Sciences, Beijing, China; 5 Institute for Stem Cell and Regeneration, Chinese Academy of Sciences, Beijing, China; 6 College of Life Science, Northeast Forestry University, Harbin, China

**Keywords:** Reprogramming, Long non-coding RNAs, Alternative splicing

## Abstract

The heterogeneity of CARM1 controls first cell fate bias during early mouse development. However, how this heterogeneity is established is unknown. Here, we show that *Carm1* mRNA is of a variety of specific exon-skipping splicing (ESS) isoforms in mouse two-cell to four-cell embryos that contribute to CARM1 heterogeneity. Disruption of paraspeckles promotes the ESS of *Carm1* precursor mRNAs (pre-mRNAs). *LincGET*, but not *Neat1*, is required for paraspeckle assembly and inhibits the ESS of *Carm1* pre-mRNAs in mouse two-cell to four-cell embryos. We further find that *LincGET* recruits paraspeckles to the *Carm1* gene locus through HNRNPU. Interestingly, PCBP1 binds the *Carm1* pre-mRNAs and promotes its ESS in the absence of *LincGET*. Finally, we find that the ESS seen in mouse two-cell to four-cell embryos decreases CARM1 protein levels and leads to trophectoderm fate bias. Our findings demonstrate that alternative splicing of CARM1 has an important role in first cell fate determination.

## Abstract (web summary)

The heterogeneity of CARM1 for the first cell fate bias in mice arises from exon-skipping splicing of *Carm1* pre-mRNAs, which is regulated by the competition between *LincGET*-paraspeckles and splicing speckles for binding to the *Carm1* locus.

## Main

During mammalian early embryonic development, the first two lineages to emerge are the trophectoderm and the inner cell mass. When and how the first cell fate bias happens remains the topic of intense investigation. The shreds of evidence to date suggest that the initial heterogeneities in CARM1 (which is regulated by the endogenous retrovirus (ERV)-associated nuclear long intergenic non-coding RNA *LincGET*^1,2^) and paraspeckles^3^ is the key epigenetic basis to influence first cell fate determination. However, how the CARM1 heterogeneity is established remains largely unknown. Alternative splicing is the most prominent mechanism to generate mRNA structural complexity^4,5^, which participates in various cell processes^6,7^ including cell fate decisions, as it regulates stem cell differentiation^8–10^ and epithelial–mesenchymal transitions^11–13^.

It is well known that the nuclei contain distinct classes of subnuclear bodies that mediate RNA splicing, including splicing speckles and paraspeckles. The splicing speckles function as storage sites for the splicing factors^14^. Paraspeckles have been reported to be involved in numerous nuclear events, including DNA unwinding, transcriptional regulation, RNA splicing, RNA editing and nuclear retention of some RNAs^15,16^. The function of the subnuclear bodies is determined by their location, which is always regulated by their long non-coding RNA (lncRNA) component^17^. For example, paraspeckles are built around the *Neat1* lncRNA^18,19^. However, *Neat1* is not the only lncRNA involved in paraspeckles. Another lncRNA, termed CTN-RNA, is specifically localized to paraspeckles of numerous cell types^16^. It is reported that alternative splicing during mouse pre-implantation development is extremely complicated and extraordinary, and stage-linked splicing is related to embryonic development^20–23^. Therefore, we wonder whether alternative splicing may contribute to the first cell fate bias.

## Results

### Alternative splicing contributes to CARM1 heterogeneity

**Fig. 1:**
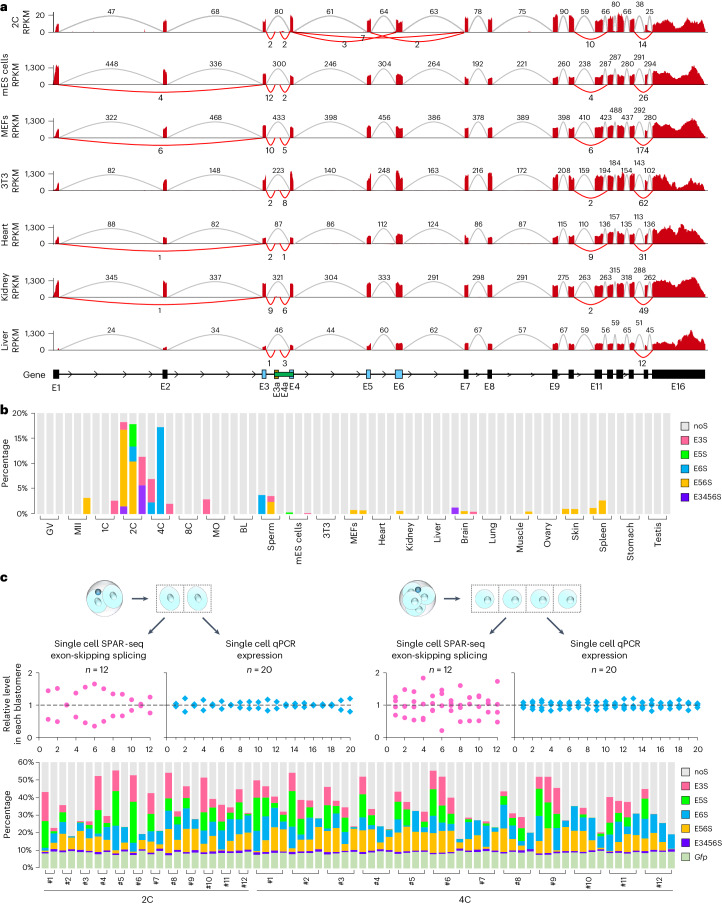
Alternative splicing of exons encoding key SAM-methyltransferase domain of CARM1 is specific and heterogeneous in mouse two-cell to four-cell embryos. **a**, Sashimi plots visualizing that ESS happens on exons 5 and 6 of *Carm1* pre-mRNAs in mouse two-cell (2C)^2^ embryos but not in mouse embryonic stem (mES) cells^51^, mouse embryonic fibroblasts (MEFs)^52^, NIH-3T3 cells, heart^53^, kidney or liver^54^ tissues. Each line represents one set of data. The constitutive splicing events are shown as gray arcs and the alternative splicing events are shown as red arcs. RPKM, reads per kilobase per million mapped reads. **b**, Percentage of ESS events on exons 3 to 6 of *Carm1* pre-mRNAs in mouse gametes, early embryos, cell lines and tissues. Each column represents a sample containing all replicates from the published data of others; three samples were used for each group (see Supplementary Table 1 for details). GV, germinal vesicle; MII, metaphase II; MO, morula stage; noS, non-skipping splicing; BL, blastocyst stage. See Methods for calculations. **c**, Single-cell SPAR-seq results for *Carm1* ESS analysis and single-cell qPCR results for *Carm1* expression level analysis in mouse 2C and 4C embryos. Each dot (top) and each column (bottom) represents one blastomere. The relative level of ESS and expression among blastomeres in the same embryo are shown in red and blue, respectively (top). The percentage of ESS events relative to noS splicing events in a single blastomere of each embryo is shown in the histogram (bottom). Source data

**Extended Data Fig. 1:**
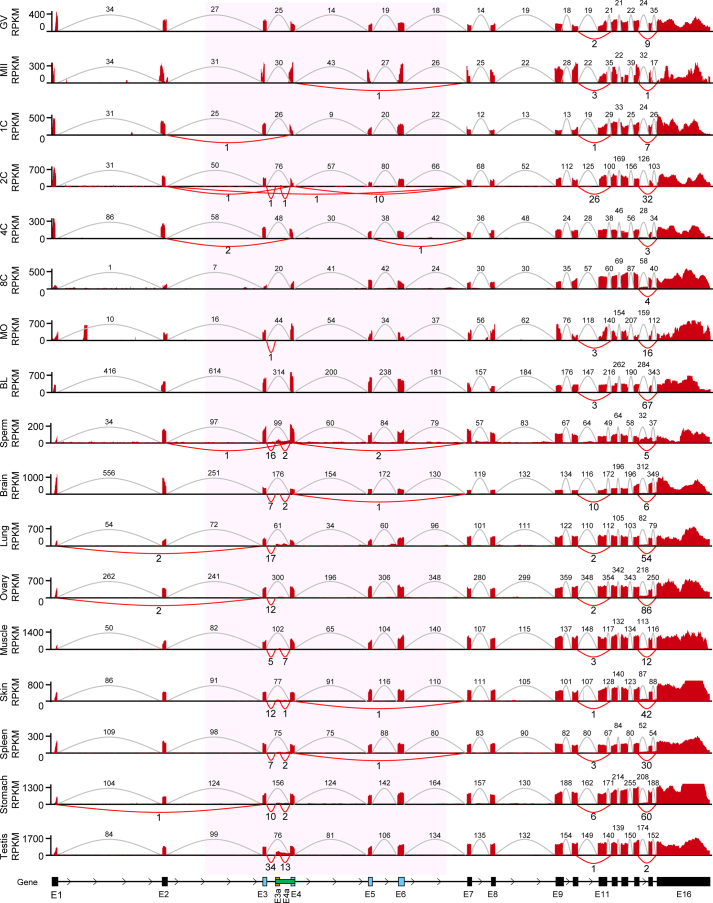
Exon-skipping splicing (ESS) events of *Carm1* pre-mRNAs. Sashimi plots visualizing those ESS events of *Carm1* pre-mRNAs in mouse GV and MII oocytes; one-cell, and two-cell, embryos; sperm; as well as tissues including brain, lung, ovary, muscle, skin, spleen, stomach, and testis. Each line represents one set of data. The constitutive splicing events are shown in grey arcs and the alternative splicing events are shown in the red arcs.

Given that alternative splicing is involved in cell fate decisions^8–13^, we questioned whether alternative splicing contributes to CARM1 heterogeneity, which was established in two-cell to four-cell mouse embryos^3,24^. We analyzed the level of different types of alternative splicing events of *Carm1* pre-mRNAs, including exon-skipping, retained introns, alternative 5′-splicing site, alternative 3′-splicing site, mutually exclusive exons, alternative first exons and alternative last exons, using RNA sequencing (RNA-seq) data from mouse cell lines, tissues, gametes and early embryos. The result indicated a distinguishing feature of the two-cell to four-cell embryos: they presented the highest relative ratio of abnormal ESS events on exons 3 to 6 of *Carm1* pre-mRNAs among all analyzed transcriptomes, including exon 3 skipping (E3S), exon 5 skipping (E5S), exon 6 skipping (E6S), exon 5 and 6 skipping (E56S), and exon 3, 4, 5 and 6 skipping (E3456S). Other alternative splicing types were minimally detectable or not specific in two-cell to four-cell embryos (Fig. 1a,b, Extended Data Fig. 1, Supplementary Fig. 1 and Supplementary Table 1).

**Extended Data Fig. 2:**
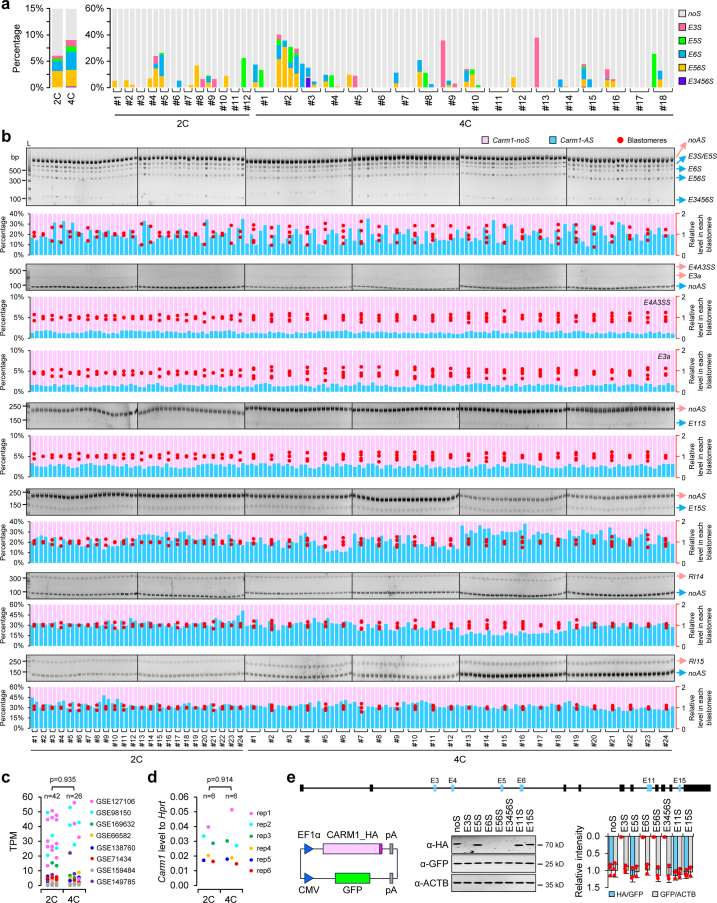
Heterogeneity of exon 3 to 6 skipping splicing of *Carm1* pre-mRNAs. **a**. Percentage of ESS events in mouse two- and four-cell embryos in total (left) or in each blastomere (right) sequenced via PacBio platform. **b**. The combination charts of bar plots depicting percentage of alternative splicing of *Carm1* pre-mRNAs (E3S, E5S, E6S, E56S, E3456S, E4A3SS, E11S, E15S, RI14, and RI15) in mouse late two- and early four-cell embryos and dot plots depicting the relative level of alternative splicing of *Carm1* pre-mRNAs among blastomeres in each embryo. Each dot or column respects one blastomere. noAS, non-alternative splicing. L, DNA ladder. Note that the results for exon 3 to 6 skipping splicing are also used in Extended Data Fig. 9b (L2C) and Extended Data Fig. 9c (E4C), and the spatial position information are shown in Extended Data Fig. 9b, c but not here. **c**, **d**. The dot plot depicting *Carm1* RNA levels based on RNA-seq data from two-cell embryos (n = 42) and four-cell embryos (n = 26) (c), and the qPCR results (d). **e**. The western blot results showing that the exon-skipping events of exons 3 to 6, but not exon 11 or 15, of *Carm1* pre-mRNAs seem to affect the protein level of CARM1. Top panel, illustration of exons of *Carm1* pre-mRNA. Bottom left panel, illustration of the vector from which HA-tagged CARM1 and green fluorescent protein (GFP) are expressed simultaneously, in which GFP works as a reference for exogenous transfection. Bottom middle panel, the western blot results. Bottom right panel, the combination charts of bar plots and dot plots showing the relative intensities of HA to GFP (HA/GFP) and GFP to ACTB (GFP/ACTB). EF1α, the promoter of human translation elongation factor 1 alpha. CMV, the promoter of cytomegalovirus. pA, the polyadenylation signal element. kD, kilodalton. Source data

Intrigued by these distinguishing features, we used systematic parallel analysis of endogenous RNA regulation coupled to barcode sequencing (SPAR-seq)^25^ to deeply analyze the ESS events around exons 3 to 6 of *Carm1* pre-mRNAs in mouse two-cell and four-cell embryos. Firstly, the SPAR-seq library was sequenced on a PacBio third-generation sequencer, and we confirmed the existence of ESS events around exons 3 to 6 of *Carm1* pre-mRNAs (Extended Data Fig. 2a and Supplementary Tables 2 and 3). Then we performed SPAR-seq at a single-cell level on an Illumina MiSeq sequencer under PE300 mode, using the synthetic *Gfp* RNA fragment as the spike-in. Consequently, we observed heterogeneous ESS of *Carm1* pre-mRNAs between blastomeres in both two-cell and four-cell embryos, whereas the levels of total *Carm1* transcripts were consistent among blastomeres as measured by single-cell quantitative PCR (qPCR) analysis (Fig. 1c). These results were confirmed by gel electrophoresis analysis of SPAR assays (Extended Data Fig. 2b). In addition, the RNA-seq results showed that the transcript levels of *Carm1* are similar between the two-cell and the four-cell stages (Extended Data Fig. 2c), which was confirmed by qPCR (Extended Data Fig. 2d), reflecting a relatively stable transcription of *Carm1* in two-cell to four-cell embryos.

The above results indicate that ESS on exons 3 to 6 of *Carm1* pre-mRNAs, encoding a key SAM-methyltransferase domain of CARM1, is specific and heterogeneous in mouse two-cell to four-cell embryos, which raises a new level of CARM1 that is heterogeneous for first cell fate bias. Therefore, we focus on ESS events on exons 3 to 6 of *Carm1* pre-mRNAs.

Although the ratios of exon 15 skipping splicing (E15S) and exon 11 skipping splicing (E11S) are probably higher than the skipping of exons 3 to 6, we did not focus on E15S and E11S for two reasons. First, E15S and E11S do not affect the CARM1 protein level, which was confirmed by transforming them into HEK293T cells and detecting the translated products with western blotting using anti-HA antibody (Extended Data Fig. 2e). Second, E15S and E11S are not specific for two-cell and four-cell embryos, in which CARM1 heterogeneity is established.

### Paraspeckle components inhibit the ESS of *Carm1* pre-mRNAs

**Fig. 2:**
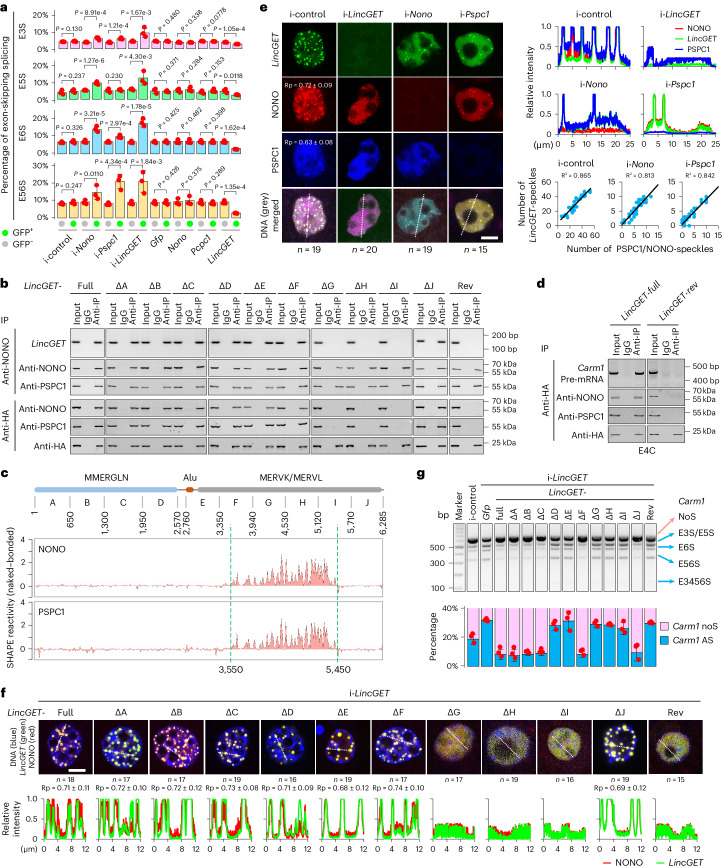
*LincGET*-guided assembly of paraspeckles is essential for protecting *Carm1* from ESS. **a**, Combination charts of bar plots and dot plots showing the percentage of ESS events of *Carm1* pre-mRNAs in 4C embryos measured by SPAR-seq. Data are mean and s.e.m. One-tailed Student’s *t*-tests were used for statistical analysis (*n* = 3 biological replicates). i- denotes knockdown by RNA interference. **b**, Co-IP followed by RT–PCR and western blot in mouse epiblast stem cells (mEpiSCs). Three biological replicates were performed. Full denotes full-length *LincGET* without LNA-targeting sites; rev denotes the reverse sequence of *LincGET*. **c**, SHAPE-MaP assays. Alu denotes transposon elements originally from *Arthrobacter luteus* restriction endonuclease; MMERGLN, MERVK and MERVL are transposon elements from mouse ERV with tRNA^Gln^, tRNA^Lys(K)^ and tRNA^Leu(L)^, respectively. **d**, Co-IP followed by RT–PCR and western blot in early 4C (E4C) embryos. Three biological replicates were performed. **e**, Immunofluorescence combined with RNA-FISH assays in late 2C (L2C). Scale bar, 10 μm. Top right, line scans of the relative fluorescence intensity of signals indicated by the dotted line in the left panel. Bottom right, the relationship between the numbers of *LincGET* speckles and NONO or PSPC1 speckles calculated by Imaris (19 × 4 cells of i-control group, 19 × 4 cells of i-*Nono* group, and 15 × 4 cells of i-*Pspc1* group) is shown in dot plots. **f**, Single nucleus of L2C injected with fluorescently labeled *LincGET* and NONO. Scale bar, 10 μm. Bottom, line scans of the relative fluorescence intensity of signals indicated by the dotted line in the top panel. **g**, Agarose gel analysis of RT–PCR (top) and quantification (bottom). AS, alternative splicing. Data are mean and s.e.m. One-tailed Student’s *t*-tests were used for statistical analysis and *P* values are shown in Extended Data Fig. 4d (*n* = 3 biological replicates). Rp values in **e** and **f** were calculated by Fiji/ImageJ. Source data

**Extended Data Fig. 3:**
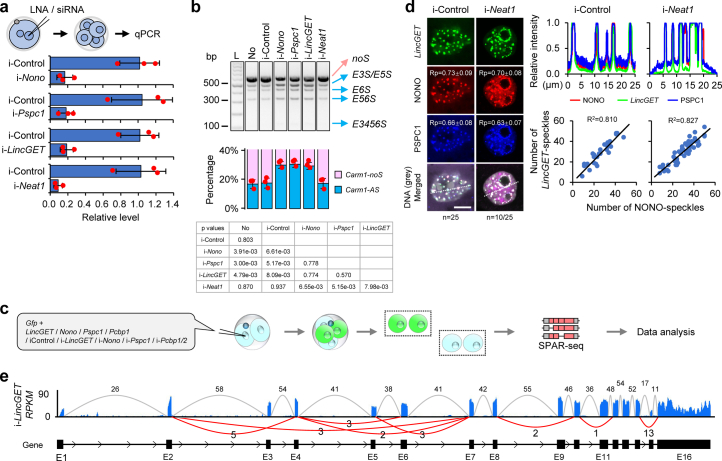
*LincGET* participates in paraspeckles in mouse two- to four-cell embryos. **a**. Interference efficiency analysis of siRNAs on *Nono* and *Pspc1*, as well as locked nucleic acid (LNA) on *LincGET* and *Neat1*. Data are presented as mean values ± SEM (n = 3 biological replicates). **b**. Agarose gel analysis of RT-PCR showing changes of ESS of *Carm1* pre-mRNAs upon knockdown of *Nono*, *Pspc1*, *Neat1*, or *LincGET*. The percentages are shown below in combination charts of bar plots and dot plots. Data are presented as mean values ± SEM. One-tailed Student’s t-tests were used for statistical analysis and p values are shown in the table below (n = 3 biological replicates). L, DNA ladder. No, group injected with nothing. **c**. Illustration of Systematic Parallel Analysis of Endogenous RNA Regulation Coupled to Barcode Sequencing (SPAR-seq). **d**. Fluorescence images showing *Neat1* depletion results in partial re-localization of NONO and PSPC1 from paraspeckles to the periphery of the nucleoli. Scale bar, 10 μm. The Rp values are calculated by Fiji/ImageJ and shown on the pictures. Top right panel, line scans of the relative fluorescence intensity of signals indicated by the dotted line in left panel. Bottom right panel, the relationship between the numbers of *LincGET*-speckles and NONO-speckles calculated by Imaris (19 × 4 cells of i-Control group and 25 × 4 cells of i-*Neat1* group) is shown in the dot plot, and the R2 values were calculated in Excel. **e**. Sashimi plots visualizing that ESS happens on exons 3 to 6 of *Carm1* pre-mRNAs in mouse two-cell embryos upon *LincGET* depletion. The constitutive splicing events are shown in grey arcs and the alternative splicing events are shown in the red arcs. Note that the control group for *LincGET* depletion is the sample 2C in Fig. 1a, in which the control LNA is injected. Source data

Given that paraspeckles have been reported to regulate RNA splicing^15,16^ and pre-implantation mouse embryo development^3^, we wondered whether paraspeckle components regulate ESS of *Carm1* pre-mRNAs in mouse early embryos. Overexpression of *Nono* or *Pspc1* did not affect the ESS of *Carm1* pre-mRNAs; however, depletion of *Nono* or *Pspc1* increased the ESS of *Carm1* pre-mRNAs in mouse four-cell embryos, which was evident not only from the reverse transcription PCR (RT–PCR) results (Extended Data Fig. 3a,b) but also from SPAR-seq (Fig. 2a and Extended Data Fig. 3c). These results suggest that paraspeckle components inhibit the ESS of *Carm1* pre-mRNAs in mouse early embryos.

### *LincGET* guides paraspeckle assembly in mouse early embryos

Paraspeckles in somatic cells are built around *Neat1* (ref. ^18^); however, we found that *Neat1* ablation did not affect the ESS of *Carm1* pre-mRNAs (Extended Data Fig. 3a,b). Moreover, we found that paraspeckles were assembled normally in mouse four-cell embryos upon *Neat1* ablation, along with partial re-localization of NONO from paraspeckles to the periphery of the nucleoli in 10 out of 25 embryos (Extended Data Fig. 3d), which is consistent with previous results^3^. These results reflect that *Neat1* is not essential for paraspeckle assembly in mouse early embryos. Therefore, we questioned whether there are other lncRNAs essential for the paraspeckle organization in mouse early embryos.

We previously found that *LincGET* forms granules with CARM1 in the nucleus of mouse two-cell to four-cell embryos^1^, and it has been reported that CARM1 is involved in paraspeckles during this stage^3^. Interestingly, *LincGET* depletion increases and *LincGET* overexpression decreases the ESS of *Carm1* pre-mRNAs (Fig. 2a and Extended Data Fig. 3a–c), which is consistent with the previous RNA-seq data^2^ (Extended Data Fig. 3e) and raises a possibility that *LincGET* may participate in the organization of paraspeckles in mouse early embryos.

**Extended Data Fig. 4:**
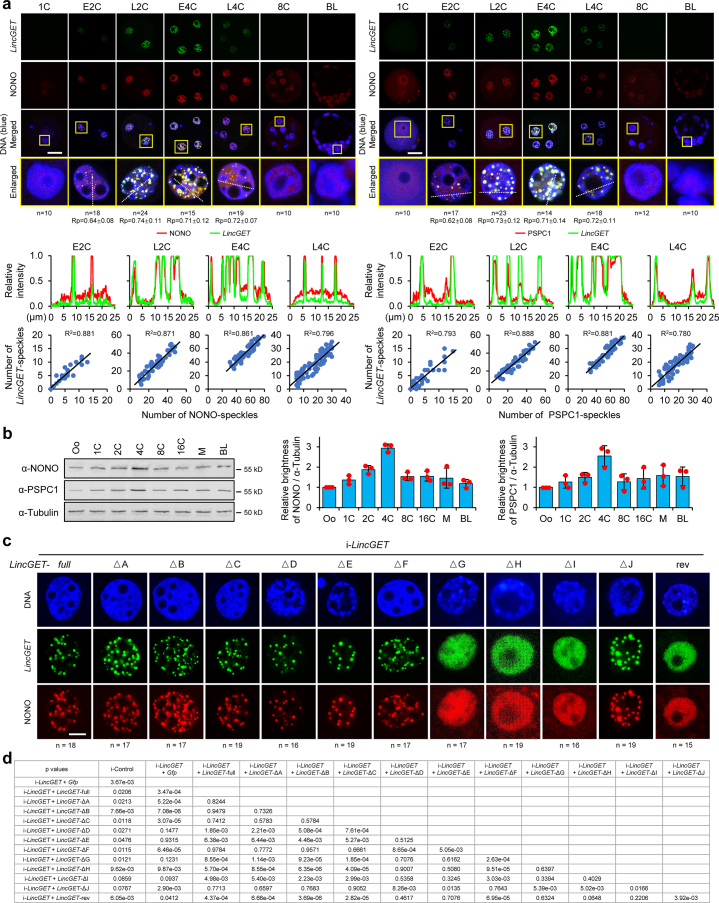
*LincGET* localizes to paraspeckles. **a**. Fluorescence images reflecting the expression pattern of *LincGET* and NONO (left) or PSPC1 (right) at different stages of mouse preimplantation embryos. Scale bar, 50 μm. Representative nuclei are shown below. The Rp values for co-localization of *LincGET* and NONO (left) or PSPC1 (right) in early two-cell embryos (E2C), late two-cell embryos (L2C), early four-cell embryos (E4C), and late four-cell embryos (L4C) are calculated by Fiji/ImageJ. Middle panel, line scans of the relative fluorescence intensity of signals indicated by the dotted lines in top panel. Bottom panel, the relationship between the numbers of *LincGET*-speckles and NONO-speckles (left, 18 × 2 cells of E2C, 24 × 2 cells of L2C, 15 × 4 cells of E4C, and 19 × 4 cells of L4C) or PSPC1-speckles (right, 17 × 2 cells of E2C, 23 × 2 cells of L2C, 14 × 4 cells of E4C, and 18 × 4 cells of L4C) calculated by Imaris is shown in the dot plot, and the R^2^ (coefficient of determination) values were calculated in Excel. 1C, one-cell embryos; 8C, eight-cell embryos; BL, blastocyst. **b**. Western blot results showing the expression pattern of NONO and PSPC1 during mouse early embryonic development. Three biological replicates were performed. Tubulin is used as a control. α-, anti. Data are presented as mean values ± SEM (n = 3 biological replicates). kD, kilodalton. **c**. Single channel of fluorescence images in Fig. 2f showing that *LincGET* deletion mutants in NONO/PSPC1 binding domain (ΔG, ΔH, or ΔI) cannot form speckles in E4C embryos. The *LincGET* signals were detected from the large amounts of injected *LincGET* or mutants. Scale bar, 10 μm. **d**. The p values for Fig. 2g. One-tailed Student’s t-tests were used for statistical analysis (n = 3 biological replicates). Source data

The expression patterns of NONO and PSPC1 were highly consistent with that of *LincGET*, which bursts at the two-cell to four-cell stage (Extended Data Fig. 4a,b). Further analysis indicated the co-localization between *LincGET* and paraspeckles in mouse two-cell and four-cell embryos (Pearson’s correlation coefficient (Rp) ≈ 0.7; Extended Data Fig. 4a).

To further confirm that *LincGET* is localized to paraspeckles, we tested the interaction between *LincGET* and components of paraspeckles using MS2-fused *LincGET* (*LincGET-MS2*) and an HA-tagged MS2 bacteriophage coat proteins (MS2P) system^26^. We performed RNA immunoprecipitation (RIP) and co-immunoprecipitation (co-IP) assays and found that *LincGET* forms complexes with NONO and PSPC1 (Fig. 2b). Furthermore, with a series of *LincGET*-*MS2* mutants, we found that the G-H-I fragment of *LincGET* is essential for its binding to NONO and PSPC1 (Fig. 2b).

To confirm these results, we performed selective 2′-hydroxyl acylation analyzed by primer extension and mutational profiling (SHAPE-MaP) assays^27^. By comparing the SHAPE-MaP data from in vitro transcribed *LincGET* under protein-free conditions (naked) and upon incubation with NONO or PSPC1 (bonded), we found that NONO and PSPC1 bind the 3,550–5,450 fragments of nucleotides of *LincGET* (Fig. 2c). The NONO/PSPC1-binding region of *LincGET* is enriched of sequences from MERVL and MERVK (Fig. 2c), which is consistent with the results of others^28^. We injected the *LincGET-MS2*, HA-tagged MS2P and NONO at the pronuclear stage and performed co-IP at the early four-cell stage. The results showed that *LincGET* forms complexes with NONO, PSPC1 and *Carm1* pre-mRNA in mouse four-cell embryos (Fig. 2d).

We found that most *LincGET* speckles disintegrated into the nucleoplasm without either NONO or PSPC1, and the paraspeckles disappeared when *LincGET* was depleted (Fig. 2e), just like *Neat1* ablation for paraspeckles in HeLa and NIH-3T3 cells^18^. Together, these results indicate that *LincGET*, rather than *Neat1*, guides the assembly of paraspeckles in mouse two-cell to four-cell embryos, which differs from paraspeckles in somatic cells.

### Paraspeckle assembly is essential for *Carm1* ESS inhibition

As *LincGET* regulates the ESS of *Carm1* pre-mRNAs and guides the assembly of paraspeckles in mouse two-cell to four-cell embryos, we wondered whether the assembly of paraspeckles is essential for inhibiting ESS of *Carm1* pre-mRNAs. Therefore, we injected *Nono* and a series of truncated *LincGET* into mouse two-cell embryos in which the endogenous *LincGET* was depleted and found that no paraspeckles formed in ΔG, ΔH and ΔI groups (Fig. 2f and Extended Data Fig. 4c), indicating that the interaction between *LincGET* and NONO is needed for paraspeckle assembly in mouse early embryos. Moreover, we found that the ESS of *Carm1* pre-mRNAs increased when the endogenic *LincGET* was replaced by deletion mutants without the NONO/PSPC1-binding domain (ΔG, ΔH and ΔI in Fig. 2g and Extended Data Fig. 4d), reflecting that assembled paraspeckles are essential for inhibiting ESS of *Carm1* pre-mRNAs.

### The D–E domain of *LincGET* regulates paraspeckle localization

Notably, the ESS of *Carm1* pre-mRNAs also increased when the endogenic *LincGET* was replaced by deletion mutants without the D–E domain (Fig. 2g, Extended Data Fig. 4d), whereas the assembly of paraspeckles is normal in deletion mutants without the D–E domain (Fig. 2f and Extended Data Fig. 4c), indicating that other mechanisms in addition to paraspeckle assembly are involved in the inhibition of ESS of *Carm1* pre-mRNAs.

**Fig. 3:**
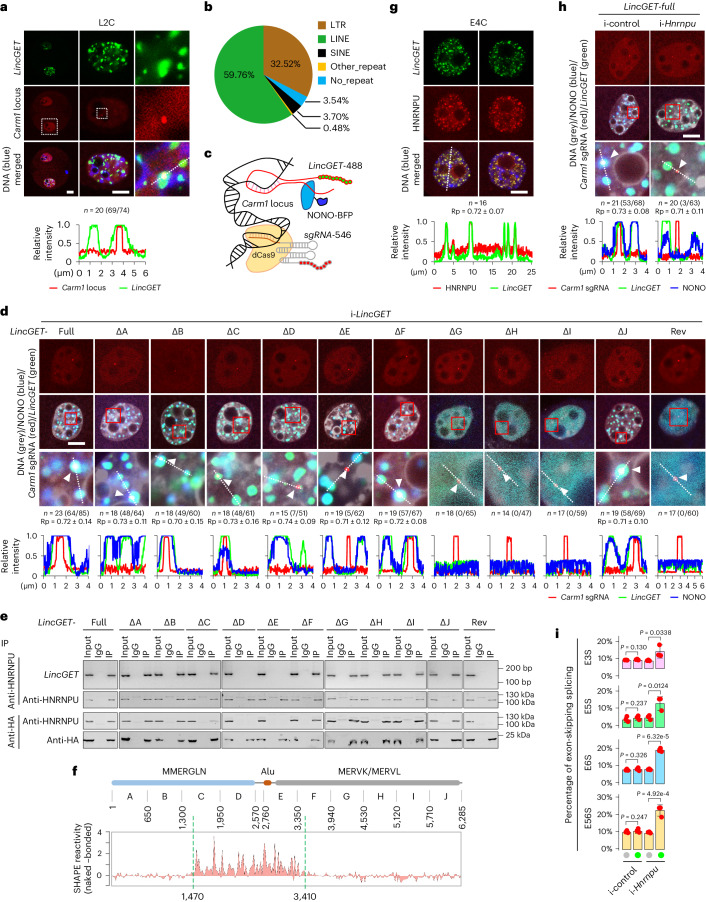
Interaction between *LincGET* and HNRNPU guides localization of paraspeckle to *Carm1* gene locus to inhibit ESS of *Carm1.* **a**, RNA-FISH combined with DNA-FISH assays in L2C. Scale bar, 10 μm. *n* = 20(69/74), which indicates 20 embryos were analyzed, 74 *Carm1* gene loci were detected and 69 of them were covered by *LincGET*. Bottom, relative fluorescence intensity. **b**, Pie chart showing the binding preference of *LincGET*. **c**, Model for co-localization of fluorescently labeled *LincGET*, sgRNA–dCas9 complex and NONO. **d**, Single nucleus of L2C injected with fluorescently labeled *LincGET* mutants, sgRNA–dCas9 complex and NONO. White triangles indicate sgRNA signals. Scale bar, 10 μm. The *n* values are as in **a**. Bottom, relative fluorescence intensity. **e**, Co-IP followed by RT–PCR and western blot in mEpiSCs. Three biological replicates were performed. **f**, SHAPE-MaP assays. **g**, Immunofluorescence combined with RNA-FISH assays in E4C. Scale bar, 10 μm. Bottom, relative fluorescence intensity. **h**, Immunofluorescence combined with RNA-FISH assays in L2C. Scale bar, 10 μm. White triangles indicate sgRNA signals. The *n* values are as in **a**. Bottom, relative fluorescence intensity. **i**, Combination charts showing changes of ESS of *Carm1* pre-mRNAs. Data are mean and s.e.m. One-tailed Student’s *t*-tests were used for statistical analysis (*n* = 3 biological replicates). Rp values in **d,** **g** and **h** were calculated by Fiji/ImageJ. Source data

**Extended Data Fig. 5:**
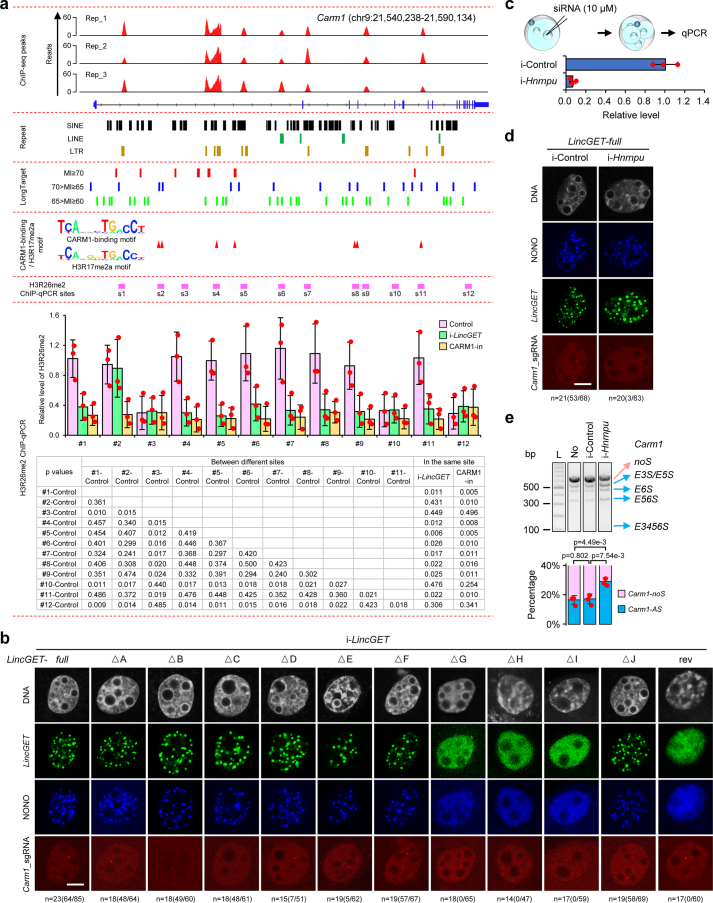
*LincGET* binds repeat sequences. **a**. Chromatin immunoprecipitation (ChIP) results showing that *LincGET* binds repeat sequences including SINE, LINE, and LTR in *Carm1* gene loci in E4C embryos. n = 3 biological replicates (rep_1, rep_2, and rep_3). ChIP-seq peaks lane, visualization of ChIP-seq peaks on *Carm1* gene locus. Repeat lane, the SINE, LINE, and LTR repeat elements in *Carm1* gene loci. SINE, short interspersed nuclear elements. LongTarget lane, the sequence complementarity of *LincGET* to the *Carm1* locus analyzed by *LongTarget*. MI, MeanIdentidy. CARM1-binding motif and H3R17me2a motif lane, the predicted CARM1-binding/H3R17me2a motif generated from the ChIP-seq data (PMID: 30456381). H3R17me2a, asymmetrically histone H3 arginine 17 dimethylation. H3R26me2 ChIP-qPCR sites lane, the tested sites by H3R26me2 ChIP-qPCR. H3R26me2, histone H3 arginine 26 dimethylation. H3R26me2 ChIP-qPCR lane top panel, combination charts of bar plots and dot plots showing the results of H3R26me2 ChIP-qPCR. Data are presented as mean values ± SEM (n = 3 biological replicates). H3R26me2 ChIP-qPCR lane bottom panel, one-tailed Student’s t-tests were used for statistical analysis in H3R26me2 ChIP-qPCR lane top panel and the p values are shown in the table. For p values in the table, the left panel ‘Between different sites’ are p values between the control groups of different sites; the right panel ‘In the same sites’ are p values between the *LincGET* depletion (i-*LincGET*) and CARM1 inhibition (CARM1-in) groups of each site. **b**. Single channel of fluorescence images in Fig. 3d. Scale bar, 10 μm. The n values are shown as in Fig. 3d. **c**. Interference efficiency analysis of siRNAs on *Hnrnpu*. Data are presented as mean values ± SEM (n = 3 biological replicates). **d**. Single channel of fluorescence images in Fig. 3h. Scale bar, 10 μm. The n values are shown as in Fig. 3h. **e**. Agarose gel analysis of RT-PCR showing changes of ESS of *Carm1* pre-mRNAs upon *Hnrnpu* depletion. The percentages are shown below in combination charts of bar plots and dot plots. Data are presented as mean values ± SEM. One-tailed Student’s t-tests were used for statistical analysis (n = 3 biological replicates). L, DNA ladder. No, group injected with nothing. Source data

As pre-mRNA is subjected to splicing during its synthesis^29^, we questioned whether the localization of paraspeckles is the mechanism beyond paraspeckle assembly. Using RNA fluorescence in situ hybridization (RNA-FISH) combined with DNA-FISH, we found that *LincGET* locates to the *Carm1* gene locus (Fig. 3a), reflecting that a paraspeckle assembles around the *Carm1* gene locus. To confirm these results, we injected the *LincGET-MS2* and HA-tagged MS2P at the pronuclear stage and performed chromatin immunoprecipitation followed by high-throughput sequencing (ChIP–seq) using an anti-HA antibody at the early four-cell stage (*LincGET* ChIP–seq) to explore the genome-wide *LincGET-*binding sites. The results showed that *LincGET* indeed binds around the *Carm1* gene locus, tending to bind long terminal repeats (LTRs) (Extended Data Fig. 5a). Generally, we found that *LincGET* tends to bind repeat elements, such as long interspersed nuclear elements (LINEs, 59.76%) and LTRs (32.52%) (Fig. 3b), which is consistent with our reports that *LincGET* increases chromatin openness around retrotransposon elements^1^.

It has been reported that lncRNA *Terra* consists of repeat sequences and maintains telomeric structure through sequence complementarity with R-loop formation^30^. As *LincGET* also consists of repeat sequences, most of which are from ERV, we reasoned that *LincGET* can bind ERV-associated LTRs by sequence complementarity. To test this hypothesis, we used the LongTarget prediction tool^31^ and found that *LincGET* binds to the *Carm1* gene locus at multiple sites, especially the LTRs and LINEs with higher mean identity (Extended Data Fig. 5a and Supplementary Table 4), which is consistent with the results of *LincGET* ChIP–seq. These results suggested that *LincGET* binds to the *Carm1* gene locus through sequence complementarity.

Next, we questioned whether the D–E domain of *LincGET* guides the localization of paraspeckles. We took advantage of CRISPR-mediated live imaging of the genome^32^ (Supplementary Table 3), and used blue fluorescent protein (BFP)-labeled NONO to visualize paraspeckles (Fig. 3c). As a result, we discovered that no paraspeckles formed when the endogenic *LincGET* was replaced by deletion mutants without the G, H or I domain (Fig. 3d and Extended Data Fig. 5b) and paraspeckles formed but no longer targeted the *Carm1* gene locus when the endogenic *LincGET* was replaced by deletion mutants without the D or E domain (Fig. 3d and Extended Data Fig. 5b). These results reveal that the NONO/PSPC1-binding domain of *LincGET* is essential for paraspeckle assembly, and the D–E domain of *LincGET* is essential for paraspeckle localization to the *Carm1* gene locus, both of which are needed for inhibiting ESS of *Carm1* pre-mRNAs.

### *LincGET*–HNRNPU complex guides paraspeckle localization

Given that the interaction between RNA and chromatin needs chromatin-associated proteins^33^, we reasoned that chromatin-associated proteins bind the D–E domain of *LincGET* and regulate its association with the *Carm1* gene locus. We previously reported that *LincGET* binds heterogeneous nuclear ribonucleoprotein U (HNRNPU)^2^, which has a critical role in the high-order organization of the nucleus^34^. We next wondered whether the D–E domain of *LincGET* interacts with HNRNPU. Results of the RIP and co-IP assays showed that the D–E domain of *LincGET* is crucial for HNRNPU binding (Fig. 3e), and these findings were confirmed by the SHAPE-MaP assays (Fig. 3f), indicating that the D–E domain of *LincGET* interacts with HNRNPU. In addition, the analysis of fluorescence staining results indicated the co-localization between *LincGET* and HNRNPU in mouse four-cell embryos (Rp ≈ 0.70; Fig. 3g).

We next investigated whether HNRNPU is essential for mediating the chromatin localization of *LincGET*-guided paraspeckles. We observed that HNRNPU depletion (Extended Data Fig. 5c) did not affect the assembly of paraspeckles but did result in an inability of paraspeckles to localize to the *Carm1* gene locus (Fig. 3h and Extended Data Fig. 5d), reflecting that HNRNPU is necessary for the localization of paraspeckles to the *Carm1* gene locus. Therefore, we conclude that the *LincGET*–HNRNPU interaction is indispensable for the correct localization of paraspeckles in mouse two-cell to four-cell embryos. As mentioned above, the results of the *LincGET* ChIP–seq revealed that *LincGET* binds LTR and LINE elements in the *Carm1* gene locus, which was confirmed by LongTarget (Fig. 3b, Extended Data Fig. 5a and Supplementary Table 4). These results suggest that *LincGET* binding to the *Carm1* gene locus depends on both sequence complementarity and HNRNPU.

We found that HNRNPU ablation increased the ESS of *Carm1* pre-mRNAs (Fig. 3i and Extended Data Fig. 5e). Considering that the ESS of *Carm1* pre-mRNAs increased when the endogenic *LincGET* was replaced by deletion mutants without the HNRNPU-binding domain (ΔD and ΔE in Fig. 2g and Extended Data Fig. 4d), the correct localization of paraspeckles to the *Carm1* gene locus guided by the *LincGET*–HNRNPU complex is essential for inhibition of ESS of *Carm1* pre-mRNAs.

### *LincGET* opens up *Carm1* gene locus by H3 arginine methylation

We reasoned that *LincGET* opens up a target gene locus by establishing H3 arginine methylation, given that we previously reported that *LincGET* physically binds to CARM1 and further increases the level of H3 arginine methylation^1^, which can open chromatin and activate gene expression^35,36^. Based on the CARM1-binding motif and asymmetrical histone H3 arginine 17 dimethylation (H3R17me2a) motif generated from the ChIP–seq data^37^, we found seven CARM1-binding or H3R17me2a sites in the *Carm1* gene locus, five of which located are in or near *LincGET*-binding peaks (Extended Data Fig. 5a). Moreover, we performed ChIP on H3R26me2 followed by qPCR after *LincGET* depletion or CARM1 inhibition. The results showed that all the *LincGET-*binding sites and all CARM1-binding or H3R17me2a sites are enriched for H3R26me2 modification (Extended Data Fig. 5a). In addition, we found that *LincGET* depletion or CARM1 inhibition resulted in deceased H3R26me2 modification in the sites in or near *LincGET*-binding peaks (Extended Data Fig. 5a). These results suggest that *LincGET* binds to the *Carm1* gene locus and further increases the level of H3 arginine methylation to open up the target gene locus.

### Comparison between *LincGET* speckle and *Neat1* paraspeckle

The above results show that *LincGET*, rather than *Neat1*, functions in the organization of paraspeckles to their roles in alternative splicing regulation in mouse two-cell and four-cell embryos. However, in tissues and cell lines, it is *Neat1* that functions in the assembly of paraspeckles to regulate alternative splicing. We wanted to determine the potential similarities and differences between *LincGET* and *Neat1*.

First, we wondered whether *LincGET* contains similar structures as *Neat1*. We identified sequence similarity in a sequence of about 200 nucleotides in length (Supplementary Fig. 2a,b), and found similarities in the RNA second structure between *LincGET* and *Neat1* (Supplementary Fig. 2b–f,Supplementary Table 5 and Methods). These results suggest that *LincGET* and *Neat1* contain similar structures.

**Extended Data Fig. 6:**
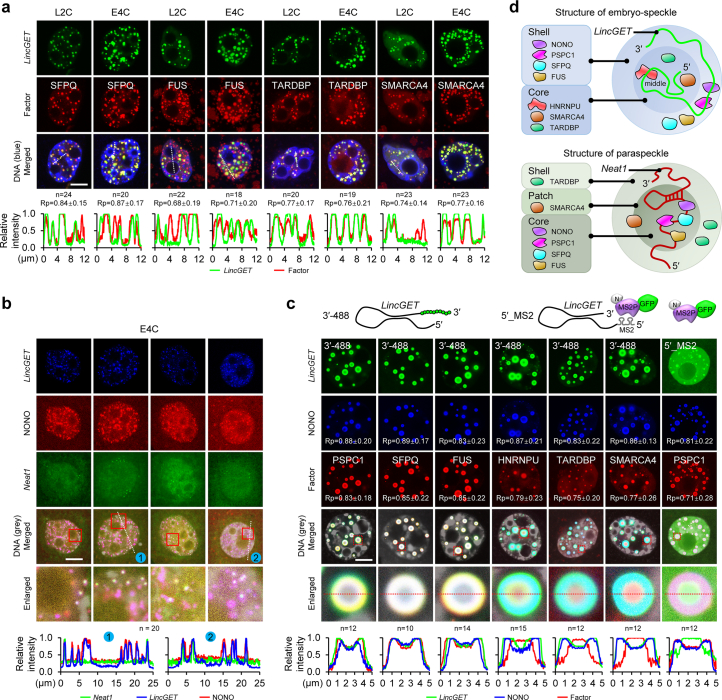
The structure of *LincGET-*speckle (embryo-speckle). **a**. Fluorescence images showing that *LincGET* co-localizes with SFPQ, FUS, TARDBP, and SMARCA4 in L2C and E4C embryos. Scale bar, 10 μm. The Rp values for co-localization of *LincGET* and SFPQ, FUS, TARDBP, or SMARCA4 are calculated by Fiji/ImageJ. Bottom panel, line scans of the relative fluorescence intensity of signals indicated by the dotted lines in top panel. **b**. Fluorescence images showing that *Neat1* co-localized with *LincGET* and NONO in mouse early four-cell embryos. Scale bar, 10 μm. Bottom panel, line scans of the relative fluorescence intensity of signals indicated by the dotted lines in top panel. **c**. Fluorescence images showing that the 3′-part of *LincGET*, NONO, PSPC1, SFPQ, and FUS locate in the shell, while the 5′-part of *LincGET*, HNRNPU, TARDBP, and SMARCA4 locate in the core of *LincGET*-speckles. Scale bar, 10 μm. The Rp values for co-localization of *LincGET* and NONO (shown on images of blue channel) as well as *LincGET* and PSPC1, SFPQ, FUS, HNRNPU, TARDBP, or SMARCA4 (shown on images of red channel) are calculated by Fiji/ImageJ. Top panel, illustration of the injected 3′-488 (3′-Alexa Fluor™ 488-labelled *LincGET*), and 5′-MS2 (5′-MS2-labelled *LincGET* combine GFP-tagged MS2P with nucleus location signal (N)). Note that the GFP-tagged MS2P with nucleus location signal doesn’t always bind to MS2-labelled *LincGET* and is dispersed throughout the nucleus. Bottom panel, line scans of the relative fluorescence intensity of signals indicated by the dotted line in top panel. **d**. Graphical representation of an *LincGET*-speckle (embryo-speckle) (top) and a *Neat1*-paraspeckle (bottom). Source data

Next, we wondered whether the *LincGET* speckles are similar to *Neat1* paraspeckles. We co-stained *LincGET* and other paraspeckle-essential components, including SFPQ, FUS, TARDBP and SMARCA4. The results showed the co-localization of *LincGET* with SFPQ, FUS, TARDBP and SMARCA4 in mouse late two-cell and early four-cell embryos (Rp ≈ 0.7; Extended Data Fig. 6a), suggesting that *LincGET* speckles are like the *Neat1* paraspeckles in protein components.

As both *LincGET* and *Neat1* bind to paraspeckle components, we wondered whether *Neat1* is involved in *LincGET* speckles. The co-staining result showed that there are only a few *Neat1* speckles in each nucleus of early four-cell embryos but are all co-stained with *LincGET* and NONO (Extended Data Fig. 6b), which suggests that *Neat1* can be involved in *LincGET-*guided paraspeckles. Using absolute qPCR, we found hundreds of copies of *LincGET* but fewer than ten copies of *Neat1* in one mouse late two-cell or early four-cell embryo (Supplementary Table 6), which might explain why *Neat1* ablation has only a slight effect on the assembly of paraspeckle components in mouse early four-cell embryos (Extended Data Fig. 3d).

We also explored the structure of *LincGET* speckles. The results showed that the 3′ part of *LincGET*, NONO, PSPC1, SFPQ and FUS is located in the shell, whereas the 5′ part of *LincGET*, HNRNPU, TARDBP and SMARCA4 is located in the core of *LincGET* speckles (Extended Data Fig. 6c). As HNRNPU binds the middle part of *LincGET* (Fig. 3f), the middle part of *LincGET* locates in the core of *LincGET* speckles (Extended Data Fig. 6d). NONO, PSPC1, SFPQ and FUS locate in the core while TARDBP and SMARCA4 locate in the shell of *Neat1* paraspeckles^38^, which suggests that *LincGET* speckles differ from *Neat1* paraspeckles in structure (Extended Data Fig. 6d).

### PCBP1 promotes ESS of *Carm1* pre-mRNAs

**Fig. 4:**
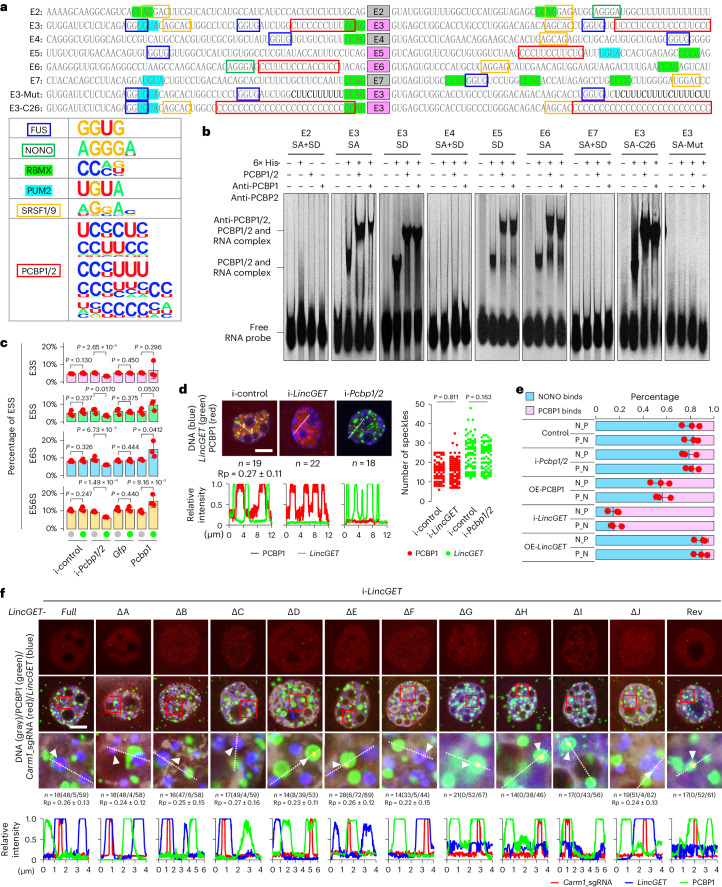
PCBP1 promotes ESS of *Carm1* which is inhibited by *LincGET*-guided paraspeckles. **a**, Protein-binding site analysis of splicing acceptor and donor flanking exons 2 to 7 of *Carm1* pre-mRNA. The sequences for E3-Mut and E3-C26 are shown. The known binding motifs are shown in the table (bottom left). **b**, RNA electrophoretic mobility shift assay analysis. Three biological replicates were performed. **c**, Combination charts showing changes in ESS of *Carm1* pre-mRNAs. Data are mean and s.e.m. One-tailed Student’s *t*-tests were used for statistical analysis (*n* = 3 biological replicates). **d**, Immunofluorescence combined with RNA-FISH assays in L2C. Scale bar, 10 μm. Bottom left, relative fluorescence intensity. Right, numbers of *LincGET* speckles and PCBP1 speckles calculated by Imaris. Two-tailed Student’s *t*-tests were used for statistical analysis. **e**, Assays of qPCR following sequential co-IP. Data are mean and s.e.m. One-tailed Student’s *t*-tests were used for statistical analysis and *P* values are shown in Extended Data Fig. 7f (three biological replicates). N_P, sequential co-IP by anti-NONO antibody and then anti-PCBP1 antibody; P_N, sequential co-IP by anti-PCBP1 antibody and anti-NONO antibody. **f**, Single nucleus of L2C injected with fluorescently labeled *LincGET* mutants, sgRNAs–dCas9 complex and PCBP1. White triangles indicate sgRNA signals. Scale bar, 10 μm. *n* = 18(48/5/59) means that 18 embryos were analyzed, 59 *Carm1* gene loci were detected, and 48 and 5 of them were covered by *LincGET* speckles and PCBP1, respectively. Bottom, relative fluorescence intensity. Rp values in **d** and **f** were calculated by Fiji/ImageJ. Source data

To further investigate the mechanism of the ESS of *Carm1* pre-mRNAs, we examined in detail the signature of splicing acceptor and splicing donor sequences around exons 2 to 7 of *Carm1* pre-mRNAs. Using RBPDB and CISBP-RNA databases, we found binding sites for FUS, NONO, RBMX, PUM2 and SRSF1/9 in splicing donor and acceptor elements around these exons (Fig. 4a). However, these binding sites are not specific to exons 3, 5 and 6, which are susceptible to be skipped during splicing. In addition, we noted that the splicing donor and acceptor elements flanking exons 3, 5 and 6, but not exons 2, 4 and 7, possess intronic C-rich motifs that could potentially be bound by PCBPs^39,40^ such as PCBP1 and PCBP2 (Fig. 4a), which are involved in alternative splicing^41–44^.

**Extended Data Fig. 7:**
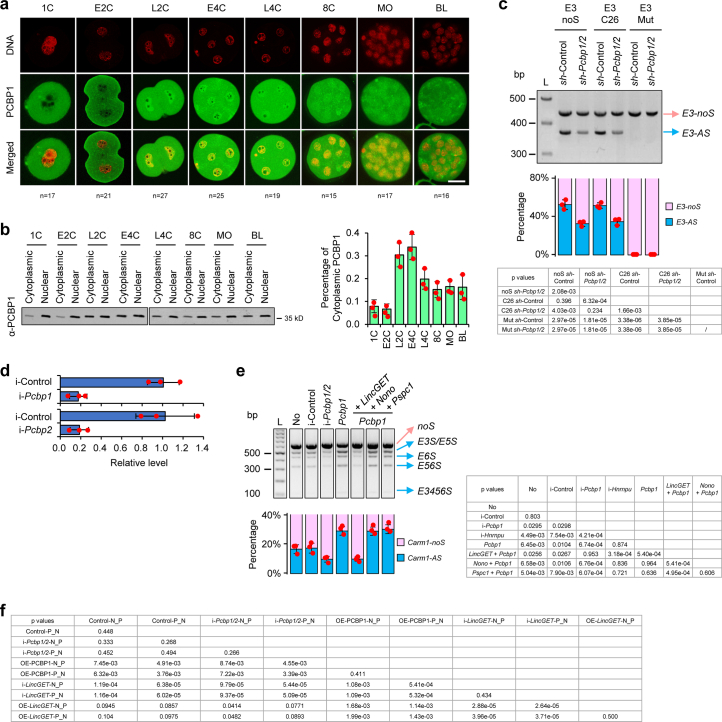
PCBP1 promotes ESS of *Carm1* via competitive binding to *Carm1* gene locus against *LincGET*-guided paraspeckles. **a**, **b**. The immunofluorescence results (**a**) and the western blotting results (**b**) of PCBP1 in mouse pre-implantation embryos, showing that PCBP1 was persistently expressed during mouse pre-implantation development, and PCBP1 was diffusely distributed in the cytoplasm and nucleus, but the nuclear localization is higher at late two- and early four-cell stages while is lower at one-cell and early two-cell stages than at other stages. The cytoplasm and nucleus are separated for western blotting. Three biological replicates were performed (**b**). Scale bar, 50 μm. α-, anti. Data are presented as mean values ± SEM (n = 3 biological replicates). kD, kilodalton. **c**. Top panel, splicing analysis using minigene containing exon 3 with flanked splicing acceptor and donor (SA and SD) sequences of *Carm1* pre-mRNAs. The splicing efficiency of exon 3 was assessed by RT-PCR. Middle panel, the percentages are shown in combination charts of bar plots and dot plots for top panel. Data are presented as mean values ± SEM. Bottom panel, one-tailed Student’s t-tests were used for statistical analysis and p values are shown in the table below (n = 3 biological replicates). L, DNA ladder. **d**. Interference efficiency analysis of siRNAs on *Pcbp1* and *Pcbp2*. Data are presented as mean values ± SEM (n = 3 biological replicates). **e**. Top left panel, agarose gel analysis of SPAR assays showing changes of ESS of *Carm1* pre-mRNAs upon *Pcbp1* overexpression or depletion. Bottom left panel, the percentages are shown in combination charts of bar plots and dot plots for top left panel. Data are presented as mean values ± SEM. Right panel, one-tailed Student’s t-tests were used for statistical analysis in bottom left panel and p values are shown in the table (n = 3 biological replicates). L, DNA ladder. No, group injected with nothing. **f**. The p values for Fig. 4e. One-tailed Student’s t-tests were used for statistical analysis (n = 3 biological replicates). Source data

We found that PCBP1 was persistently expressed during mouse pre-implantation development (Extended Data Fig. 7a). The results showed that PCBP1 was diffusely distributed in the cytoplasm and nucleus, but the nuclear localization is higher at late two-cell and early four-cell stages while is lower at one-cell and early two-cell stages than at other stages (Extended Data Fig. 7a,b). These results reflect a potential role of PCBP1 in regulating alternative splicing in mouse two-cell to four-cell embryos.

To investigate whether PCBP1/2 regulates alternative splicing of *Carm1* pre-mRNAs by directly binding the exons susceptible to be skipped during splicing, we performed RNA electrophoretic mobility shift assays. The results showed that PCBP1/2 binds to splicing acceptor and donor sequence-flanked exons 3, 5 and 6, which contain C-rich motifs, but not exons 2, 4 and 7, which do not contain C-rich motifs (Fig. 4b). In addition, we found that the binding activity of PCBP1/2 was slightly higher when the C-rich composition increased in the splicing acceptor sequence 5′ to exon 3 of *Carm1* (SA-C26 in Fig. 4b). However, no PCBP1 binding was seen when the sequence was modified from C-rich to U-rich (SA-Mut in Fig. 4b). These results confirm that PCBP1 prefers to bind C-rich motifs flanked by exons susceptible to be skipped in *Carm1* pre-mRNAs.

To test whether the binding of PCBP1 to intronic C-rich motifs is essential for ESS of *Carm1* pre-mRNAs, we cloned the *Carm1* exon 3 along with its flanked intronic C-rich motifs into a splice-reporter minigene^45^ and then transfected it into MCF-7 cells with PCBP1 and PCBP2 depletion (Supplementary Table 3). Given that PCBP1 and PCBP2 have extensive functional overlap, we co-depleted *Pcbp1* and *Pcbp2* (*Pcbp1/2*). As a result, we found that PCBP1 and PCBP2 co-depletion significantly repressed *Carm1* exon 3 skipping splicing, and no ESS occurred when the splicing acceptor sequence 5′ to exon 3 of *Carm1* was modified from C-rich to U-rich, even in the control group (Extended Data Fig. 7c). These data suggest that PCBP1 promotes ESS of *Carm1* pre-mRNAs by binding to the intronic C-rich motifs close to the splicing acceptor or donor sequences. Moreover, we found that *Pcbp1* overexpression increased and *Pcbp1* depletion decreased the ESS of *Carm1* pre-mRNAs in mouse four-cell embryos (Fig. 4c and Extended Data Fig. 7d,e). Together, these results indicate that PCBP1/2 binds *Carm1* pre-mRNAs and promotes its ESS.

### *LincGET* speckles prevent access of PCBP1 to *Carm1* gene locus

*Pcbp1* overexpression increased the ESS of *Carm1* pre-mRNAs but can be reversed by overexpression of *LincGET* (Extended Data Fig. 7e), reflecting competition between *LincGET* and PCBP1. Given that some lncRNAs can inhibit target protein function by physical binding^46^, we tested whether *LincGET* forms a complex with PCBP1. Based on immunostaining, we noted that both *LincGET* and PCBP1 formed speckles in the nucleus, but they were not co-localized and somewhat parallel to each other, both at two-cell and four-cell stages (Supplementary Fig. 3a), indicating that *LincGET* and PCBP1 are probably not in the same complex. In addition, we found that *LincGET* depletion did not affect the formation of PCBP1 speckles and vice versa (Fig. 4d and Supplementary Fig. 3b). These results indicate no interaction between PCBP1 and *LincGET*.

Splicing speckles have a crucial role, serving as storage and assembly sites for various pre-mRNA processing factors such as SRSF1 and the U2AF2 splicing factor^14,47^. Therefore, we asked whether *LincGET* or PCBP1 participate in splicing peckles. The staining results showed that PCBP1 co-localizes with SRSF1 in the nucleus of late two-cell and early four-cell embryos (Rp ≈ 0.6; Supplementary Fig. 3c), and the co-IP results revealed that PCBP1 forms complexes with SRSF1 in early four-cell embryos (Supplementary Fig. 3d), suggesting that PCBP1 is involved in splicing speckles. Additionally, *LincGET* does not co-localize with U2AF2 or SRSF1 (Supplementary Fig. 3e), and SRSF1 ablation did not affect the formation of *LincGET* speckles and vice versa (Supplementary Fig. 3f). These results suggest that PCBP1 participates in splicing speckles while *LincGET* is involved in the paraspeckles.

Considering that both PCBP1 and *LincGET* can bind to *Carm1* pre-mRNAs, we surmised that *LincGET*-guided paraspeckles affect the binding of PCBP1 to *Carm1* pre-mRNAs. To test our hypothesis, we performed sequential co-IP by anti-NONO antibody and then anti-PCBP1 antibody, as well as by anti-PCBP1 antibody and anti-NONO antibody (Supplementary Fig. 3g). The results showed that about 80% of *Carm1* pre-mRNA was captured by NONO in the control group, and about 45% and 85% of *Carm1* pre-mRNA was captured by PCBP1 upon PCBP1 overexpressing or upon *LincGET* depletion, respectively (Fig. 4e and Extended Data Fig. 7f). These results indicate that the affinity of *Carm1* pre-mRNA is higher for *LincGET*-guided paraspeckles than for PCBP1 in mouse four-cell embryos. We also tested the localization of PCBP1 when the localization of paraspeckles was disturbed, by replacing endogenic *LincGET* with *LincGET*-ΔD/ΔE mutants, or when paraspeckles were destroyed, by replacing endogenic *LincGET* with *LincGET*-ΔG/ΔH/ΔI mutants. We found that *LincGET* paraspeckles assembled around *Carm1* gene loci while the PCBP1 speckles were out of the *Carm1* gene loci when the full-length *LincGET* was injected (Fig. 4f and Supplementary Fig. 4a,b). However, the PCBP1 speckles assembled around *Carm1* loci both when the localization of paraspeckles was disturbed and when paraspeckles were destroyed (Fig. 4f and Supplementary Fig. 4a,c). These results indicate that *LincGET*-guided paraspeckles prevent access of PCBP1 to the *Carm1* gene locus to inhibit the ESS of *Carm1* pre-mRNAs.

### *LincGET* speckles occupancy links to *Carm1* ESS heterogeneity

**Extended Data Fig. 8:**
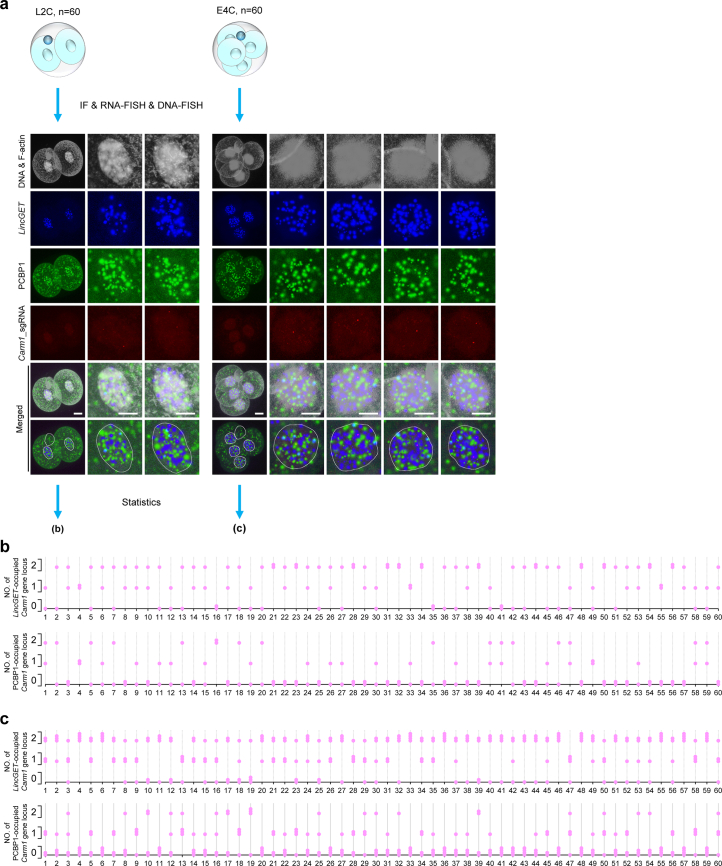
Analysis of the heterogenous occupancy of *LincGET*-speckles and PCBP1 around the *Carm1* gene loci. **a**. Illustration of the analysis of the heterogenous occupancy of *LincGET*-speckles and PCBP1 around the *Carm1* gene loci using immunofluorescence (IF) combined with RNA-FISH and DNA-FISH. Representative fluorescence images are shown which exhibit the occupancy of *LincGET*-speckles or PCBP1 around the *Carm1* gene loci in late two- and early four-cell embryos. Scale bar, 10 μm. DNA & F-actin, the nucleus and membrane are displayed together with SYTOX™ Deep Red Nucleic Acid Stain (Invitrogen, S11380) and Alexa Fluor™ 647 Phalloidin (Invitrogen, A22287) to show the three-dimension structure of embryos. **b, c**. The dot plots showing the counting results of the number (NO.) of *LincGET-*occupied or PCBP1-occupied *Carm1* gene loci in late two- (**b**) and early four-cell embryos (**c**). To prevent the dots from overlapping completely, the y-axis is divided into three groups with NO. = 0, 1, and 2, respectively. Source data

Given that the above results indicated that the competitive occupancy of *LincGET* speckles and PCBP1 regulates skipping splicing of exons 3 to 6 of *Carm1* pre-mRNAs, we wondered whether the occupancy of *LincGET* speckles and PCBP1 around the *Carm1* gene locus is heterogenous in mouse two-cell and four-cell embryos and whether the heterogenous occupancy is associated with the heterogeneity of exon-skipping events on exon 3 to 6 of *Carm1* pre-mRNAs. Interestingly, the immunofluorescence combined with RNA-FISH and DNA-FISH results supported the heterogenous occupancy of *LincGET* speckles and PCBP1 around the *Carm1* gene loci in mouse late two-cell and early four-cell embryos (Extended Data Fig. 8a–c). Simultaneous detection of the heterogenous occupancy and the heterogeneity of *Carm1* ESS events in single blastomeres is very difficult because RNA degradation is severe after immunofluorescence combined with RNA-FISH and DNA-FISH and there are no instruments capable of microdissection in three dimensions.

**Fig. 5:**
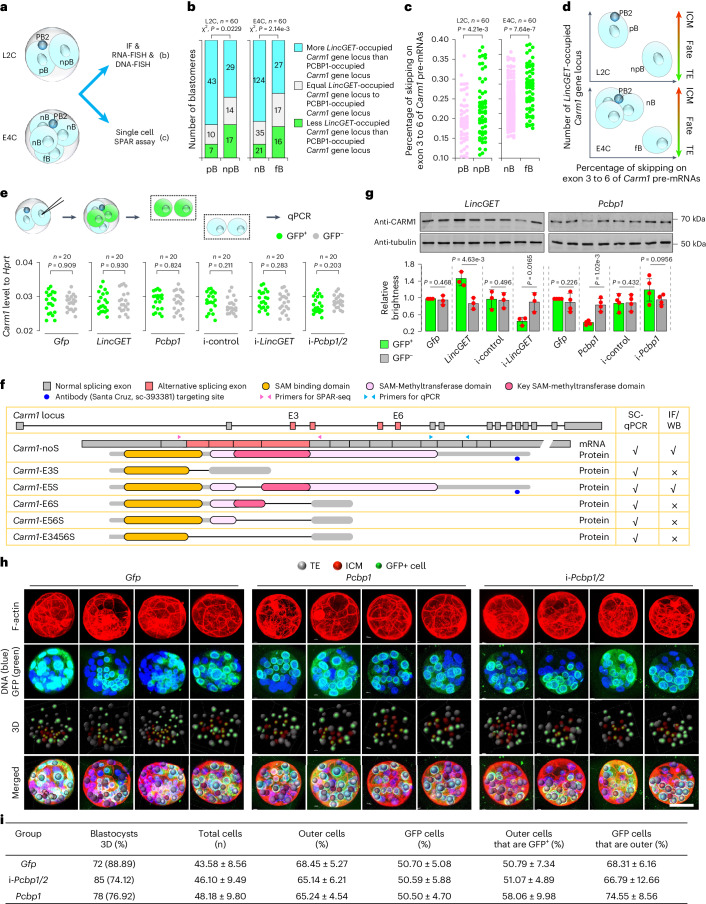
Alternative splicing contributes to CARM1 heterogeneity. **a**–**c** Illustration of the heterogenous analysis (**a**) using immunofluorescence combined with RNA-FISH and DNA-FISH (**b**) and single-cell SPAR assay (**c**). pB and npB denote the blastomeres attached (pB) or not attached (npB) by the second polar body (PB2) in two-cell embryos; nB and fB denote the blastomeres near (nB) or farthest (fB) from PB2 in tetrahedral four-cell embryos. **b**, Histogram showing the heterogenous occupancy of *LincGET* speckles and PCBP1 around the *Carm1* gene loci according to the spatial position relative to PB2 in L2C and E4C. *χ*^2^ tests are used for statistical analysis with df = 2; *n* = 60 L2C or E4C examined over four biological replicates. **c**, Dot plots showing the heterogeneity of ESS of *Carm1* pre-mRNAs according to the spatial position relative to PB2 in L2C and E4C. Two-tailed Student’s *t*-tests are used for statistical analysis. *n* = 60 L2C or E4C examined over four biological replicates. **d**, Illustration of the correlation between heterogeneous occupancy of *LincGET* speckles around the *Carm1* gene loci and the heterogeneity of ESS of *Carm1* pre-mRNAs. **e**, Single-cell qPCR assays in E4C. Two-tailed Student’s *t*-tests were used for statistical analysis. **f**, ESS events on exons 3 to 6 of *Carm1* pre-mRNAs and the corresponding protein structures. Primer sites and antibody-recognizing sites are shown. IF, immunfluorescence; WB, western blot. **g**, Western blot assays in E4C. Tubulin is used as a control. Data are mean and s.e.m. One-tailed Student’s *t*-tests were used for statistical analysis (left, *n* = 3; right, *n* = 4 biological replicates). **h**, Examples of three-dimensional (3D) reconstruction analysis. Scale bar, 50 mm. TE, trophectoderm; ICM, inner cell mass. **i**, Analysis of the distribution of progeny of injected blastomere at the blastocyst stage based on 3D reconstruction. Data are mean ± s.e.m. Two-tailed Student’s *t*-tests were used for statistical analysis and *P* values are shown in Extended Data Fig. 10d. Key to table headings are shown in Supplementary Table 8. Source data

**Extended Data Fig. 9:**
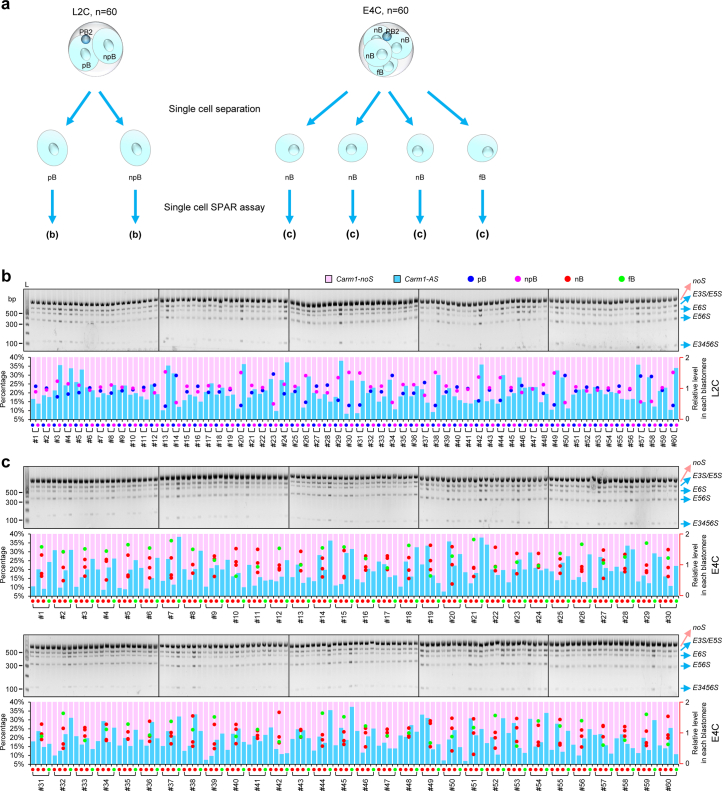
Analysis of the heterogeneity of exon 3 to 6 skipping events of *Carm1* pre-mRNAs. **a**. Illustration of the analysis of the heterogeneity of exon 3 to 6 skipping events of *Carm1* pre-mRNAs via single-cell SPAR assay. **b, c**. Agarose gel analysis of single-cell SPAR assay showing the heterogeneity of exon-skipping events on exon 3 to 6 of *Carm1* pre-mRNAs in late two-cell (**b**) and early four-cell (**c**) embryos. The percentages are shown in bar plots below the gel. L, DNA ladder. Note that the embryos #1 to #24 of both L2C and E4C are also used in the results for exon 3 to 6 skipping splicing in Extended Data Fig. 2b, but the spatial position information are shown here with different colors for pB and npB as well as nB and fB, but not in Extended Data Fig. 2b. Source data

We then used the spatial position relative to the second polar body (PB2) as a link to study the relationship between the heterogenous occupancy and the heterogeneity of *Carm1* ESS events for two reasons. Firstly, the spatial position of blastomeres in the two-cell to four-cell stage can be recorded by their positions relative to PB2. Blastomeres in two-cell embryos can be recorded as blastomeres that are attached by PB2 (pB) and not attached by PB2 (npB); blastomeres in tetrahedral four-cell embryos can be recorded as blastomeres that are near PB2 (nB) and far from PB2 (fB) (Fig. 5a). Secondly, pB and npB as well as nB and fB have a cell fate bias but not a random fate^24,48^. As a result, we found that the *Carm1* gene loci are more likely to be occupied by *LincGET* speckles, and the percentage of exon-skipping events on exon 3 to 6 of *Carm1* pre-mRNAs is lower in pB than in npB in late two-cell embryos, and lower in nB than in fB in tetrahedral late four-cell embryos (Fig. 5b,c, Extended Data Fig. 9a–c and Supplementary Table 7). These results demonstrate that the more *LincGET* speckles there are around the *Carm1* gene loci, the fewer ESS events there are on exon 3 to 6 of *Carm1* pre-mRNAs (Fig. 5d).

### Alternative splicing contributes to CARM1 heterogeneity

**Extended Data Fig. 10:**
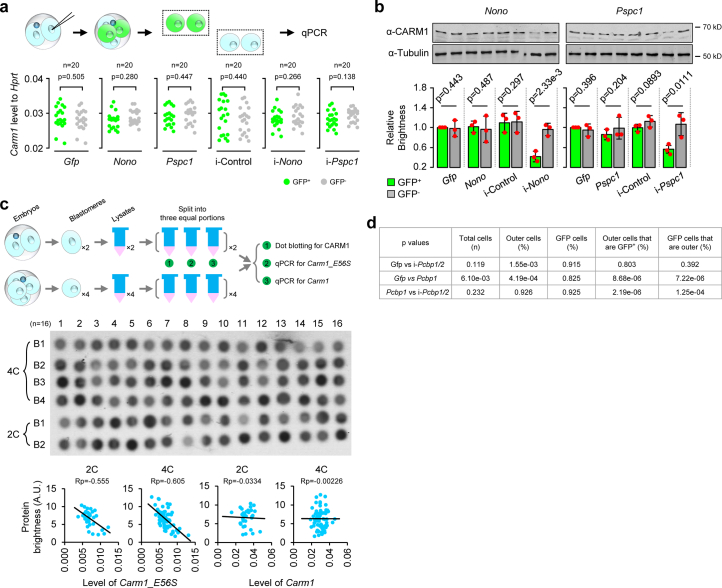
NONO and PSPC1 regulate CARM1 protein level but not transcription level. **a**. Single-cell qPCR results showing neither *Nono* nor *Pspc1* affects the transcription level of *Carm1*. Two-tailed Student’s *t*-tests were used for statistical analysis. **b**. Western blot results showing that *Nono* or *Pspc1* depletion downregulates CARM1 at the protein level. Tubulin is used as a control. Data are presented as mean values ± SEM (n = 3 biological replicates). Two-tailed Student’s *t*-tests were used for statistical analysis. ns, *p* > 0.05. α-, anti. kD, kilodalton. **c**. The ESS level but not the expression level of *Carm1* correlates with CARM1 protein level. The protein brightness from dot blotting is calculated by Fiji/ImageJ. Middle, results of dot plotting. Bottom, the relationship between protein brightness and expression level of the ESS isoform *Carm1_E56S* (Bottom left) or the total expression level of *Carm1* (Bottom right) is shown in the dot plot. The Rp values were calculated in Excel. **d**. The p values for Fig. 5i. One-tailed Student’s t-tests were used for statistical analysis (n = 3 biological replicates). Source data

Given that ESS can lead to decreased protein levels^49^, we wondered whether ESS contributes to CARM1 protein heterogeneity in mouse early embryos. We found that neither overexpression nor depletion of *Nono*, *Pspc1*, *LincGET* or *Pcbp1* affects *Carm1* mRNA levels (Fig. 5e and Extended Data Fig. 10a), reflecting that neither *LincGET*-paraspeckles nor PCBP1 are involved in transcriptional regulation of *Carm1*. However, we found that depletion of *Nono*, *Pspc1* and *LincGET* and overexpression of *Pcbp1* decreased while overexpression of *LincGET* increased the CARM1 protein level (Fig. 5f,g, Extended Data Fig. 10b). These results were consistent with the previous observation that knockdown of paraspeckle components (NONO) decreased CARM1 protein levels in mouse early embryos^3^. To further explore whether the ESS is correlated with CARM1 protein level, single blastomeres separated from late two-cell and early four-cell embryos were used for dot plotting for CARM1 and qPCR for the ESS isoform *Carm1_E56S* and the total expression level of *Carm1*. The results showed that the ESS level but not the expression level of *Carm1* correlates with CARM1 protein levels in mouse two-cell to four-cell embryos (Extended Data Fig. 10c). Together, our findings indicate that both *LincGET* speckles and PCBP1 regulate CARM1 protein levels through the regulation of ESS.

As PCBP1 promotes ESS of *Carm1* pre-mRNAs, which contrasts with the role of *LincGET*, we wondered whether PCBP1 overexpression biases first cell fate toward the trophectoderm as opposed to *LincGET*, which biases blastomeres toward inner cell mass^1^. The total number of cells and the percentage of GFP^+^ cells in blastocysts were similar among *Pcbp1* overexpression, *Pcbp1/2* ablation and the control groups (Fig. 5h,i, Extended Data Fig. 10d and Supplementary Table 8), indicating that *Pcbp1* overexpression or depletion does not affect the overall mouse pre-implantation development. However, the percentage of outer cells that are GFP^+^ was significantly higher in the *Pcbp1* overexpression group (58.06 ± 9.98%) than in the control group (50.79 ± 7.34%) and *Pcbp1* ablation group (51.07 ± 4.89%), and the percentage of GFP^+^ cells that were outer was also significantly higher in the *Pcbp1* overexpression group (74.55 ± 8.56%) than in the control group (68.31 ± 6.16%) and *Pcbp1* ablation group (66.79 ± 12.66%) (Fig. 5h,i, Extended Data Fig. 10d and Supplementary Table 8). These results reflect that overexpression of PCBP1 in one of the two-cell blastomeres leads to decreased CARM1 protein levels and biases its progeny cells toward a trophectoderm fate.

## Discussion

**Fig. 6:**
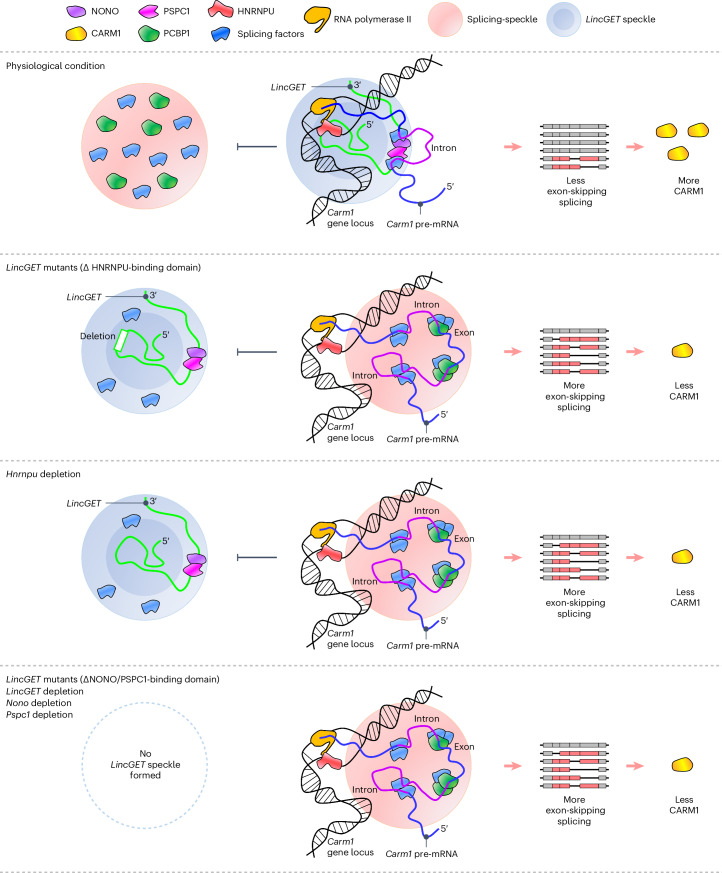
Model of *LincGET*-guided paraspeckles and PCBP1 on ESS regulation of *Carm1* pre-mRNAs. In physiological conditions (first line), *LincGET* speckles assemble around the *Carm1* gene locus and inhibit the access of splicing speckles, leading to less ESS of *Carm1* pre-mRNAs and more CARM1 protein. When *LincGET* loses the HNRNPU-binding domain (second line) or without HNRNPU (third line), *LincGET* speckles assemble but cannot localize to the *Carm1* gene locus, while splicing speckles assemble around the *Carm1* gene locus, leading to more ESS of *Carm1* pre-mRNAs and less CARM1 protein. When *LincGET* loses the NONO/PSPC1-binding domain, or without *LincGET*, *Nono* or *Pspc1* (fourth line), *LincGET* speckles cannot assemble, while splicing speckles assemble around the *Carm1* gene locus, leading to more ESS of *Carm1* pre-mRNAs and less CARM1 protein.

CARM1 heterogeneity is the core factor that biases the first cell fate in mice, yet the mechanism by which CARM1 heterogeneity is established is far from understood. Here, we found that *LincGET* contributes to CARM1 heterogeneity at the post-transcriptional level by tethering paraspeckle components to the *Carm1* gene locus to inhibit the ESS of *Carm1* pre-mRNAs (Fig. 6). In light of our earlier work^1^, our findings reflect that *LincGET* both helps to correctly splice *Carm1* mRNA and, after CARM1 is translated, *LincGET* binds CARM1 to regulate epigenetic modifications and cell fate bias as early as the two-cell stage in mice. Moreover, we found that PCBP1 promotes the ESS of *Carm1* pre-mRNAs and biases the blastomere towards a trophectoderm fate. This study demonstrates that alternative splicing contributes to the first cell fate bias in mouse pre-implantation embryos.

There are other non-membrane-bound subnuclear domains that function in RNA processing, such as the nuclear poly(A) domains (NPADs) that work as the hub for newly synthesized mRNA in growing mouse oocytes^50^. In addition to nuclear poly(A)-binding protein 1 (PABPN1), NPADs also involve alternative splicing factors such as SRSF2 (also known as SC35)^50^. Although *LincGET* is not expressed in metaphase II (MII) oocytes^2^, it is a worthwhile topic in the future to explore whether paraspeckles and/or splicing speckles exist and function in the growing oocytes when transcription is still active. If this is the case, whether or not the protein components of the NPAD, such as PABPN1 and SRSF2, co-localize with paraspeckles and/or splicing speckles would require further study.

## Methods

### Ethical statements

All the mouse procedures were carried out in compliance with the guidelines of the Animal Care and Use Committee of the Institute of Zoology, Chinese Academy of Sciences, and the Animal Care and Use Committee of the Northeast Agricultural University.

### Antibodies

The following antibodies were used for immunoblotting, immunostaining and/or immunoprecipitation: mouse monoclonal (G-2) anti-CARM1 (Santa Cruz, sc-393381), chicken polyclonal anti-GFP (Abcam, ab13970), rat monoclonal (YL1/2) anti-Tubulin (Abcam, ab6160), goat polyclonal anti-PCBP1 (Abcam, ab109577), rat monoclonal (EPR14859(2)) anti-PCBP2 (Abcam, ab200835), rat monoclonal (EPR8239) anti-SRSF1 (Abcam, ab129108), rabbit polyclonal anti-HA (Abcam, ab9110), rabbit polyclonal anti-NONO (Abcam, ab70335), rabbit polyclonal anti-PSPC1 (Abcam, ab104238), rabbit polyclonal anti-HNRNPU (Abcam, ab20666), rabbit polyclonal anti-U2AF2 (Abcam, ab37530), rabbit polyclonal anti-H3R26me2 (Abcam, ab127095), FITC-conjugated donkey anti-Chicken IgY polyclonal secondary antibody (Invitrogen, SA1-72000), Alexa Fluor Plus 555-conjugated goat anti-Mouse IgG polyclonal secondary antibody (Invitrogen, A32727), Alexa Fluor Plus 555-conjugated donkey anti-Goat IgG polyclonal secondary antibody (Invitrogen, A32816), Alexa Fluor Plus 555-conjugated donkey anti-Rabbit IgG polyclonal secondary antibody (Invitrogen, A32794), Alexa Fluor Plus 555-conjugated donkey anti-Rat IgG polyclonal secondary antibody (Invitrogen, A48270), Alexa Fluor 488-conjugated rabbit anti-Rat IgG polyclonal secondary antibody (Invitrogen, A-21210), HRP-conjugated goat anti-Rabbit IgG polyclonal secondary antibody (Invitrogen, A27036), HRP-conjugated mouse anti-Goat IgG polyclonal secondary antibody (Invitrogen, 31400) and HRP-conjugated goat anti-Rat IgG polyclonal secondary antibody (Invitrogen, 31470).

### Mouse embryo collection

The CD1 (ICR) mice were purchased from Vital River company. All mice used for experiments were 7–8 weeks old. All mice were housed in the animal care facilities under specific pathogen-free conditions, with a 12 h dark–light cycle, ambient temperature ranging from 21 °C to 26 °C and a humidity level of 50% to 60%. To obtain pre-implantation embryos, female mice were injected with 10 U of pregnant mare serum gonadotropin (PMSG, Prospec, HOR-272) and 10 U of human chorionic gonadotropin (hCG, Prospec, HOR-250) at 46–48 h intervals and then crossed with 7–8-week-old CD1 (ICR) male mice. Embryos were collected at the following times post hCG injection: early one-cell stage (phCG 19 h), late one-cell stage (phCG 30 h), early two-cell stage (phCG 39 h), late two-cell stage (phCG 48 h), early four-cell stage (phCG 54 h), late four-cell stage (phCG 62 h), early eight-cell stage (phCG 68 h), late eight-cell stage (phCG 74 h), 16-cell stage (phCG 80 h), 32-cell stage (phCG 90 h), early blastocyst stage (phCG 98 h) and late blastocyst stage (phCG 114 h).

### Culture cells

mEpiSCs were established in Q. Zhou’s lab in the State Key Laboratory of Stem Cell and Reproductive Biology, Institute of Zoology, Chinese Academy of Sciences, and cultured in a fibronectin-coated dish in N2B27 medium plus 12 ng ml^−1^ bFGF (R&D, 233-FB-001MG/CF) and 10 ng ml^−1^ activin A (R&D, 338-AC-01M) at 37 °C in a 5% CO_2_ incubator. The culture medium was changed every day, and the mEpiSCs were passaged every 2–3 days and then digested to single cells by 0.05% trypsin/EDTA (GIBCO, 25300062). The MCF-7 cells were purchased from ATCC (ATCC, HTB-22). MCF-7 cells were cultured in DMEM/F-12 (1:1) medium supplemented with 10% FBS at 37 °C in a 5% CO_2_ incubator. For cell transfection, the cells were passaged and seeded at a density of 1–1.5 × 10^4^ cells per cm^2^. After 2 days (60–70% confluence), plasmid DNA (MS2-LincGET and MS2P-HA for mEpiSCs; PCBP-shRNA for MCF-7 cells) was transferred into cells using Lipofectamine 3000 transfection reagent (GIBCO, L3000015) according to the manufacturer’s instructions. mEpiSCs and MCF-7 cells were collected for further analysis 36 and 72 h after transfection, respectively.

### RNA extraction, reverse transcription and qPCR

RNA was extracted using the RNeasy Mini Kit (QIAGEN, 74104), and the RNase-Free DNase Set (QIAGEN, 79254) was used to prevent DNA contamination. Reverse transcription was performed using a High-Capacity cDNA Reverse Transcription Kit (ABI, 4368814). SYBR-qPCR was performed using a Power SYBR Green PCR Master Mix (ABI, 4367659). TM-qPCR was performed using TaqMan Universal Master Mix II (Life, 4440048). All kits were used following the manufacturers’ guidelines.

### Construction of plasmid vectors

To create Alexa Fluor 488-labeled RNA probes for *LincGET* RNA-FISH, the specific *LincGET* region (2,574–2,763) was amplified using the 2× Vazyme Lamp Master Mix (Dye Plus) (Vazyme, P312). Primers are shown in Supplementary Table 3. These sequences were sub-cloned into the plasmid pEASY-T3 cloning vector (TransGen, CT301-02), which contains the T7 promoter. For co-IP experiments, the MS2 coat protein (MS2P), MS2-labeled *LincGET* and HA-labeled MS2P were cloned into the PB533A vector (SBI, PB533A-2) digested with *EcoR*I or *Sal*I, respectively.

For in vitro transcription, the GFP-KASH sequence with T7 promoter was synthesized by BGI company and sub-cloned into the pUC57 vector; *LincGET* mutants were generated by PCR, 5′-phosphorylation and ligation; plasmid containing NONO-BFP or NONO-mCherry with T7 promoter was purchased from YouBio company (YouBio, L3774 and L2772).

For coding potential analysis for exon-skipping variants of *Carm1*, *Carm1* (NM_021531) cDNA for non-skipping splicing (noS) and *Carm1* (NM_153141) cDNA for the E15S variant were purchased from YouBio company (YouBio, G156971 and G156972). The EF1α promoter from Addgene plasmid no. 61422, *Carm1* cDNA and bGH poly(A) signal element from Addgene plasmid no. 61422 were sub-cloned into plasmid pEGFP-C1, purchased from YouBio company (YouBio, VT1118), using NEBuilder HiFi DNA Assembly Cloning Kit (NEB, E5520S). *Carm1* mutants of E3S, E5S, E6S, E56S, E3456S and E11S were generated by PCR, 5′-phosphorylation and ligation.

For short hairpin RNA (shRNA) expression, primers containing sense-loop–antisense structure (Supplementary Table 3) were synthesized and annealed to generate double-strand oligonucleotides with a re-annealing process: 95 °C for 10 min; 95–85 °C ramping at −2 °C s^−1^; 85–25 °C at −0.25 °C s^−1^; and 25 °C hold for 1 min. Then the double-strand oligonucleotides were sub-cloned into the pLL3.7 vector (Addgene, 11795).

For protein expression and purification, the NONO, PCBP1 or PCBP2 sequence followed by a 14 amino acid linker sequence ‘GAPGSAGSAAGGSG’ and mCherry or GFP was cloned into a modified version of a T7 pET expression vector (YouBio) containing a 5′ MBP tag and a 3′ 6×His tag.

For minigene analysis of ESS of *Carm1* pre-mRNAs, wild-type *Carm1* exon 3 flanked by a 539 bp 5′ splicing acceptor sequence and 258 bp 3′ splicing donor sequence, wild-type *Carm1* exon 5 flanked by a 531 bp 5′ splicing acceptor sequence and 506 bp 3′ splicing donor sequence, and wild-type *Carm1* exon 6 flanked by a 550 bp 5′ splicing acceptor sequence and 418 bp 3′ splicing donor sequence were amplified by PCR with the primers listed in Supplementary Table 3 using Q5U Hot Start High-Fidelity DNA Polymerase (NEB, M0515) and then cloned into the KpnI-digested pSpliceExpress (Addgene, 32485) splicing minigene plasmid^45^ using the In-Fusion HD Cloning Kit (Clontech, 639636) following the manufacturers’ guidelines. Plasmids for E3-C26 and E3-Mut were generated by PCR with primers containing mutations using Q5U Hot Start High-Fidelity DNA Polymerase (NEB, M0515), phosphorylation using T4 polynucleotide kinase (NEB, M0201L) and ligation using T4 DNA ligase (NEB, M0202L).

### Micro-injection

Mouse embryos at the one-cell stage were collected and microinjected with about 1–2 pL RNA at 150 ng μl^−1^ concentration or plasmid at 10 ng μl^−1^ concentration into the pronucleus at phCG 25 h, using an Eppendorf micromanipulator on a Nikon inverted microscope. The *LincGET* and mRNAs for NONO and PCBP1 were in vitro transcribed with HiScribe T7 ARCA pre-mRNAs Kit (with tailing) (NEB, E2060), while the single-guide RNAs (sgRNAs) were in vitro transcribed with HiScribe T7 Quick High Yield RNA Synthesis Kit (NEB, E2050) following the manufacturers’ guidelines. DNA templates with T7 promoter were amplified using the 2× Vazyme Lamp Master Mix (Dye Plus) (Vazyme, P312). Primers are shown in Supplementary Table 3. The fluorescently labeled *LincGET* or sgRNAs were generated by Poly(U) Polymerase (NEB, M0337S) with modified nucleotide 5-(3-aminoallyl)-UTP (Ambion, AM8437) and then labeled with Alexa Fluor 488 NHS Ester (Succinimidyl Ester) (Invitrogen, A20000) or Alexa Fluor 546 NHS Ester (Succinimidyl Ester) (Invitrogen, A20002) following the manufacturers’ guidelines. Locked nucleic acid (LNA) or short interfering RNA (siRNA) was used in co-injection, in the following concentrations: 10 μM LNA for control; 1 μM LNA for *LincGET*; and 10 μM siRNA for *Nono*, *Pspc1*, *Hnrnpu* or control.

### Western blot

For each lane, 200 single blastomeres from embryos were lysed with RIPA lysis buffer (10 μl per lane; Beyotime, P00138) containing 1 mM phenylmethyl sulfonyl fluoride (PMSF; Beyotime, ST506), mixed with 30 μl of sample buffer (Beyotime, P0283) and incubated for 5 min in boiling water bath. The samples were then separated by SDS–PAGE with a 5% stacking gel (10 ml; 5.7 ml ddH_2_O, 1.7 ml 30% acrylamide (29:1), 100 μl of 10% SDS, 2.5 ml 1.5 M pH 6.8 Tris-HCl, 50 μl of 10% ammonium persulfate and 10 μl of TEMED) and a 10% separating gel (10 ml; 4.1 ml ddH_2_O, 2.5 ml 1.5 M pH 8.8 Tris-HCl, 3.3 ml 30% acrylamide (29:1), 100 μl of 10% SDS, 50 μl of 10% ammonium persulfate and 5 μl of TEMED) at 100 V for 1 h. Separated proteins were then electrophoretically transferred onto a nitrocellulose membrane at 250 mA for 1 h at 4 °C. Membranes were then blocked in TBST buffer (150 mM NaCl, 10 mM Tris, 0.1% Tween-20, pH 7.4) containing 3% BSA (Sigma, B2064), for 1 h at room temperature and then incubated with primary antibody, diluted (mass:volume ratio, 1:200) in TBST containing 1% BSA, overnight at 4 °C. After three washes for 10 min each in TBST, membranes were incubated for 1 h at room temperature with the fluorescence-labeled secondary antibody diluted (mass:volume ratio, 1:5,000) in TBST containing 1% BSA. After three washes for 10 min each, the membrane was exposed to a Bio-Rad GelDoc XR+ Gel imaging system for the acquisition of signals.

### Immunoprecipitation

Magnetic Beads Protein G were coated with 5 μg of primary antibody in RIP wash buffer (150 mM sodium chloride, 50 mM Tris-HCl, pH 7.4, 1 mM MgCl_2_ and 0.05% NP-40) containing 5% BSA overnight with rotation at 4 °C. Then, we collected approximately 1 × 10^6^ mEpiSCs expressing HA-MS2P, with or without MS2-*LincGET*, added to 100 μl of RIP lysis buffer (50 mM Tris-HCl pH 7.4, 150 mM NaCl, 1 mM EDTA pH 8.0 (Invitrogen, AM9260G), 1% NP-40, 0.1% SDS, 0.5% sodium deoxycholate, 0.5 mM DTT, 1 mM PMSF/cocktail) and 10 μl of RNase inhibitor (Ambion, AM2694) and incubated on ice for 10 min. For embryos injected with MS2-*LincGET* or MS2-*anti-LincGET*, pre-mRNAs for HA-MS2P and pre-mRNAs for NONO, about 400 embryos were used for each group. Next, we centrifuged the RIP lysate at 10,000×*g* for 10 min at 4 °C, removed 100 μl of the supernatant and added this to 900 μl of the beads–antibody complex (mass:volume ratio, 1:200) in RIP Immunoprecipitation Buffer (860 μl RIP wash buffer, 35 μl 0.5 M EDTA pH 8.0 and 5 μl RNase inhibitor) and incubated this with rotation overnight at 4 °C. The residual 10 μl of the supernatant of RIP lysate was treated as input. After washing, the immunoprecipitate was divided into two parts. One part was mixed with 15 μl of western blot sample buffer and incubated for 5 min in boiling water; this blot was used to detect HA-MS2P and NONO/PSPC1/HNRNPU (according to the antibody). The other part was treated with proteinase K at 55 °C for 30 min with shaking to digest the protein, followed by RNA extraction from the supernatant; reverse transcription polymerase chain reaction (RT–PCR) was then performed to detect *LincGET*.

### Immunofluorescence staining

Mouse embryos were permeabilized in 1% Triton X-100 (Sigma, T9284) in 1× PBS (Invitrogen, AM9625) and fixed in ice-cold 100% methanol for 30 min at −20 °C, followed by permeabilization again in 70% ethanol for 30 min at 4 °C. Then, embryos were blocked in blocking solution (1% BSA in 1× PBS) for 1 h at room temperature after three washes for 5 min each in washing solution (0.1% Tween-20, 0.01% Triton X-100 in 1× PBS), followed by incubation with primary antibody diluted (mass:volume ratio, 1:50) with blocking solution overnight at 4 °C. After three washes, embryos were incubated with Alexa series fluorescent tag-conjugated secondary antibody diluted (mass:volume ratio, 1:1,000) with washing solution for 1 h at room temperature. After three washes, embryos were mounted with DAPI-Vectashield solution (Vector laboratories, H1200). Notably, when the embryos were injected with Alexa Fluor 488-labeled *LincGET* and pre-mRNAs for NONO-mCherry, the embryos were directly mounted with DAPI-Vectashield solution (Vector laboratories, H1200) after permeabilization. When the embryos were injected with Alexa Fluor 488-labeled *LincGET*, pre-mRNAs for NONO-BFP and Alexa Fluor 546-labeled sgRNAs, the embryos were directly stained with SYTOX Deep Red Nucleic Acid Stain (Invitrogen, S11380) and then mounted after permeabilization. Imaging of embryos was then performed using a laser-scanning confocal microscope (Leica, TCS SP8). IMARIS software (Bitplane) was then used to calculate the number of speckles in each picture.

### Immunofluorescence combined with RNA-FISH

Probes targeting *LincGET* used in RNA-FISH were labeled by in vitro transcription using the MEGAshortscript Kit (Ambion, AM1354) with ATP, CTP, GTP, UTP and ChromaTide Alexa Fluor 488-5-UTP (Invitrogen, C11403) solution (4:4:4:1:4) in which 80% of uracil was labeled by Alexa Fluor 488. The zona pellucida of the mouse embryos was removed with incubation in acidic Tyrode’s Solution (Sigma, T1788). Then the embryos were incubated in PBSA (1× PBS containing 6 mg ml^−1^ BSA) for 3 min and transferred onto Superfrost/Plus microscope slides and dried as quickly as possible (less than 5 min). Embryos were permeabilized in 1% Triton X-100 in 1× PBS and fixed in ice-cold 100% methanol for 30 min at −20 °C. Then, slides were transferred into 70% ethanol on ice for 20 min. To perform IF, slides were rinsed in PBS for 5 min, blocked in blocking buffer (1× PBS with 0.1% Tween-20, 1% BSA and 0.4 U μl^−1^ of Ribonuclease Inhibitor (Invitrogen, 10777019)) for 20 min at room temperature, and then incubated with the primary antibody (mass:volume ratio, 1:50) in blocking buffer for 1 h at room temperature. After three washes with 0.1% Tween-20 in PBS, slides were incubated with secondary antibody (mass:volume ratio, 1:1,000) in blocking buffer for 1 h at room temperature. After three washes with 0.1% Tween-20 in PBS, slides were post-fixed in ice-cold 100% methanol for 30 min at −20 °C. The slides were then transferred into 70% ethanol on ice. Dehydration was performed in 80%, 95% and 100% ethanol (×2), with each incubation lasting for 5 min at room temperature. Slides were then dried for 5 min. Embryos were then hybridized in the hybridization solution (50% Formamide (Sigma, F9037), 1% Dextran Sulfate (Sigma, 30915), 2× SSC (Sigma, S6639-1L), 10 mM VRC (Sigma, 94742), 2 mg ml^−1^ BSA) containing 5 μg of Alexa Fluor 488-labeled RNA probes per slide at 37 °C overnight (14–15 h). After three washes for 5 min each in hybridization washing solution (50% formamide, 2× SSC) at 42 °C and four washes for 5 min each in 2× SSC, embryos were mounted with DAPI-Vectashield solution (Vector laboratories, H1200). Fluorescence staining was imaged using a laser-scanning confocal microscope (Leica, TCS SP8). IMARIS software (Bitplane) was then used to calculate the number of speckles for each picture.

### DNA-FISH combined with RNA-FISH

For the preparation of probes in DNA-FISH, 17 DNA fragments containing T7 promoter (2,100–2,700 bp) were amplified from the mouse genome using LongAmp *Taq* DNA Polymerase (NEB, M0534L). Primers are given in Supplementary Table 3. Then, in vitro transcription was performed with mixed 17 DNA fragments using the MEGAshortscript Kit (Ambion, AM1354) with ATP, CTP, GTP, UTP and ChromaTide Alexa Fluor 488-5-UTP (Invitrogen, C11403) solution (4:4:4:1:4), in which 80% of uracil was labeled by Alexa Fluor 488. The labeled RNA was then fragmented by adding 1× Ambion RNA fragmentation reagent (Ambion, AM8740) with incubation at 70 °C for 2 min. After adding the stop solution, the labeled RNA was purified and used as probes for DNA-FISH.

The zona pellucida of the mouse embryos was removed with incubation in acidic Tyrode’s solution. Then the embryos were incubated in PBSA for 3 min and transferred onto Superfrost/Plus microscope slides and dried as quickly as possible (less than 5 min). Embryos were permeabilized in 1% Triton X-100 in 1× PBS and fixed in ice-cold 100% methanol for 30 min at −20 °C. Then, slides were transferred into 70% ethanol on ice for 20 min. To perform RNA-FISH, the procedures for dehydration and hybridization were performed as described in the ‘IF combined with the RNA-FISH’ section. After three washes for 5 min each in hybridization washing solution at 42 °C and four washes for 5 min each in 2× SSC, slides were post-fixed in 3% paraformaldehyde (PFA; Sigma, 158127) in PBS for 10 min at room temperature.

To perform DNA-FISH, the embryos were incubated with RNase Cocktail (Invitrogen, AM2288) in 1× PBS for 1 h at 37 °C. After three washes with 1× PBS, embryos were permeabilized in permeabilization solution II (0.7% Triton X-100 and 0.1 M HCl in 1× PBS) for 15 min on ice. Then, slides were transferred into 70% ethanol on ice for 20 min. Dehydration was performed in 80%, 95% and 100% ethanol (×2), with each incubation lasting for 5 min at room temperature. Slides were then dried for 5 min. Then the slides were denatured in the hybridization washing solution for 30 min at 80 °C. After dehydration in cold ethanol, the embryos were hybridized in the hybridization solution containing 5 μg of Alexa Fluor 546-labeled DNA probes per slide at 37 °C overnight (14–15 h). After three washes for 5 min each in hybridization washing solution (50% Formamide, 2× SSC) at 42 °C and four washes for 5 min each in 2× SSC, embryos were mounted with DAPI-Vectashield solution (Vector laboratories, H1200). Fluorescence staining was imaged using a laser-scanning confocal microscope (Leica, TCS SP8). IMARIS software (Bitplane) was then used to calculate the number of speckles for each picture.

### Immunofluorescence combined with RNA-FISH and DNA-FISH

Immunofluorescence, RNA-FISH and DNA-FISH were performed as described above. For analysis of the heterogenous occupancy of *LincGET* speckles and PCBP1 around the *Carm1* gene loci, the embryos were injected with Alexa Fluor 488-labeled *LincGET*, pre-mRNAs for PCBP1-BFP and Alexa Fluor 546-labeled sgRNAs; then, the embryos were directly stained with SYTOX Deep Red Nucleic Acid Stain (Invitrogen, S11380) and Alexa Fluor 647 Phalloidin (Invitrogen, A22287) and then mounted after permeabilization.

### ESS percentage analysis from published data

The raw data were downloaded from the GEO database. To trim the original data, the trim_galore (v.0.6.7) software was used with the default parameters. Next, STAR software was used to align reads to mouse genome sequences (https://ftp.ebi.ac.uk/pub/databases/gencode/Gencode_mouse/release_M29/GRCm39.primary_assembly.genome.fa.gz) with default parameters. RMATS (v.4.1.0) software was used to analyze the alternative splicing events and Rmats2sashimiplot (v.2.0.4) software was used for visualization. The levels of all types of alternative splicing events on *Carm1* pre-mRNAs were analyzed, including exon-skipping, retained introns, alternative 5′-splicing site, alternative 3′-splicing site, mutually exclusive exons, alternative first exons and alternative last exons.

The percentage of ESS events on exons 3 to 6 relative to noS splicing events is calculated based on Psi values (Ψ). The ESS events on exons 3 to 6 conclude E3S, E5S, E6S, E56S and E3456S; thus, the calculation formulas are as follows:

$$P\rm{({noS}})=\frac{\frac{\it{R}\rm{({noS})}}{\it{L}\rm{(E2\;{to}\,E7)}}}{\frac{\it{R}\rm{({noS})}}{\it{L}\rm{(E2\;{to}\,E7)}}+\frac{\it{R}\rm{(E3S)}}{\it{L}\rm{(E2+E4)}}+\frac{\it{R}\rm{(E5S)}}{\it{L}\rm{(E4+E6)}}+\frac{\it{R}\rm{(E6S)}}{\it{L}\rm{(E5+E7)}}+\frac{\it{R}\rm{(E56S)}}{\it{L}\rm{(E4+E7)}}+\frac{\it{R}\rm{(E3456S)}}{\it{L}\rm{(E2+E7)}}}$$

$$P\rm{(E3S)}=\frac{\frac{\it{R}\rm{(E3S)}}{\it{L}\rm{(E2+E4)}}}{\frac{\it{R}\rm{({noS})}}{\it{L}\rm{(E2\;{to}\,E7)}}+\frac{\it{R}\rm{(E3S)}}{\it{L}\rm{(E2+E4)}}+\frac{\it{R}\rm{(E5S)}}{\it{L}\rm{(E4+E6)}}+\frac{\it{R}\rm{(E6S)}}{\it{L}\rm{(E5+E7)}}+\frac{\it{R}\rm{(E56S)}}{\it{L}\rm{(E4+E7)}}+\frac{\it{R}\rm{(E3456S)}}{\it{L}\rm{(E2+E7)}}}$$

$$P\rm{(E5S)}=\frac{\frac{\it{R}\rm{(E5S)}}{\it{L}\rm{(E4+E6)}}}{\frac{\it{R}\rm{({noS})}}{\it{L}\rm{(E2\;{to}\,E7)}}+\frac{\it{R}\rm{(E3S)}}{\it{L}\rm{(E2+E4)}}+\frac{\it{R}\rm{(E5S)}}{\it{L}\rm{(E4+E6)}}+\frac{\it{R}\rm{(E6S)}}{\it{L}\rm{(E5+E7)}}+\frac{\it{R}\rm{(E56S)}}{\it{L}\rm{(E4+E7)}}+\frac{\it{R}\rm{(E3456S)}}{\it{L}\rm{(E2+E7)}}}$$

$$P\rm{(E6S)}=\frac{\frac{\it{R}\rm{(E6S)}}{\it{L}\rm{(E5+E7)}}}{\frac{\it{R}\rm{({noS})}}{\it{L}\rm{(E2{\;to}\,E7)}}+\frac{\it{R}\rm{(E3S)}}{\it{L}\rm{(E2+E4)}}+\frac{\it{R}\rm{(E5S)}}{\it{L}\rm{(E4+E6)}}+\frac{\it{R}\rm{(E6S)}}{\it{L}\rm{(E5+E7)}}+\frac{\it{R}\rm{(E56S)}}{\it{L}\rm{(E4+E7)}}+\frac{\it{R}\rm{(E3456S)}}{\it{L}\rm{(E2+E7)}}}$$

$$P\rm{(E56S)}=\frac{\frac{\it{R}\rm{(E56S)}}{\it{L}\rm{(E4+E7)}}}{\frac{\it{R}\rm{({noS})}}{\it{L}\rm{(E2{\;to}\,E7)}}+\frac{\it{R}\rm{(E3S)}}{\it{L}\rm{(E2+E4)}}+\frac{\it{R}\rm{(E5S)}}{\it{L}\rm{(E4+E6)}}+\frac{\it{R}\rm{(E6S)}}{\it{L}\rm{(E5+E7)}}+\frac{\it{R}\rm{(E56S)}}{\it{L}\rm{(E4+E7)}}+\frac{\it{R}\rm{(E3456S)}}{\it{L}\rm{(E2+E7)}}}$$

$$P\rm{(E3456S)}=\frac{\frac{\it{R}\rm{(E3456S)}}{\it{L}\rm{(E2+E7)}}}{\frac{\it{R}\rm{({noS})}}{\it{L}\rm{(E2{to}\,E7)}}+\frac{\it{R}\rm{(E3S)}}{\it{L}\rm{(E2+E4)}}+\frac{\it{R}\rm{(E5S)}}{\it{L}\rm{(E4+E6)}}+\frac{\it{R}\rm{(E6S)}}{\it{L}\rm{(E5+E7)}}+\frac{\it{R}\rm{(E56S)}}{\it{L}\rm{(E4+E7)}}+\frac{\it{R}\rm{(E3456S)}}{\it{L}\rm{(E2+E7)}}}$$

where *P is* the percentage of the splicing event; *R* is the number of reads with the splicing event; and *L* is the length of exons.

For other alternative splicing events, including the alternative 3′-splicing site of exon 4 (E4A3SS), retained intron 14 (RI14), retained intron 15 (RI15), exon 2 skipping splicing (E2S), exon 3a inclusive (E3a), E11S and E15S, the percentage of these alternative splicing (AS) relative to non-alternative splicing (noAS) is calculated based on Ψ, and the formulas are as follows:

$$P\rm{({noAS}})=\frac{\it{R}\rm({noAS})}{\it{R}\rm{({noAS})}+\it{R}\rm{({AS})}}$$

$$P(\rm{{AS}})=\frac{\it{R}\rm{({AS})}}{\it{R}\rm{({noAS})}+\it{R}\rm{({AS})}}$$

where *P* is the percentage of the splicing event and *R* is the number of reads with the splicing event.

### SPAR-seq and data analysis

The zona pellucida of embryos was removed using Tyrode’s solution (Sigma, T1788). Zona-free embryos were incubated for 5 min in Ca^2+^-free and Mg^2+^-free M2 medium before disaggregation by careful pipetting. For single-cell SPAR-seq, each blastomere of two-cell or four-cell embryos was transferred to individual tubes containing 2.3 μl of lysis buffer (0.2% Triton X-100 supplemented with 1 U ml^−1^ ribonuclease inhibitor (Invitrogen, 10777019)). For bulk SPAR-seq, blastomeres of 50 embryos at the four-cell stage of each group were placed into two individual tubes containing 2.3 μl of lysis buffer separated by GFP^+^ or GFP^−^. Reverse transcription was performed with a Single Cell-to-CT qRT–PCR Kit (Invitrogen, 4458237). PCR was performed using primers targeting exon 2 and exon 7 of *Carm1* mRNA (Supplementary Table 3).

The samples were sequenced using an Illumina MiSeq sequencer with 300 bp paired-end sequencing reactions (PE300) at BGI company (https://www.genomics.cn). Clean reads were merged using pear (v.0.9.6)^55^ software with default parameters. Given that the noS and E3a (with longer exon 4, from XM_030244479.2) sequences are longer than 600 bp, the noS and E3a reads were separated into unassembled.forward.fastq and fq.unassembled.reverse.fastq files, whereas the AS reads (E3S, E4S, E5S, E6S, E34S, E56S, E345S and E3456S) and the *Gfp* spike-ins were separated into assembled.fastq files. To count the AS reads, reads in the assembled.fastq files were mapped to the reference transcripts, including all the exon-skipping candidates, with bowtie2 (v.2.2.5) software^56^ and transferred into *bam* files with samtools (v.1.9)^57^ software. To count the noS and E3a reads, Carm1_E3a_analysis.py (https://github.com/NEAU-Wang-lab/SPAR-seq) was used to note reads in unassembled.forward.fastq and fq.unassembled.reverse.fastq files.

Some samples were sequenced on a PacBio Sequel System according to the standard PacBio Iso-Seq procedures at Annoroad company (http://www.annoroad.com) mixed with samples from other experiments. Then, circular consensus sequencing (CCS) reads were generated using ccs (v.5.0.0) software and converted to fastq format using bam2fastq software in the pbbam (v.1.0.6) package. The number of passes for each of the raw CCS reads was generated using GetCCSpass.pl (https://github.com/Lulab-IGDB/polyA_analysis/blob/main/bin). Then the clean reads of *Carm1* were extracted from the CCS reads using Carm1_SPAR_reads_extract.py. Carm1_AS_analysis.py (https://github.com/NEAU-Wang-lab/SPAR-seq) was used to count the AS reads, and Carm1_E3a_analysis.py was used to count the noS and E3a reads.

### SHAPE-MaP assays and data analysis

The in vitro transcribed *LincGET* was re-folded in folding buffer (100 mM NaCl, 100 mM HEPES pH 8.0 and 10 mM MgCl_2_ in water) at 37 °C for 20 min with or without NONO, PSPC1 or HNRNPU. As a control, one group of *LincGET* was denatured in denaturing control buffer (50% formamide, 50 mM HEPES pH 8.0 and 4 mM EDTA pH 8.0 in water) at 95 °C for 1 min. For each group, approximately 5 µg RNA was added to the one-ninth volume of NMIA (Invitrogen, M25) at 100 mM in neat DMSO (10 mM final concentration) and incubated at 37 °C for 22 min. The background was assessed by performing no-reagent and denaturing controls.

After fragmentation with RNA fragmentation reagent (Ambion, AM8740), modified *LincGET* was subjected to MaP reverse transcription^27^, with SuperScript II Reverse Transcriptase (Invitrogen, 18064014) under Mn^2+^ conditions (50 mM Tris-HCl pH 8.0, 75 mM KCl, 10 mM DTT, 2 mM dNTPs and 15 mM MnCl_2_ in water) using random nonamer primers (200 ng μl^−1^; NEB, S1254S). After synthesizing the second strand by NEBNext pre-mRNAs Second Strand Synthesis Module (NEB, E6111S), the resulting cDNAs were constructed for high-throughput sequencing libraries and sequenced by BGI company.

The deep sequencing datasets were analyzed by ShapeMapper2 (v.2.1.5)^58^ software with default parameters. The reads were mapped to target sequences by bowtie2 (v.2.2.5)^56^ software with default parameters as recommended by ShapeMapper2. RNA secondary structures were modeled by Superfold (v.1.0)^59^ with map files produced by ShapeMapper2. The RNA stem-loop structures for specific fragments were produced by VARNA (v.3.93)^60^. The ct files from Superfold were used for visualization and varna_colors.txt files from ShapeMapper2 output were used for the reactivity coloring.

### Protein purification

Plasmids expressing the NONO, PCBP1 or PCBP2 fused to EGFP tagged with MBP and 6×His (MBP–NONO-EGFP–6×His) were transformed into Transetta (DE3) Chemically Competent Cells (TransGen, CD801). A fresh bacterial colony was inoculated into 4 ml LB media containing ampicillin and grown for 6–8 h at 37 °C until the A600 reached about 0.5. Cells were diluted into 600 ml (1:150) room temperature LB with freshly added ampicillin and grown for 8–12 h at 37 °C. IPTG (TransGen, GF101-01) was added to 1 mM and growth continued for 18 h at 16 °C. Pellets from 600 ml cells were resuspended in 30 ml of Buffer A (50 mM Tris pH 7.5, 500 mM NaCl, 1% Triton X-100, 10 mM imidazole and 1× cOmplete protease inhibitors (Roche, 11873580001)) and divided equally into three 50 ml tubes. For each tube containing 10 ml suspended cells, 15 min of sonication (90 cycles of 5 s on, 5 s off) at 300 W was suitable. The lysate was mixed and cleared by centrifugation at 8,000*g* for 5 min at 4 °C followed by filtration with a 0.45 μm filter.

Next, the MBP-tagged proteins were purified using a PurKine MBP-Tag Dextrin Packed Column (Dextrin, BMC20206), according to the manufacturer’s instructions. In brief, the top and bottom stoppers on the column were removed to let the stored buffer drain away. Two aliquots of resin-bed volume binding/washing buffer (2 mM Tris-HCl, 20 mM NaCl, 0.1 mM EDTA pH 8.0 and 1 mM DTT) were added to the column and drained away to equilibrate the column. The equilibration step was repeated three times. The cleared lysate was mixed with equal binding/washing buffer and added to the column. For maximal binding, the sample was incubated for 30 min at room temperature or 4 °C. After the sample was drained away, two aliquots of resin-bed volume binding/washing buffer were added to the column and drained to remove the non-specifically adsorbed protein. The washing step was repeated six times. Then, 10 ml of elution buffer (2 mM Tris-HCl, 0.1 mM EDTA pH 8.0, 1 mM Maltose pH 7.4 and 1 mM DTT) was added to the column to wash the target protein. The wash liquid was collected and the content was analyzed using a Coomassie-stained SDS–PAGE gel.

After testing the protein concentration using the Super-Bradford Protein Assay Kit (CWBio, CW0013S), the MBP tag was removed from the purified protein using Factor Xa Protease (NEB, P8010L), in which 1 μg of Factor Xa was added to 50 μg of MBP fusion protein for 24 h at 4 °C.

Next, the sample containing NONO-EGFP–6×His, PCBP1-EGFP–6×His or PCBP2-EGFP–6×His was purified by ProteinIso Ni-NTA Resin (TransGen, DP101-02) combined with an Affinity Chromatography Column (12 ml; TransGen, GP101-03), according to the manufacturer’s instructions. In brief, 6 ml Ni-NTA was added to the column and equilibrated with 60 ml Buffer A. The sample was poured into the column and then washed with 15 volumes of Buffer B (20 mM Tris pH 7.5, 500 mM NaCl, 1% NP-40, 1 mM DTT, 10 mM imidazole and 1× cOmplete protease inhibitors). Protein was eluted with 4 ml Buffer C (20 mM Tris pH 7.5, 500 mM NaCl, 1% NP-40, 1 mM DTT, 200 mM imidazole, and 1× cOmplete protease inhibitors). The wash liquid was collected and the contents were analyzed using a Coomassie-stained SDS–PAGE gel.

Next, the sample (4 ml) was dialyzed in Slide-A-Lyzer 20K Dialysis Cassettes (Thermo, 66012) against 1 l of Buffer D (50 mM Tris-HCl pH 7.5, 125 mM NaCl, 10% glycerol, 1 mM DTT) twice at 4 °C for 8 h each time. Then the sample was concentrated using 30K MWCO Amicon ΜLtra Centrifugal Filters (Millipore, UFC803024) to 200–1,000 μl via centrifuge at 7,500×*g* at 4 °C for 30 min. The protein concentration was measured using the Super-Bradford Protein Assay Kit (CWBio, CW0013S).

### ChIP and data analysis

For chromatin immunoprecipitation to analyze *LincGET* binding DNA sites, embryos were injected with the *LincGET-MS2* and mRNAs for HA-tagged MS2P at the pronuclear stage and collected at the early four-cell stage. For each batch, embryos were washed three times with 1× PBS and were crosslinked for 40 min in a droop containing 1.5 mM freshly prepared ethylene glycol bis(succinimidyl succinate) (EGS; Thermo Scientific, 21566) in a chemical fume hood at room temperature with rotation. Then the embryos were dual-crosslinked in a droop containing 1% formaldehyde (Sigma, 8187081000) for 20 min in a chemical fume hood at room temperature with rotation. To quench the crosslinkers, the embryos were moved to a droop containing 200 mM glycine and were incubated for 10 min at room temperature with rotation in a chemical fume hood. Then embryos were washed three times with 1× PBS and stored at −80 °C with as little liquid as possible until use.

Some batches were combined to collect about 1,000 embryos for each replicate using 20 μl nuclei extraction buffer (10 mM Tris-HCl pH 8.5 (Sigma, 87772), 140 mM NaCl (Sigma, S6546), 5 mM MgCl_2_ (Invitrogen, AM9530G), 0.6% NP-40 (Abcam, ab142227), 1 mM PMSF, 1× protease unhibitors complex (PIC, CST, 7012)) and incubated for 2 min on ice. For chromatin digestion, the samples were added with 80 μl chromatin digestion buffer (1× MNase buffer (NEB, M0247S), 2 mM DTT, 5% PEG6000 (Avantor, 1008060), 60 U μl^−1^ MNase (NEB, M0247S)) and incubated at room temperature for 5 min. Then the samples were added with 11 μl MNase stop solution and incubated at room temperature for 1 min. Next, the samples were added with 13 μl nuclear break buffer (1% Triton X-100, 1% odium deoxycholate (Sigma, 30970), 1× PIC) and 86 μl 10× ChIP buffer (CST, 7008) to a final volume of 210 μl, 10 μl of which was treated as input and the other 200 μl of which was used for immunoprecipitation. Before immunoprecipitation, the magnetic beads (CST, 9006) were washed three times with 1× ChIP buffer.

For immunoprecipitation, each sample was added with 1 μg anti-HA antibody (mass:volume ratio, 1:200) and incubated overnight at 4 °C with rotation. Then the samples were added with 12 μl magnetic beads (CST, 9006) and incubated for 5 h at 4 °C with rotation. Next, the beads were washed with 1× ChIP buffer four times for 5 min each time at 4 °C with rotation and with high salt wash buffer (300 μl 10× ChIP buffer, 2,700 μl H_2_O, 210 μl 5 M NaCl) three times for 5 min at 4 °C with rotation. After washing, chromatin was eluted from the beads with 100 μl 1× ChIP elution buffer (CST, 7009) with incubation at 65 °C for 30 min. The reverse crosslink was performed by adding 4 μl 5 M NaCl and 1 μl proteinase K (20 mg ml^−1^; Beyotime, ST533) and incubation at 65 °C for 4 h. Then the DNA was extracted by 100 μl chloroform and was precipitated by adding 10 μl of 3 M sodium acetate pH 5.5 (Invitrogen, AM9740), 300 μl isopropanol and 1 μl glycogen (15 mg ml^−1^; Invitrogen, AM9516) and incubation for 30 min at −80 °C. After washing twice with cold 75% ethanol, the DNA pellet was dissolved with 10 μl of nuclease-free water. Finally, DNA library preparation and sequencing on Hiseq 2500 were performed by BGI company.

For H3R26me2 ChIP–qPCR, anti-H3R26me2 antibody (Abcam, ab127095) was used for immunoprecipitation (mass:volume ratio, 1:200), and qPCR was performed instead of DNA library preparation and sequencing.

The deep sequencing datasets were trimmed by trim_galore (v.0.6.7)^61^ software with default parameters. The reads were mapped to mouse genome sequences (https://ftp.ebi.ac.uk/pub/databases/gencode/Gencode_mouse/release_M29/GRCm39.primary_assembly.genome.fa.gz) by bwa (v.0.7.17-r1188)^62^ software with default parameters and transferred into bam files with samtools (v.1.9)^57^ software. Finally, the bam files were loaded into the IGV (v.2.16.2) for visualization. Peak calling was performed by macs2 (v.2.1.1.20160309) software after duplex removing by picard (v.2.18.29-0) software.

### RNA electrophoretic mobility shift assays

RNA oligonucleotides were in vitro transcribed using HiScribe T7 Quick High Yield RNA Synthesis Kit (NEB, E2050) following the manufacturers’ guidelines. DNA templates with a T7 promoter were generated by annealing with primers listed in Supplementary Table 3. RNA oligonucleotides were then biotinylated using the Pierce RNA 3′ End Desthiobiotinylation Kit (Thermo Scientific, 20163) according to the manufacturer’s instructions. The labeled oligonucleotides were gel-purified on 12% denaturing gels before use. The gel shift assay was carried out using the LightShift Chemiluminescent RNA EMSA Kit (Thermo Scientific, 20158). In brief, 5 ng biotinylated wild-type or mutant RNA probe was mixed with 50 μg of 6×His-purified PCBP1 and PCBP2 mix (6×His–PCBP1) (and anti-PCBP1 or anti-PCBP2 antibody for super-shifts (mass:volume ratio, 1:500)) and incubated at room temperature for 30 min in a 20 μl binding reaction containing 1× binding buffer, 5% glycerol and 0.1 mg ml^−1^ tRNA. The samples were electrophoresed on a 5% native PAGE in 0.5× Tris Borate EDTA (Thermo Scientific, B52), transferred to a positively charged BrightStar-Plus Nylon membrane (Invitrogen, AM10100) and crosslinked in a UV Stratalinker 1800 (Stratagene). To block the membrane, the membrane was incubated in 20 ml nucleic acid detection blocking buffer for 15 min with gentle shaking and then was incubated in 20 ml conjugate/blocking solution (20 ml nucleic acid detection blocking buffer containing 66.7 μl stabilized streptavidin-horseradish peroxidase conjugate) for 15 min with gentle shaking. After washing four times for 5 min each in 20 ml 1× wash buffer with gentle shaking, the membrane was incubated in 30 ml substrate equilibration buffer for 5 min with gentle shaking. Then the membrane was incubated in 12 ml substrate working solution (6 ml luminol/enhancer solution and 6 ml stable peroxide solution) for 5 min without shaking. Finally, the membrane was exposed to a Bio-Rad GelDoc XR+ Gel imaging system for the acquisition of signals.

### Minigene analysis of ESS

The minigene plasmids and plasmids encoding shRNAs targeting *Pcbp1/2* or control shRNAs were co-transfected into MCF-7 cells. Cells were collected 72 h post transcription, and total RNA was extracted. Then RT–PCR was performed with the primers listed in Supplementary Table 3.

### Dot blotting

Single blastomeres were separated from late two-cell and early four-cell embryos and transferred into PCR tubes containing 10 μl of 0.2% (vol/vol) Triton X-100 (Sigma, T9284) and 0.5 μl of RNase inhibitor (Clontech, 2313A). The lysate was split into three equal portions, which were used for dot plotting for CARM1, qPCR for the ESS isoform *Carm1_E56S* and qPCR for the total expression level of *Carm1*. For dot blotting, lysates from a series of single blastomeres were spotted onto a nitrocellulose membrane and allowed to absorb. Then the membrane was blocked, incubated sequentially with anti-CARM1 antibody (mass:volume ratio, 1:100) and fluorescence-labeled secondary antibody (mass:volume ratio, 1:5,000), and then exposed to a Bio-Rad GelDoc XR+ Gel imaging system in the same way as those in the 'Western blot' section above.

### Secondary structure comparison between *LincGET* and *Neat1*

The RNA secondary structures of *LincGET* and *Neat1* were predicted and compared using the ViennaRNA package^63^. Firstly, sequences of *LincGET* and *Neat1* were split into 500 nt fragments, with the two adjacent fragments overlapped by 100 nt. Then the RNAfold function was used to predict the RNA secondary structures and the RNAdistance function was used to measure the dissimilarity of the RNA secondary structures in a bracket format. The resulting *f* values are shown in Supplementary Table 5. The smaller the *f* value, the more similar the structure.

To determine the *f* values that represent high enough structural similarity, the distribution of *f* values from the comparison between 52 *Neat1* fragments and 10,000 random mRNA fragments (500 nt each) was plotted. We found that the distribution curve is approximately normal, and the boundary of the left 2.5% (*P* < 0.05) is 320 (Supplementary Fig. 2c). Therefore, we considered structural similarity to be high enough if the *f* value is less than 320.

Two comparisons with *f* values less than 320 (Supplementary Fig. 2d and Supplementary Table 5) were found. The secondary structures of these fragments were predicted using the RNAfold web server (http://rna.tbi.univie.ac.at/cgi-bin/RNAWebSuite/RNAfold.cgi).

### Absolute qPCR for *LincGET* and *Neat1_2*

Firstly, PCR using Q5U Hot Start High-Fidelity DNA Polymerase (NEB, M0515) with primers for TaqMan quantitative real-time PCR (Supplementary Table 3) was performed, and the PCR products were purified using the Zymoclean Gel DNA Recovery Kit (ZYMO, D4007). Standard curves were made using the purified PCR results for *LincGET* and *Neat1*. Serial diluted PCR products were used. Standard curves with threshold cycle (*C*_t_) values on the *x* axis and the logarithmic value with base 2 (log_2_) of molecular concentration (number of molecules/μl) on the *y* axis were generated for quantification (Supplementary Table 6). Next, three groups of late two-cell embryos (200 embryos each), three groups of early four-cell embryos (200 embryos each) and three groups of mEpiSCs using FACS (10,000 cells each) were collected. After total RNA extraction and reverse transcription, real-time PCR was performed using TaqMan Universal Master Mix II. The copy numbers of *LincGET* and *Neat1* in late two-cell and early four-cell embryos as well as in mEpiSCs were calculated in Excel.

### Statistical analyses

Statistical analyses (mean ± s.e.m.) for differential gene expression, differential fluorescence intensity and differential abundance on gels were performed in Excel. Levels of significance were calculated with Student’s *t*-tests. Isoform abundance on SDS–PAGE gels or agarose gels was measured in Fiji/ImageJ. The co-localization analysis of *LincGET* signals with NONO, PSPC1, PCBP1, U2AF2, SRSF1 or *Carm1* gene locus in live imaging assays, RNA-FISH combined with immunofluorescence assays and RNA-FISH combined with DNA-FISH assays were calculated by the two (green and red) or three (green, red and blue) separate channel signals per nucleus using Pearson’s correlation coefficient with Coloc 2 plugins in Fiji/ImageJ. Line scans of the relative fluorescence intensity of signals were drawn by separate channel signals with the Plot Profile plugins in Fiji/ImageJ. Particle numbers of *LincGET* speckles, NONO speckles, PSPC1 speckles and PCBP1 speckles were calculated by Imaris with the particle building system. The *R*^2^ value for linear regression was calculated in Excel.

### Reporting summary

Further information on research design is available in the Nature Portfolio Reporting Summary linked to this article.

## Online content

Any methods, additional references, Nature Portfolio reporting summaries, source data, extended data, supplementary information, acknowledgements, peer review information; details of author contributions and competing interests; and statements of data and code availability are available at 10.1038/s41594-024-01292-9.

## Supplementary information

Supplementary Information Supplementary Discussion, and Supplementary Figs. 1–5.

Reporting Summary

Supplementary Table 1 Sheet ‘S Table 1’: Supplementary Table 1. The published data from others used in Fig. 1a,b, Extended Data Fig. 1, Supplementary Fig. 1. Sheet ‘S Table 2’: Supplementary Table 2. Data analysis of PacBio long-read SPAR-seq. Sheet ‘S Table 3’: Supplementary Table 3. Oligos used in the paper. Sheet ‘S Table 4’: Supplementary Table 4. The sequence complementarity of *LincGET* to the *Carm1* gene locus analyzed by *LongTarget*. Sheet ‘S Table 5’: Supplementary Table 5. *f* values to measure the dissimilarity of the RNA secondary structures of fragments of *LincGET* and *Neat1*. Sheet ‘S Table 6’: Supplementary Table 6. Absolute quantification for *LincGET* and *Neat1_2* in late two-cell and early four-cell embryos as well as in mEpiSCs. Sheet ‘S Table 7’: Supplementary Table 7. Occupancy of *LincGET*-speckles and PCBP1 around the *Carm1* gene loci in blastomeres of late two-cell and early four-cell embryos. Sheet ‘S Table 8’: Supplementary Table 8. Analysis of the distribution of the progeny of injected blastomere at the blastocyst stage.

Source Data Supplementary Fig. 1 Statistical Source data for bar plots in Supplementary Fig. 1.

Source Data Supplementary Fig. 2 Statistical Source data for bar plots in Supplementary Fig. 2c

Source Data Supplementary Fig. 3 Sheet ‘S Fig. 3a’: Statistical Source data for line scans and Rp values in Supplementary Fig. 3a. Sheet ‘S Fig. 3b’: Statistical Source data for Rp values in Supplementary Fig. 3b. Sheet ‘S Fig. 3c’: Statistical Source data for line scans and Rp values in Supplementary Fig. 3c. Sheet ‘S Fig. 3e’: Statistical Source data for line scans and Rp values in Supplementary Fig. 3e. Sheet ‘S Fig. 3f’: Statistical Source data for Rp values in Supplementary Fig. 3f.

Unmodified Gels Supplementary Fig. 3 Unprocessed western Blots for Supplementary Fig. 3d.

## Source data

Source Data Fig. 1 Sheet ‘Fig. 1b’: Statistical Source data for bar plot in Fig. 1b. Sheet ‘Fig. 1c’: Statistical Source data for bar plot and dot plots in Fig. 1c.

Source Data Fig. 2 Sheet ‘Fig. 2a’: Statistical Source data for bar plots and dot plots in Fig. 2a. Sheet ‘Fig. 2c’: Statistical Source data for line scans in Fig. 2c. Sheet ‘Fig. 2e’: Statistical Source data for line scans, Rp values, and dot plots in Fig. 2e. Sheet ‘Fig. 2f’: Statistical Source data for line scans and Rp values in Fig. 2f. Sheet ‘Fig. 2g’: Statistical Source data for bar plot and dot plot in Fig. 2g.

Source Data Fig. 2 Unprocessed Gels and Western Blots for Fig. 2b,d,g.

Source Data Fig. 3 Sheet ‘Fig. 3a’: Statistical Source data for line scans in Fig. 3a. Sheet ‘Fig. 3b’: Statistical Source data for pie chart in Fig. 3b. Sheet ‘Fig. 3d’: Statistical Source data for line scans and Rp values in Fig. 3d. Sheet ‘Fig. 3f’: Statistical Source data for line scans in Fig. 3f. Sheet ‘Fig. 3g’: Statistical Source data for line scans and Rp values in Fig. 3g. Sheet ‘Fig. 3h’: Statistical Source data for line scans and Rp values in Fig. 3h. Sheet ‘Fig. 3i’: Statistical Source data for bar plots and dot plots in Fig. 3i.

Source Data Fig. 3 Unprocessed Gels and Western Blots for Fig. 3e.

Source Data Fig. 4 Sheet ‘Fig. 4c’: Statistical Source data for bar plots and dot plots in Fig. 4c. Sheet ‘Fig. 4d’: Statistical Source data for line scans, Rp values, and dot plots in Fig. 4d. Sheet ‘Fig. 4e’: Statistical Source data for bar plots and dot plots in Fig. 4e. Sheet ‘Fig. 4f’: Statistical Source data for line scans and Rp values in Fig. 4f.

Source Data Fig. 4 Unprocessed REMSA Blots for Fig. 4b.

Source Data Fig. 5 Sheet ‘Fig. 5b’: Statistical Source data for histograms in Fig. 5b. Sheet ‘Fig. 5c’: Statistical Source data for dot plots in Fig. 5c. Sheet ‘Fig. 5e’: Statistical Source data for dot plots in Fig. 5e. Sheet ‘Fig. 5g’: Statistical Source data for bar plots and dot plots in Fig. 5g.

Source Data Fig. 5 Unprocessed Western Blots for Fig. 5g.

Source Data Extended Data Fig. 2 Sheet ‘ED Fig. 2a’: Statistical Source data for bar plots in Extended Data Fig. 2a. Sheet ‘ED Fig. 2b’: Statistical Source data for bar plots and dot plots in Extended Data Fig. 2b. Sheet ‘ED Fig. 2c’: Statistical Source data for dot plots in Extended Data Fig. 2c. Sheet ‘ED Fig. 2d’: Statistical Source data for dot plots in Extended Data Fig. 2d. Sheet ‘ED Fig. 2e’: Statistical Source data for bar plots and dot plots in Extended Data Fig. 2e.

Source Data Extended Data Fig. 2 Unprocessed Gels and Western Blots for Extended Data Fig. 2b,e.

Source Data Extended Data Fig. 3 Sheet ‘ED Fig. 3a’: Statistical Source data for bar plots and dot plots in Extended Data Fig. 3a. Sheet ‘ED Fig. 3b’: Statistical Source data for bar plots and dot plots in Extended Data Fig. 3b. Sheet ‘ED Fig. 3d’: Statistical Source data for line scans, Rp values, and dot plots in Extended Data Fig. 3d.

Source Data Extended Data Fig. 3 Unprocessed Gels for Extended Data Fig. 3b.

Source Data Extended Data Fig. 4 Sheet ‘ED Fig. 4a’: Statistical Source data for line scans, Rp values, and dot plots in Extended Data Fig. 4a. Sheet ‘ED Fig. 4b’: Statistical Source data for bar plots and dot plots in Extended Data Fig. 4b.

Source Data Extended Data Fig. 4 Unprocessed Western Blots for Extended Data Fig. 4b.

Source Data Extended Data Fig. 5 Sheet ‘ED Fig. 5a’: Statistical Source data for bar plots and dot plots in Extended Data Fig. 5a. Sheet ‘ED Fig. 5c’: Statistical Source data for bar plots and dot plots in Extended Data Fig. 5c. Sheet ‘ED Fig. 5e’: Statistical Source data for bar plots and dot plots in Extended Data Fig. 5e.

Source Data Extended Data Fig. 5 Unprocessed Gels for Extended Data Fig. 5e.

Source Data Extended Data Fig. 6 Sheet ‘ED Fig. 6a’: Statistical Source data for line scans and Rp values in Extended Data Fig. 6a. Sheet ‘ED Fig. 6b’: Statistical Source data for line scans in Extended Data Fig. 6b. Sheet ‘ED Fig. 6c’: Statistical Source data for line scans and Rp values in Extended Data Fig. 6c.

Source Data Extended Data Fig. 7 Sheet ‘ED Fig. 7b’: Statistical Source data for bar plots and dot plots in Extended Data Fig. 7b. Sheet ‘ED Fig. 7c’: Statistical Source data for bar plots and dot plots in Extended Data Fig. 7c. Sheet ‘ED Fig. 7d’: Statistical Source data for bar plots and dot plots in Extended Data Fig. 7d. Sheet ‘ED Fig. 7e’: Statistical Source data for bar plots and dot plots in Extended Data Fig. 7e.

Source Data Extended Data Fig. 7 Unprocessed Gels and western Blots for Extended Data Fig. 7b,c,e.

Source Data Extended Data Fig. 8 Sheet ‘ED Fig. 8b’: Statistical Source data for dot plots in Extended Data Fig. 8b. Sheet ‘ED Fig. 8c’: Statistical Source data for dot plots in Extended Data Fig. 8c.

Source Data Extended Data Fig. 9 Sheet ‘ED Fig. 9b’: Statistical Source data for bar plots and dot plots in Extended Data Fig. 9b. Sheet ‘ED Fig. 9c’: Statistical Source data for bar plots and dot plots in Extended Data Fig. 9c.

Source Data Extended Data Fig. 9 Unprocessed Gels for Extended Data Fig. 9b,c.

Source Data Extended Data Fig. 10 Sheet ‘ED Fig. 10a’: Statistical Source data for dot plots in Extended Data Fig. 10a. Sheet ‘ED Fig. 10b’: Statistical Source data for bar plots and dot plots in Extended Data Fig. 10b. Sheet ‘ED Fig. 10c’: Statistical Source data for dot plots and Rp values in Extended Data Fig. 10c.

Source Data Extended Data Fig. 10 Unprocessed western Blots and dot Blots for Extended Data Fig. 10b,c.

## Data Availability

The sequencing data of SPAR-seq, SHAPE-MaP-seq and *LincGET* ChIP–seq have been deposited in the Genome Sequence Archive of Beijing Institute of Genomics, Chinese Academy of Sciences (GSA; http://gsa.big.ac.cn) with accession numbers CRA007472, CRA007494 and CRA007495, respectively. This study also includes analysis of the following published data in the Gene Expression Omnibus database (GEO): GSE85019, GSE71257 (ref. ^64^), GSE163724 (ref. ^65^), GSE135457 (ref. ^66^), GSE45719 (ref. ^67^), GSE127106, GSE138760 (ref. ^23^), GSE153530 (ref. ^68^), GSE98150 (ref. ^69^), GSE66582 (ref. ^70^), GSE135678 (ref. ^71^), GSE151704, GSE171760 (ref. ^72^), GSE160894 (ref. ^73^), GSE169632 (ref. ^74^), GSE137630 (ref. ^75^), GSE161998 (ref. ^51^), GSE180259 (ref. ^76^), GSE165133 (ref. ^77^), GSE117815, GSE178298 (ref. ^78^), GSE162352 (ref. ^79^), GSE181800 (ref. ^52^), GSE173471 (ref. ^80^), GSE242289, GSE194115 (ref. ^53^), GSE194203, GSE201938, GSE226534 (ref. ^81^), GSE192404 (ref. ^54^), GSE151260 (ref. ^82^), GSE156568 (ref. ^83^), GSE228894 (ref. ^84^), GSE199546 (ref. ^85^), GSE189015, GSE150510 (ref. ^86^), GSE148019 (ref. ^87^), GSE184348 (ref. ^88^), GSE179888 (ref. ^89^), GSE181651, GSE197122 (ref. ^90^), GSE167360 (ref. ^91^), GSE234841 (ref. ^92^), GSE202260 (ref. ^93^), GSE190199, GSE114450 (ref. ^94^), GSE196236 (ref. ^35^), GSE147574, GSE235546, GSE71434 (ref. ^95^), GSE159484 and GSE149785 (ref. ^96^); and data from the GSA: CRA007513 (ref. ^2^). The mouse genome sequences are available at the website https://ftp.ebi.ac.uk/pub/databases/gencode/Gencode_mouse/release_M29/GRCm39.primary_assembly.genome.fa.gz. Source data are provided with this paper.

## Code availability

For analysis of SPAR-seq data, the Carm1_SPAR_reads_extract.py, Carm1_AS_analysis.py, and Carm1_E3a_analysis.py are available on the website https://github.com/NEAU-Wang-lab/SPAR-seq, and the GetCCSpass.pl is available on the website https://github.com/Lulab-IGDB/polyA_analysis/blob/main/bin/.
