# Supplementary material for: Alternative splicing of CARM1 regulated by LincGET-guided paraspeckles biases the first cell fate in mammalian early embryos
Source: Nat Struct Mol Biol. 2024 Apr 24;31(9):1341–54. doi: 10.1038/s41594-024-01292-9 (PMC11402786; doi:10.1038/s41594-024-01292-9)
Supplement: Supplementary file 1 — Supplementary Discussion, and Supplementary Figs. 1–5. [file 41594_2024_1292_MOESM1_ESM.pdf]

nature structural & molecular biology

Article

https://doi.org/10.1038/s41594-024-01292-9

Alternative splicing of CARM1 regulated by *LincGET*-guided paraspeckles biases the first cell fate in mammalian early embryos

In the format provided by the authors and unedited

Supplementary Discussion

Besides, we analyzed the SINE, LINE, and LTR elements in *Carm1* gene locus among mice, rats, rabbits, dogs, pigs, monkeys, and humans, and found these elements are conserved in *Carm1* gene locus among these mammalian species (Extended Data Fig. 15). It raised the possibility that the model in which CARM1 heterogeneity is partially controlled by retrotransposon-associated lincRNA is conserved in other mammals.

The localization of many subnuclear speckles is regulated by their lncRNA component[1]. Generally, paraspeckles in somatic cells are built around the lncRNA *Neat1*. *Neat1* is not the only lncRNA involved in paraspeckles, for example, another lncRNA termed CTN-RNA, localizes specifically to paraspeckles in a number of cell types[2]. Besides, in human pluripotent cells, *NEAT1* is poorly expressed and paraspeckles are barely detected[3]. This indicated that besides *Neat1*, paraspeckle components might assemble around other lncRNAs in different cell types for different functions. Interestingly, *Neat1* ablation in mouse two- to four-cell embryos resulted in re-localization of partial NONO from paraspeckles around the nucleoli[4] rather than disrupting paraspeckles completely, while *Neat1* ablation in HeLa and NIH-3T3 cells led to disappearance of paraspeckle that all NONO diffused throughout the nucleoplasm[5]. Besides, paraspeckles in somatic cells form near *Neat1* gene loci[6], but we here and others[4] found that paraspeckle components in mouse two- to four-cell embryos distribute evenly throughout the nucleoplasm. Our data here indicate that *LincGET* is the major lincRNA component involved in the assembly of paraspeckles in mouse two- to four-cell embryos and is essential for the localization of paraspeckles to the *Carm1* gene locus. *LincGET* depletion led to the disappearance of paraspeckles in mouse early embryos, which is similar to *Neat1* ablation for paraspeckles in HeLa and NIH-3T3 cells[5]. The involvement of the early embryo-specific *LincGET* in the assembly of paraspeckles in mouse two- to four-cell embryos reveals the highly specialized function and regulation of paraspeckles in early embryos, highlighting the cell-type specific function of paraspeckles through different lncRNA.

Our results indicated that PCBP1 accumulates in the nucleus of mouse late two- and early four-cell embryos (Extended Data Fig. 9a, b), which may be responsible for driving the change in alternative splicing under physiological conditions during late two- to early four-cell stage under the major wave of zygotic genome activation (ZGA). *LincGET*, which is specifically expressed in the nucleus of mouse late two- and early four-cell embryos, may work as a balancing mechanism to

prevent the alternative splicing from being excessive.

It is reported that a lot of splicing factors, such as SRAF3, undergo exon-skipping splicing in mouse early embryos, especially at the two-cell stage[7]. CARM1 is also reported as one client protein of paraspeckles that can influence the paraspeckle organization in two- to four-cell embryos[4]. Both elevating CARM1 levels and inhibiting CARM1 impair the organization of paraspeckles, which suggests that CARM1 might feedback and regulate its expression level via alternative splicing. It is consistent with the observation that there is a regulatory cascade of alternative splicing events where some key splicing regulators are controlled by alternative splicing of their gene transcripts[7]. This may be the reason why alternative splicing is extremely complicated and extraordinary during preimplantation development. This study reveals that the high exon-skipping splicing promoting activity can be partially counteracted by the *LincGET*-guided paraspeckles in a locus-specific manner, providing mechanistic insight into the negative regulation side of exon-skipping splicing in mouse early embryos. In addition, the mechanisms responsible for the strong promotion of exon-skipping splicing beyond *Pcbp1/2* in mouse early embryos is a fascinating question to be explored in the future.

We found that *Pcbp1/2* silencing doesn't significantly alter TE fate, which may be due to that depletion of *Pcbp1/2* didn't affect CARM1 protein level (Fig. 5g), even though *Pcbp1/2* depletion decreased the exon-skipping splicing of *Carm1* pre-mRNAs in mouse four-cell embryos (Fig. 4c, Extended Data Fig. 9e). PCBP1, plays important roles in both transcription regulation and translation control[8]. PCBP1 binds the 3'-untranslational region (3'-UTR) of $p27^{Kip1}$ mRNA, increases its stability, and enhances its translation[9]. The evidence indicates that PCBP1 not only can downregulate protein level through promoting exon-skipping splicing of its binding mRNAs, but also can upregulate protein level through enhance translation of its binding mRNAs.

LincGET-guided paraspeckles to the *Carm1* genome locus requires HNRNPU. The DNA binding and RNA binding ability of HNRNPU gives it an essential role in the high-order arrangement of the nucleus[10]. For example, HNRNPU is necessary for the correct localization of the X chromosome inactivation specific transcript *Xist* to establish gene silencing and form the highly structured chromatin territories[11-13]. Whether *LincGET* participates in high order nuclear organization is an interesting direction to explore in the future.

We found that the noS and E5S of *Carm1* pre-mRNAs are translated into protein, while the E3S, E6S, E56S, and E3456S cannot be translated into protein (Extended Data Fig. 3e), suggesting that

the exon-skipping splicing events do cause frame shift of the CARM1 protein during translation. It is known that the extra stop codon generated by frame shift will activate the non-sense mediated mRNA decay (NMD) pathway, a selective RNA turnover mechanism to control steady-state RNA levels[14]. Although the exon-skipping splicing events cause the decrease of CARM1 protein level, we found the mRNA level of CARM1 remained stable at two- to four-cell stage (Fig. 1c, Extended Data Fig. 3c, d), thus whether and how the NMD pathway is involved in balancing between RNA synthesis and RNA turnover to control RNA levels during early embryonic development is a field worthy of extensive study in the future work.

References

1. Batista, P.J. & Chang, H.Y. Long noncoding RNAs: cellular address codes in development and disease. *Cell* **152**, 1298-307 (2013).
2. Prasanth, K.V. et al. Regulating gene expression through RNA nuclear retention. *Cell* **123**, 249-63 (2005).
3. Chen, L.L. & Carmichael, G.G. Altered nuclear retention of mRNAs containing inverted repeats in human embryonic stem cells: functional role of a nuclear noncoding RNA. *Mol Cell* **35**, 467-78 (2009).
4. Hupalowska, A. et al. CARM1 and Paraspeckles Regulate Pre-implantation Mouse Embryo Development. *Cell* **175**, 1902-1916 e13 (2018).
5. Sasaki, Y.T., Ideue, T., Sano, M., Mituyama, T. & Hirose, T. MENepsilon/beta noncoding RNAs are essential for structural integrity of nuclear paraspeckles. *Proc Natl Acad Sci U S A* **106**, 2525-30 (2009).
6. Clemson, C.M. et al. An architectural role for a nuclear noncoding RNA: NEAT1 RNA is essential for the structure of paraspeckles. *Mol Cell* **33**, 717-26 (2009).
7. Xing, Y. et al. Dynamic Alternative Splicing During Mouse Preimplantation Embryo Development. *Front Bioeng Biotechnol* **8**, 35 (2020).
8. Huo, L.R. et al. Identification of differentially expressed transcripts and translatants targeted by knock-down of endogenous PCBP1. *Biochim Biophys Acta* **1804**, 1954-64 (2010).
9. Shi, H. et al. PCBP1 depletion promotes tumorigenesis through attenuation of p27(Kip1) mRNA stability and translation. *J Exp Clin Cancer Res* **37**, 187 (2018).

10. Fackelmayer, F.O., Dahm, K., Renz, A., Ramsperger, U. & Richter, A. Nucleic-acid-binding properties of hnRNP-U/SAF-A, a nuclear-matrix protein which binds DNA and RNA in vivo and in vitro. *Eur J Biochem* **221**, 749-57 (1994).
11. Hasegawa, Y. et al. The matrix protein hnRNP U is required for chromosomal localization of Xist RNA. *Dev Cell* **19**, 469-76 (2010).
12. Helbig, R. & Fackelmayer, F.O. Scaffold attachment factor A (SAF-A) is concentrated in inactive X chromosome territories through its RGG domain. *Chromosoma* **112**, 173-82 (2003).
13. Pullirsch, D. et al. The Trithorax group protein Ash2l and Saf-A are recruited to the inactive X chromosome at the onset of stable X inactivation. *Development* **137**, 935-43 (2010).
14. Karousis, E.D. & Muhlemann, O. Nonsense-Mediated mRNA Decay Begins Where Translation Ends. *Cold Spring Harb Perspect Biol* **11**(2019).

Supplementary Fig. 1

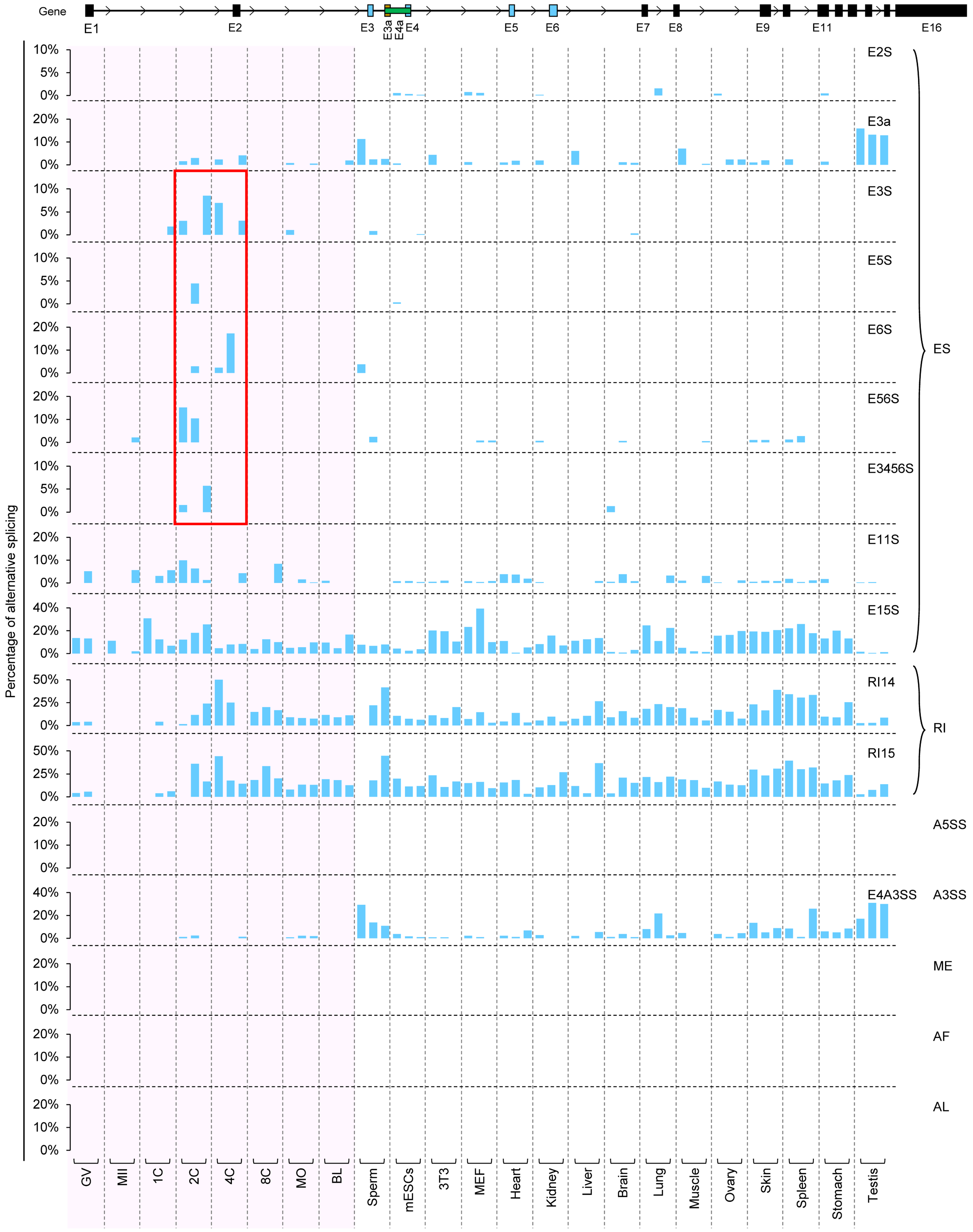

Supplementary Fig. 1 | Percentage of alternative splicing of *Carm1* pre-mRNAs in mouse gametes, early embryos, cell lines, and tissues.

Top panel, illustration of exons of *Carm1* pre-mRNA. Bottom panel, the bar plots depicting the percentage of alternative splicing of *Carm1* pre-mRNAs in mouse gametes, early embryos, cell lines, and tissues. ES, exon-skipping; E2S, exon 2 skipping; E3a, exon 3a inclusive; E3S, exon 3 skipping; E5S, exon 5 skipping; E6S, exon 6 skipping; E56S, exon 5 and 6 skipping; E3456S, exon 3, 4, 5, and 6 skipping; E11S, exon 11 skipping; E15S, exon 15 skipping; RI, retained intron; RI14, retained intron 14; RI15, retained intron 15; A3SS, alternative 3′-splicing site; E4A3SS, alternative 3′-splicing site of exon 4; A5SS, alternative 5′-splicing site; ME, mutually exclusive exons; AF, alternative first exons; AL, alternative last exons. Samples of early embryos are marked by light-red background. Exon 3 to 6 skipping splicing that are with specifically high percentage in two- and four-cell embryos are marked by red rectangle.

Supplementary Fig. 2

a

b

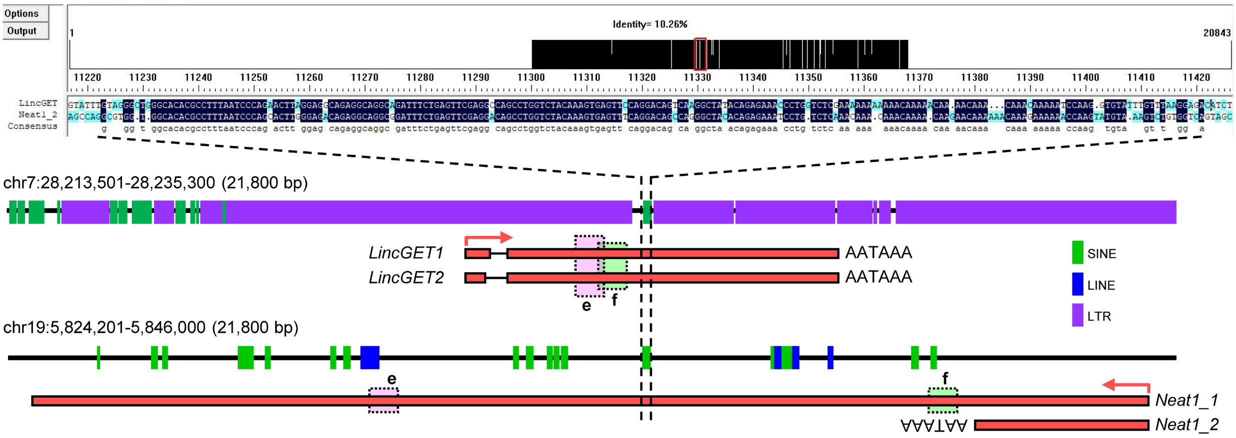

c

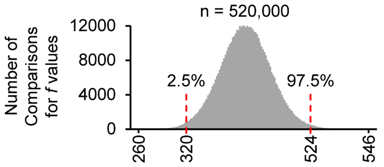

d

	LincGET (1201-1700)	*LincGET* (1601-2100)	*LincGET* (2001-2500)	*LincGET* (2401-2900)
Neat1_2 (3601-4100)	394	370	► 300	372
Neat1_2 (13601-14100)	378	362	378	460
Neat1_2 (14001-14500)	450	► 278	426	426

e

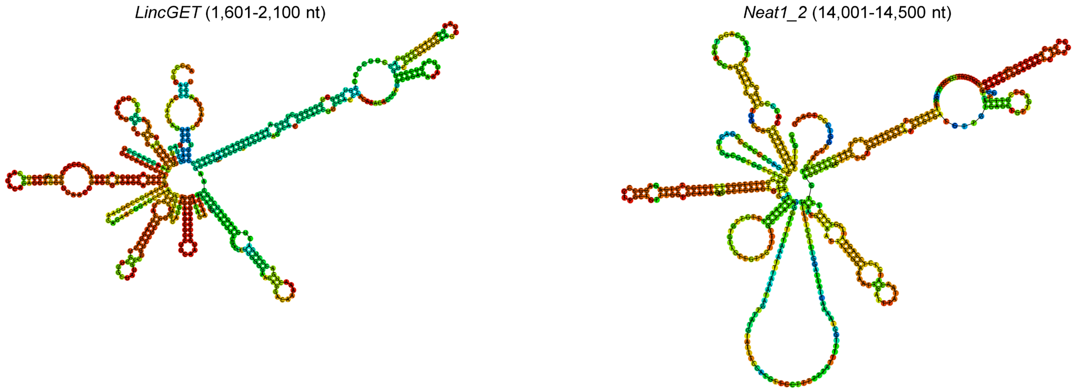

f

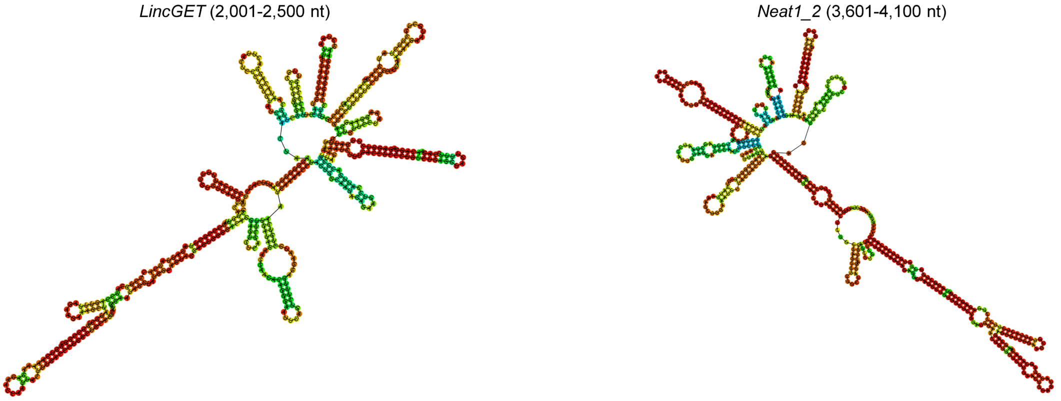

Supplementary Fig. 2 | *LincGET* contains similar structures as *Neat1*.

a. Alignment of *LincGET* and *Neat1* by DNAMAN software. The similarity in primary structure is shown.

b. Illustration of the structure similarity between *LincGET* and *Neat1*. The SINE, LINE, and ERV elements in the gene locus of *LincGET* and *Neat1* are shown. The region with similarity in primary structure is indicated by black dotted lines, and the regions with secondary structure similarity are indicated by light red rectangle with a dashed black border (**e**) and light green rectangle with a dashed black border (**f**).

c. The distribution of *f* values from the comparison between 52 *Neat1* fragments and 10,000 random mRNA fragments (500 nt for each). The confidence interval for difference is 95%, the lower-bound (2.5%) is 320, and the upper-bound (97.5%) is 524.

d. Partial of the comparison result of the secondary structures of *LincGET* and *Neat1* by the *ViennaRNA* Package. Two comparisons with *f* values less than 320 are marked by red triangles. The full comparison results are in Supplementary Table 5.

e, f. Two similar secondary structures of *LincGET* and *Neat1* that are predicted by *ViennaRNA* and visualized by *RNAfold* web serve.

Supplementary Fig. 3

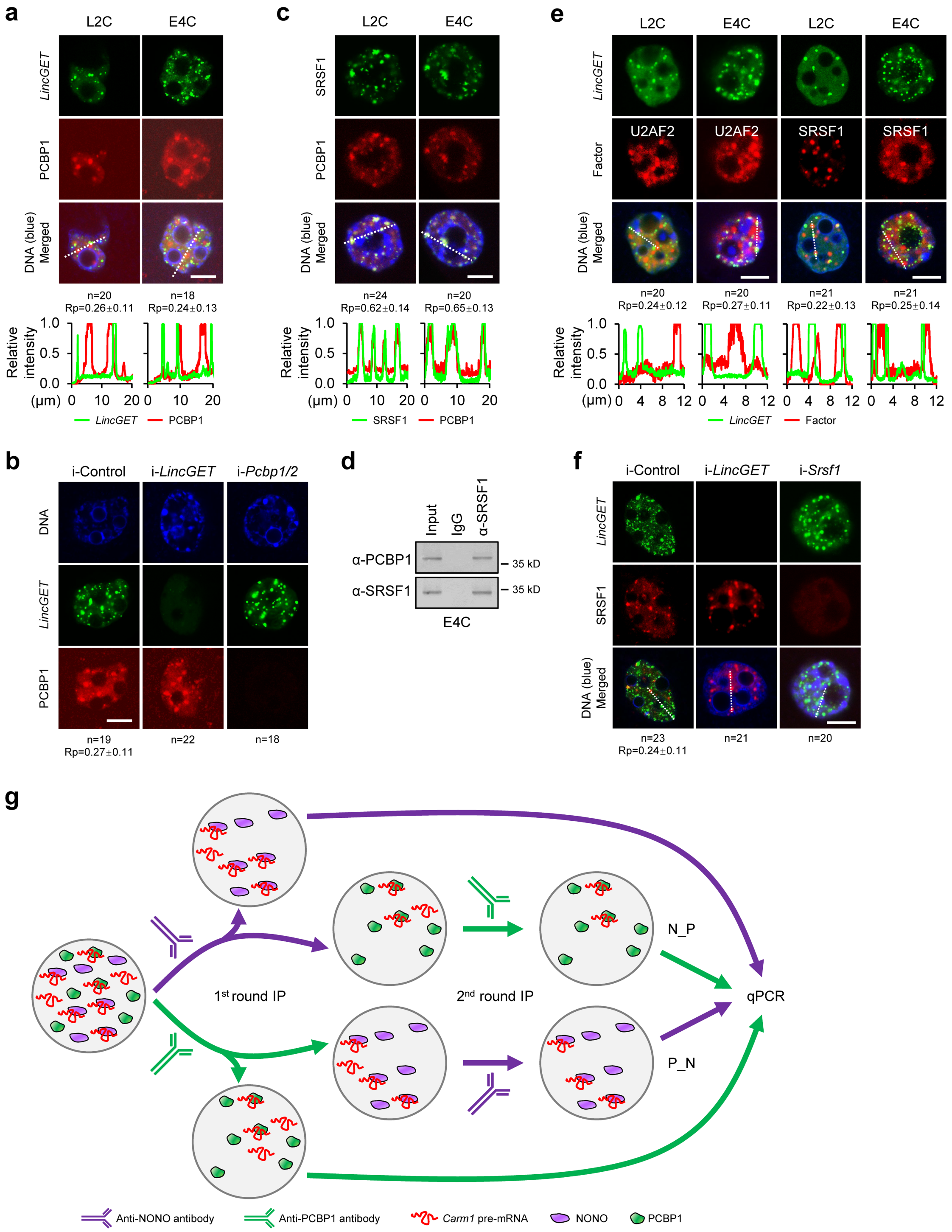

Supplementary Fig. 3 | PCBP1 but not *LincGET* participates in splicing-speckles.

a. Immunostaining results showing that *LincGET* does not co-localize with PCBP1 both in the L2C and E4C embryos. Scale bar, 10 μm. The Pearson's correlation coefficient (Rp) values for co-localization of *LincGET* and PCBP1 are calculated by Fiji/ImageJ. Bottom panel, line scans of the relative fluorescence intensity of signals indicated by the dotted line in left panel.

b. Single channel of fluorescence images in Fig. 4d showing that *LincGET* depletion does not affect the formation of PCBP1-speckles and vice versa. Scale bar, 10 μm.

c. Fluorescence images of representative nuclei reflecting that PCBP1 co-localize with SRSF1 in mouse L2C and E4C embryos. Scale bar, 10 μm. The Rp values for co-localization of PCBP1 and SRSF1 are calculated by Fiji/ImageJ. Bottom panel, line scans of the relative fluorescence intensity of signals indicated by the dotted lines in top panel.

d. co-IP followed by western blot showing PCBP1 forms a complex with SRSF1 in mouse E4C embryos. Three biological replicates were performed. α-, anti; α-IP, the antibody used for IP. kD, kilodalton.

e. Immunostaining results showing that *LincGET* doesn't co-localize with U2AF2 or SRSF1 in both L2C and E4C embryos. Scale bar, 10 μm. The Pearson's R values (Rp) for co-localization of *LincGET* and U2AF2 or SRSF1 are calculated by Fiji/ImageJ. Bottom panel, line scans of the relative fluorescence intensity of signals indicated by the dotted lines in top panel.

f. Immunostaining results showing that *LincGET* depletion doesn't affect the formation of SRSF1-speckles and vice versa. Scale bar, 10 μm.

g. Illustration of sequential co-IP by anti-NONO antibody and then anti-PCBP1 antibody (N_P), as well as by anti-PCBP1 antibody and anti-NONO antibody (P_N).

Supplementary Fig. 4

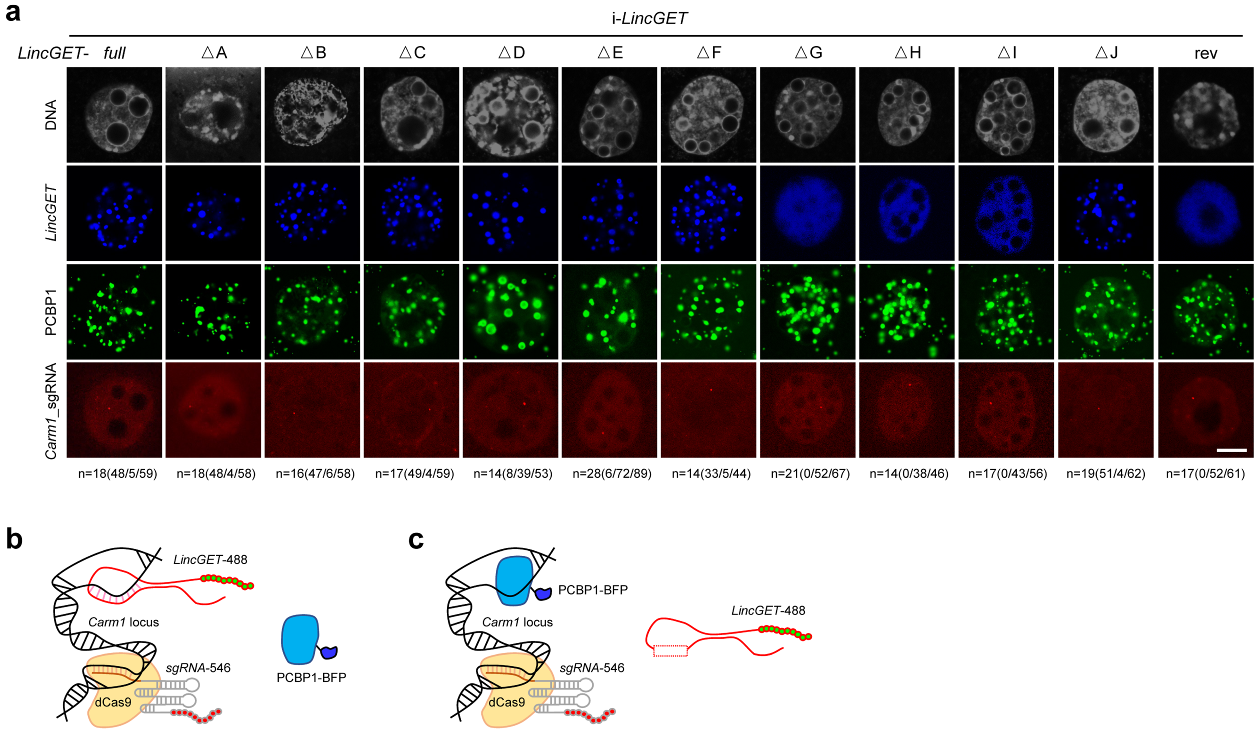

Supplementary Fig. 4 | PCBP1 binds to *Carm1* gene locus when paraspeckles were destroyed or the localization of paraspeckles was disturbed.

a. Single channel of fluorescence images in Fig. 4f showing that PCBP1-speckles cover *Carm1* locus when paraspeckles were destroyed (ΔG, ΔH, or ΔI) or the localization of paraspeckles was disturbed (ΔD or ΔE). The *LincGET* signals were detected from the large amounts of injected *LincGET* or mutants. To make PCBP1-speckles more visible, pseudo blue and green colours are used for *LincGET*-488 and PCBP1-BFP shown in **b** and **c** panels, respectively. Scale bar, 10 μm. The n values for the localization ratio of *LincGET*-speckles and PCBP1 to the *Carm1* gene locus are shown. For full as an example, n=18(48/5/59) means that 18 embryos are analyzed, 59 *Carm1* gene loci are detected, and 48 and 5 out of them are covered by *LincGET*-speckles and PCBP1, respectively.

b, c. Model for localization of Alexa Fluor-488 end-labelled *LincGET*, Alexa Fluor-546 end-labelled sgRNAs-dCas9 complex, and blue fluorescent protein (BFP) fused PCBP1.

Supplementary Fig. 5

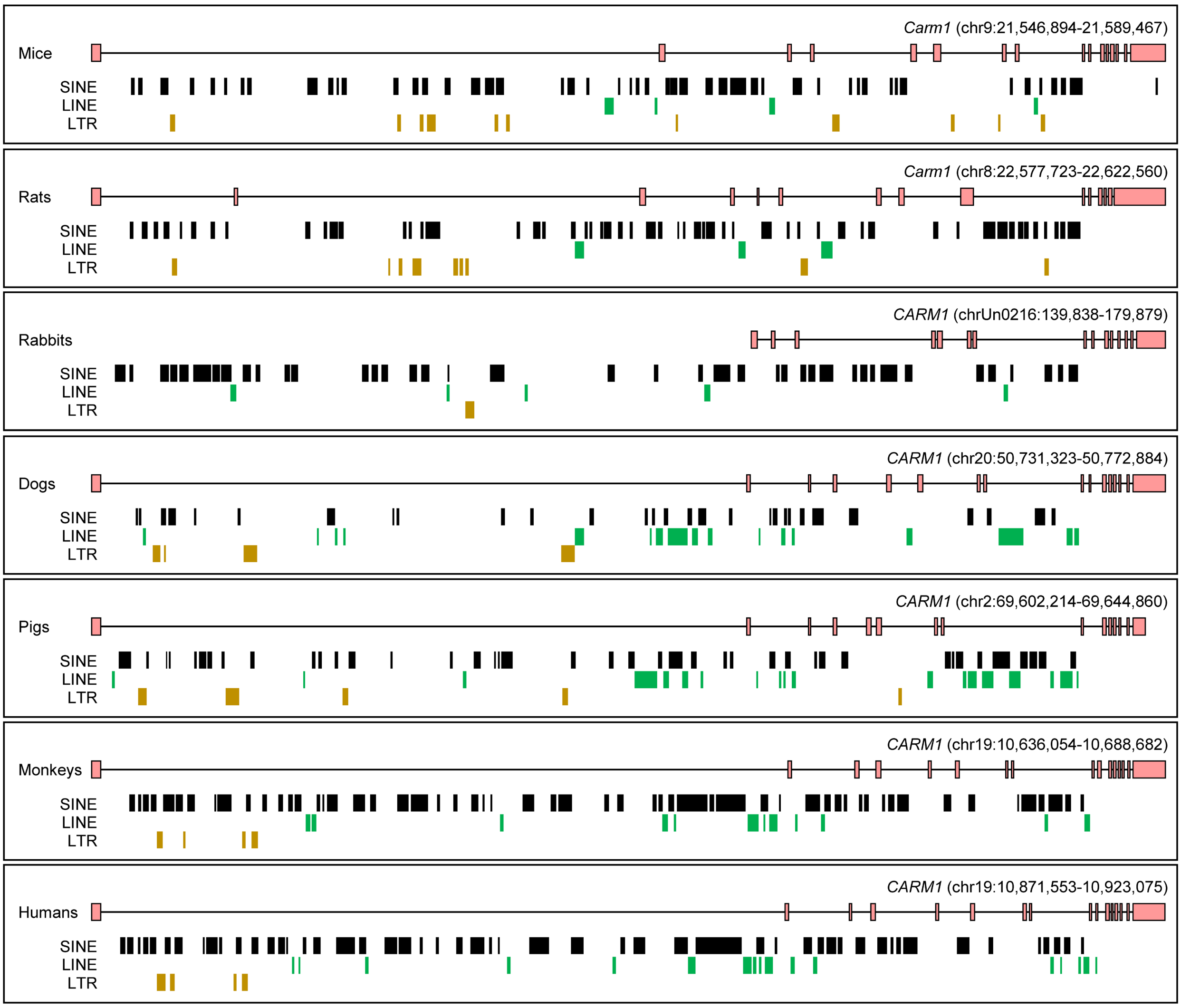

Supplementary Fig. 5 | Comparation of the SINE, LINE, and LTR in the *Carm1* gene locus among mice, rats, rabbits, dogs, pigs, monkeys, and humans.
