# Supplementary material for: Alternative splicing of CARM1 regulated by LincGET-guided paraspeckles biases the first cell fate in mammalian early embryos
Source: Nat Struct Mol Biol. 2024 Apr 24;31(9):1341–54. doi: 10.1038/s41594-024-01292-9 (PMC11402786; doi:10.1038/s41594-024-01292-9)
Supplement: Supplementary file 2 — Reporting Summary [file 41594_2024_1292_MOESM2_ESM.pdf]

nature portfolio

Corresponding author(s): Jiaqiang Wang

Last updated by author(s): Mar 7, 2024

Reporting Summary

Nature Portfolio wishes to improve the reproducibility of the work that we publish. This form provides structure for consistency and transparency in reporting. For further information on Nature Portfolio policies, see our Editorial Policies and the Editorial Policy Checklist.

Statistics

For all statistical analyses, confirm that the following items are present in the figure legend, table legend, main text, or Methods section.

n/a	Confirmed	
☐	☒	The exact sample size (*n*) for each experimental group/condition, given as a discrete number and unit of measurement
☒	☐	A statement on whether measurements were taken from distinct samples or whether the same sample was measured repeatedly
☐	☒	The statistical test(s) used AND whether they are one- or two-sided<br>*Only common tests should be described solely by name; describe more complex techniques in the Methods section.*
☒	☐	A description of all covariates tested
☒	☐	A description of any assumptions or corrections, such as tests of normality and adjustment for multiple comparisons
☐	☒	A full description of the statistical parameters including central tendency (e.g. means) or other basic estimates (e.g. regression coefficient) AND variation (e.g. standard deviation) or associated estimates of uncertainty (e.g. confidence intervals)
☐	☒	For null hypothesis testing, the test statistic (e.g. *F*, *t*, *r*) with confidence intervals, effect sizes, degrees of freedom and *P* value noted<br>*Give P values as exact values whenever suitable.*
☒	☐	For Bayesian analysis, information on the choice of priors and Markov chain Monte Carlo settings
☒	☐	For hierarchical and complex designs, identification of the appropriate level for tests and full reporting of outcomes
☐	☒	Estimates of effect sizes (e.g. Cohen's *d*, Pearson's *r*), indicating how they were calculated

Our web collection on statistics for biologists contains articles on many of the points above.

Software and code

Policy information about availability of computer code

Data collection	The majority of SPAR-seq data were mainly acquired from an Illumina MiSeq sequencer with 300 bp paired-end sequencing reactions (PE300) at BGI company (https://www.genomics.cn/), while partial were acquired from a PacBio Sequel System according to the standard PacBio Iso-Seq procedures at Annoroad company (http://www.annoroad.com/) mixed with samples from other experiments.<br>The SHAPE-MaP data and the ChIP-seq data were both acquired from an Illumina HiSeq 2500 with 150 bp paired-end sequencing reactions (PE150) at BGI company (https://www.genomics.cn/). For exon-skipping splicing percentage analysis from published data, the raw data were downloaded from the GEO database.
Data analysis	For analysis of SPAR-seq data from PE300, clean reads were merged by pear (v0.9.6), reads were counted by bowtie2 (v2.2.5) and transferred into bam files with samtools (v1.9), and the Carm1_E3a_analysis.py (https://github.com/NEAU-Wang-lab/SPAR-seq) was used to note reads.<br>For analysis of SPAR-seq data from PacBio, CCS reads were generated using ccs (v5.0.0) and converted to .fastq format using bam2fastq in the pbbam (v1.0.6). The number of passes for each of the raw CCS reads was generated using GetCCSpass.pl (https://github.com/Lulab-IGDB/polyA_analysis/blob/main/bin/). The Carm1_SPAR_reads_extract.py, Carm1_AS_analysis.py, and Carm1_E3a_analysis.py (https://github.com/NEAU-Wang-lab/SPAR-seq) were used to extract and count AS reads.<br>For analysis of SHAPE-MaP data, ShapeMapper2 (v2.1.5) was used. The reads were mapped to target sequences by bowtie2 (v2.2.5). RNA secondary structures were modeled by Superfold (v1.0). The RNA stem-loop structures for specific fragments were produced by VARNA (v3.93). The .ct files from Superfold were used for visualization and varna_colors.txt files from ShapeMapper2 output were used for the reactivity coloring.<br>For analysis of ChIP-seq data, reads were trimmed by trim_galore (v0.6.7), mapped to mouse genome sequences (https://ftp.ebi.ac.uk/pub/databases/gencode/Gencode_mouse/release_M29/GRCm39.primary_assembly.genome.fa.gz) by bwa (v 0.7.17-r1188), and transferred into .bam files with samtools (v1.9). The .bam files were loaded into the IGV (2.16.2) for visualization. Peak calling was performed by macs2 (2.1.1.20160309) software, after duplex removing by picard (2.18.29-0) software.

For exon-skipping splicing percentage analysis from published data, the trim_galore (v0.6.7) software was used to trim the original data with the default parameters. Next, the STAR software was used to align reads to mouse genome sequences (https://ftp.ebi.ac.uk/pub/databases/gencode/Gencode_mouse/release_M29/GRCm39.primary_assembly.genome.fa.gz) with default parameters. Then, the RMATS (v4.1.0) software was used to analyze the alternative splicing events and Rmats2sashimiplot (v2.0.4) software was used for visualization.

For manuscripts utilizing custom algorithms or software that are central to the research but not yet described in published literature, software must be made available to editors and reviewers. We strongly encourage code deposition in a community repository (e.g. GitHub). See the Nature Portfolio guidelines for submitting code & software for further information.

Data

Policy information about availability of data

All manuscripts must include a data availability statement. This statement should provide the following information, where applicable:

- Accession codes, unique identifiers, or web links for publicly available datasets
- A description of any restrictions on data availability
- For clinical datasets or third party data, please ensure that the statement adheres to our policy

Data availability
The sequencing data of SPAR-seq, SHAPE-MaP-seq, and LincGET-ChIP-seq have been deposited in the Genome Sequence Archive of Beijing Institute of Genomics, Chinese Academy of Sciences (GSA, http://gsa.big.ac.cn/) with the accession number of CRA007472, CRA007494, and CRA007495, respectively. This study also includes analysis of the following published data in Gene Expression Omnibus database (GEO): GSE85019, GSE71257, GSE163724, GSE135457, GSE45719, GSE127106, GSE138760, GSE153530, GSE98150, GSE66582, GSE135678, GSE151704, GSE171760, GSE160894, GSE169632, GSE137630, GSE161998, GSE180259, GSE165133, GSE117815, GSE178298, GSE162352, GSE181800, GSE173471, GSE242289, GSE194115, GSE194203, GSE201938, GSE226534, GSE192404, GSE151260, GSE156568, GSE228894, GSE199546, GSE189015, GSE150510, GSE148019, GSE184348, GSE179888, GSE181651, GSE197122, GSE167360, GSE234841, GSE202260, GSE190199, GSE114450, GSE196236, GSE147574, GSE235546, GSE71434, GSE159484, and GSE149785, and data in GSA: CRA007513.
The mouse genome sequences are available at the website https://ftp.ebi.ac.uk/pub/databases/gencode/Gencode_mouse/release_M29/GRCm39.primary_assembly.genome.fa.gz.

Research involving human participants, their data, or biological material

Policy information about studies with human participants or human data. See also policy information about sex, gender (identity/presentation), and sexual orientation and race, ethnicity and racism.

Reporting on sex and gender	n/a
Reporting on race, ethnicity, or other socially relevant groupings	n/a
Population characteristics	n/a
Recruitment	n/a
Ethics oversight	n/a

Note that full information on the approval of the study protocol must also be provided in the manuscript.

Field-specific reporting

Please select the one below that is the best fit for your research. If you are not sure, read the appropriate sections before making your selection.

☒ Life sciences ☐ Behavioural & social sciences ☐ Ecological, evolutionary & environmental sciences

For a reference copy of the document with all sections, see nature.com/documents/nr-reporting-summary-flat.pdf

Life sciences study design

All studies must disclose on these points even when the disclosure is negative.

Sample size	No statistical methods were used to pre-determine sample sizes but our sample sizes are similar to those used in previous publications (DOI: 10.1016/j.cell.2018.11.027).
Data exclusions	No data was excluded from the analysis.
Replication	All experiments were reliably reproduced. Each experiment was performed independently at least three times, but usually many more times.
Randomization	The embryos or cells were randomly assigned to each experimental groups.
Blinding	Blinding was not performed due to the unambiguous nature of measurements and systematic analyses used in these experiments.

Reporting for specific materials, systems and methods

We require information from authors about some types of materials, experimental systems and methods used in many studies. Here, indicate whether each material, system or method listed is relevant to your study. If you are not sure if a list item applies to your research, read the appropriate section before selecting a response.

Materials & experimental systems

n/a	Involved in the study
☐	☒ Antibodies
☐	☒ Eukaryotic cell lines
☒	☐ Palaeontology and archaeology
☐	☒ Animals and other organisms
☒	☐ Clinical data
☒	☐ Dual use research of concern
☒	☐ Plants

Methods

n/a	Involved in the study
☐	☒ ChIP-seq
☒	☐ Flow cytometry
☒	☐ MRI-based neuroimaging

Antibodies

Antibodies used

mouse monoclonal [G-2] anti-CARM1 (Santa Cruz, sc-393381),
chicken polyclonal anti-GFP (Abcam, ab13970),
rat monoclonal [YL1/2] anti-Tubulin (Abcam, ab6160),
goat polyclonal anti-PCBP1 (Abcam, ab109577),
rat monoclonal [EPR14859(2)] anti-PCBP2 (Abcam, ab200835),
rat monoclonal [EPR8239] anti-SRSF1 (Abcam, ab129108),
rabbit polyclonal anti-HA (Abcam, ab9110),
rabbit polyclonal anti-NONO (Abcam, ab70335),
rabbit polyclonal anti-PSPC1 (Abcam, ab104238),
rabbit polyclonal anti-HNRNPU (Abcam, ab20666),
rabbit polyclonal anti-U2AF2 (Abcam, ab37530),
rabbit polyclonal anti-H3R26me2 (Abcam, ab127095)
FITC-conjugated donkey anti-Chicken IgY polyclonal secondary antibody (Invitrogen, SA1-72000),
Alexa Fluor Plus 555-conjugated goat anti-Mouse IgG polyclonal secondary antibody (Invitrogen, A32727),
Alexa Fluor Plus 555-conjugated donkey anti-Goat IgG polyclonal secondary antibody (Invitrogen, A32816),
Alexa Fluor Plus 555-conjugated donkey anti-Rabbit IgG polyclonal secondary antibody (Invitrogen, A32794),
Alexa Fluor Plus 555-conjugated donkey anti-Rat IgG polyclonal secondary antibody (Invitrogen, A48270),
Alexa Fluor 488-conjugated rabbit anti-Rat IgG polyclonal secondary antibody (Invitrogen, A-21210),
HRP-conjugated goat anti-Rabbit IgG polyclonal secondary antibody (Invitrogen, A27036),
HRP-conjugated mouse anti-Goat IgG polyclonal secondary antibody (Invitrogen, 31400),
HRP-conjugated goat anti-Rat IgG polyclonal secondary antibody (Invitrogen, 31470)

Validation

All antibodies used are commonly used in the field and have been validated in previous publications/by the manufacturer.
References and manufacturer validations can be found here:
mouse monoclonal [G-2] anti-CARM1 (Santa Cruz, sc-393381; RRID: AB_2732840): https://www.scbt.com/p/carm1-antibody-g-2;
Chicken polyclonal anti-GFP (Abcam, ab13970; RRID: AB_300798): https://www.abcam.cn/gfp-antibody-ab13970.html;
Rat monoclonal [YL1/2] anti-Tubulin (Abcam, ab6160; RRID: AB_305328): https://www.abcam.cn/tubulin-antibody-yl12-loading-control-ab6160.html;
Rabbit polyclonal anti-PCBP1 (Abcam, ab74793; RRID: AB_1281060): https://www.abcam.cn/pcbp1-antibody-ab74793.html;
rat monoclonal [EPR14859(2)] anti-PCBP2 (Abcam, ab200835; RRID: AB_3094741): https://www.abcam.cn/products/primary-antibodies/pcbp2hnrnp-e2-antibody-epr148592-ab200835.html;
Rabbit monoclonal anti-SRSF1 (Abcam, ab129108; RRID: AB_11141636): https://www.abcam.cn/sf2-antibody-epr8239-ab129108.html;
Rabbit polyclonal anti-HA (Abcam, ab9110; RRID: AB_307019): https://www.abcam.cn/ha-tag-antibody-chip-grade-ab9110.html;
Rabbit polyclonal anti-NONO (Abcam, ab70335; RRID: AB_1269576): https://www.abcam.cn/nmt55-p54nrb-antibody-ab70335.html;
Rabbit polyclonal anti-PSPC1 (Abcam, ab104238; RRID: AB_11157752): https://www.abcam.cn/pspc1-antibody-ab104238.html;
Rabbit polyclonal anti-hnRNPU (Abcam, ab20666; RRID: AB_732983): https://www.abcam.cn/hnrnp-up120-antibody-ab20666.html;
rabbit polyclonal anti-U2AF2 (Abcam, ab37530; RRID:AB_883336): https://www.abcam.cn/products/primary-antibodies/u2af65-antibody-ab37530.html;
Rabbit polyclonal anti-H3R26me2 (Abcam, ab127095; RRID: AB_2732841): https://www.abcam.cn/histone-h3-symmetric-di-methyl-r26-antibody-ab127095.html.

Eukaryotic cell lines

Policy information about cell lines and Sex and Gender in Research

Cell line source(s)

The mouse epiblast stem cells (mEpiSCs) were established in Qi Zhou's lab in State Key Laboratory of Stem Cell and Reproductive Biology, Institute of Zoology, Chinese Academy of Sciences.
The MCF7 cells were purchased from ATCC (ATCC, HTB-22).

Authentication	Identity of mEpiSCs cell line was frequently checked by the morphological features, but not authenticated.<br>Identity of MCF7 cell line was frequently checked by the morphological features, but not authenticated.
Mycoplasma contamination	The mEpiSCs cell line was regularly tested for mycoplasma contamination and no contamination was found.<br>The MCF7 cell line was regularly tested for mycoplasma contamination and no contamination was found.
Commonly misidentified lines<br>(See ICLAC register)	No commonly misidentified cell lines were used.

Animals and other research organisms

Policy information about studies involving animals; ARRIVE guidelines recommended for reporting animal research, and Sex and Gender in Research

Laboratory animals	The CD1 (ICR) mice were purchased from Vital River company. All mice used for experiments were seven- to eight-week-old. All mice were housed under specific pathogen-free conditions with a 12-hour dark/light cycle, an ambient temperature ranging from 21°C to 26°C, and a humidity level of 50% to 60%, in the animal care facilities at the Institute of Zoology, Chinese Academy of Sciences. To obtain preimplantation embryos, female mice were injected with 10 U of pregnant mare serum gonadotropin (PMSG, Prospec, HOR-272) and 10 U of human chorionic gonadotropin (hCG, Prospec, HOR-250) at 46- to 48-hour intervals, and then crossed with 7- to 8-week-old CD1 (ICR) male mice. Embryos were collected at the following times post hCG injection: early one-cell stage (phCG 19 hours), late one-cell stage (phCG 30 hours), early two-cell stage (phCG 39 hours), late two-cell stage (phCG 48 hours), early four-cell stage (phCG 54 hours), late four-cell stage (phCG 62 hours), early eight-cell stage (phCG 68 hours), late eight-cell stage (phCG 74 hours), 16-cell stage (phCG 80 hours), 32-cell stage (phCG 90 hours), early blastocyst stage (phCG 98 hours), and late blastocyst stage (phCG 114 hours).
Wild animals	No wild animals were used in the study.
Reporting on sex	No sex-based experiments were performed.
Field-collected samples	No field-collected samples were used.
Ethics oversight	All the mouse procedures are carried out in compliance with the guidelines of the Animal Care and Use Committee of the Institute of Zoology, Chinese Academy of Sciences, and the Animal Care and Use Committee of the Northeast Agricultural University.

Note that full information on the approval of the study protocol must also be provided in the manuscript.

Plants

Seed stocks	n/a
Novel plant genotypes	n/a
Authentication	n/a

ChIP-seq

Data deposition

☒ Confirm that both raw and final processed data have been deposited in a public database such as GEO.

☒ Confirm that you have deposited or provided access to graph files (e.g. BED files) for the called peaks.

Data access links<br>*May remain private before publication.*	The raw data have been deposited in GSA with the access link, https://ngdc.cncb.ac.cn/gsa/s/QcU5dfun.<br>The processed data in bam file and the bed file for the call peaks have been deposited in the OMIX, China National Center for Bioinformation / Beijing Institute of Genomics, Chinese Academy of Sciences (https://ngdc.cncb.ac.cn/omix), with accession no. OMIX004027 and OMIX004026, respectively.
Files in database submission	input: input_1.fq.gz, input_2.fq.gz<br>LincGET_ChIP_rep1: V350003627_L04_612_1.fq.gz, V350003627_L04_612_2.fq.gz<br>LincGET_ChIP_rep2: V350003627_L04_613_1.fq.gz, V350003627_L04_613_2.fq.gz<br>LincGET_ChIP_rep3, V350003627_L04_614_1.fq.gz, V350003627_L04_614_2.fq.gz
Genome browser session<br>(e.g. UCSC)	https://ftp.ebi.ac.uk/pub/databases/gencode/Gencode_mouse/release_M30/GRCm39.primary_assembly.genome.fa.gz<br>https://ftp.ebi.ac.uk/pub/databases/gencode/Gencode_mouse/release_M32/<br>gencode.vM32.chr_patch_hapl_scaff.annotation.gtf.gz

Methodology

Replicates	One replicates for input, and three replicates for ChIP.
Sequencing depth	Sequencing depth for input, LincGET_ChIP_rep1, LincGET_ChIP_rep2, and LincGET_ChIP_rep3 were 4,673,619,900, 4,785,825,600,

Sequencing depth	4,790,067,900, and 4,807,644,300, respectively.<br>Total number of reads for input, LincGET_ChIP_rep1, LincGET_ChIP_rep2, and LincGET_ChIP_rep3 were 31,157,466, 31,905,504, 31,933,786, and 32,050,962, respectively.<br>Total number of uniquely mapped reads for input, LincGET_ChIP_rep1, LincGET_ChIP_rep2, and LincGET_ChIP_rep3 were 29,990,741, 29,211,776, 29,118,337, and 29,271,751, respectively.<br>Length of reads is 150 bp. Reads were paired-end.
Antibodies	rabbit polyclonal anti-HA antibody (Abcam, ab9110)

Peak calling parameters

```
# index building
$ bwa index mm10.fa -p mm10
# reads mapping
$ bwa mem -M -t 8 index/mm10 input_1.fq.gz input_2.fq.gz > input.bwa.sam
$ bwa mem -M -t 8 index/mm10 V350003627_L04_612_1.fq.gz V350003627_L04_612_2.fq.gz > LincGET_rep1.bwa.sam
$ bwa mem -M -t 8 index/mm10 V350003627_L04_613_1.fq.gz V350003627_L04_613_2.fq.gz > LincGET_rep2.bwa.sam
$ bwa mem -M -t 8 index/mm10 V350003627_L04_614_1.fq.gz V350003627_L04_614_2.fq.gz > LincGET_rep3.bwa.sam
# unmapped reads removing and sorting
$ samtools view -bS -F 4 input.bwa.sam > input.bwa.mapped.bam
$ samtools view -bS -F 4 LincGET_rep1.bwa.sam > LincGET_rep1.bwa.mapped.bam
$ samtools view -bS -F 4 LincGET_rep2.bwa.sam > LincGET_rep2.bwa.mapped.bam
$ samtools view -bS -F 4 LincGET_rep3.bwa.sam > LincGET_rep3.bwa.mapped.bam
$ samtools sort input.bwa.mapped.bam > input.bwa.mapped.sort.bam
$ samtools sort LincGET_rep1.bwa.mapped.bam > LincGET_rep1.bwa.mapped.sort.bam
$ samtools sort LincGET_rep2.bwa.mapped.bam > LincGET_rep2.bwa.mapped.sort.bam
$ samtools sort LincGET_rep3.bwa.mapped.bam > LincGET_rep3.bwa.mapped.sort.bam
# duplex removing
$ picard MarkDuplicates REMOVE_DUPLICATES=true I= input.bwa.mapped.sort.bam O= input_deduplicate_bam M= input.log; done
$ picard MarkDuplicates REMOVE_DUPLICATES=true I= LincGET_rep1.bwa.mapped.sort.bam O= LincGET_rep1_deduplicate_bam M= input.log; done
$ picard MarkDuplicates REMOVE_DUPLICATES=true I= LincGET_rep2.bwa.mapped.sort.bam O= LincGET_rep2_deduplicate_bam M= input.log; done
$ picard MarkDuplicates REMOVE_DUPLICATES=true I= LincGET_rep3.bwa.mapped.sort.bam O= LincGET_rep3_deduplicate_bam M= input.log; done
# peak calling
$ macs2 callpeak -t LincGET_rep1_deduplicate_bam -c input_deduplicate_bam -f BAMPE -g mm -n LincGET_rep1 -B -q 0.05
$ macs2 callpeak -t LincGET_rep2_deduplicate_bam -c input_deduplicate_bam -f BAMPE -g mm -n LincGET_rep1 -B -q 0.05
$ macs2 callpeak -t LincGET_rep3_deduplicate_bam -c input_deduplicate_bam -f BAMPE -g mm -n LincGET_rep1 -B -q 0.05
```

Data quality	The number of peaks at FDR 5% and above 5-fold enrichment in LincGET_ChIP_rep1, LincGET_ChIP_rep2, and LincGET_ChIP_rep3 are 43,794, 43,095, and 43,082, respectively.
Software	The deep sequencing datasets of LincGET-ChIP-seq were trimmed by trim_galore (v 0.6.7) software with default parameters. The reads were mapped to mouse genome sequences (https://ftp.ebi.ac.uk/pub/databases/gencode/Gencode_mouse/release_M29/GRCm39.primary_assembly.genome.fa.gz) by bwa (v 0.7.17-r1188) software with default parameters and transferred into .bam files with samtools (v1.9) software. The .bam files were loaded into the IGV for visualization. Peak calling was performed by macs2 (2.1.1.20160309) software, after duplex removing by picard (2.18.29-0) software.
