# Supplementary figures and images for: Alternative splicing of CARM1 regulated by LincGET-guided paraspeckles biases the first cell fate in mammalian early embryos
Source: Nat Struct Mol Biol. 2024 Apr 24;31(9):1341–54. doi: 10.1038/s41594-024-01292-9 (PMC11402786; doi:10.1038/s41594-024-01292-9)

Unmodified Gels Supplementary Fig. 3

d

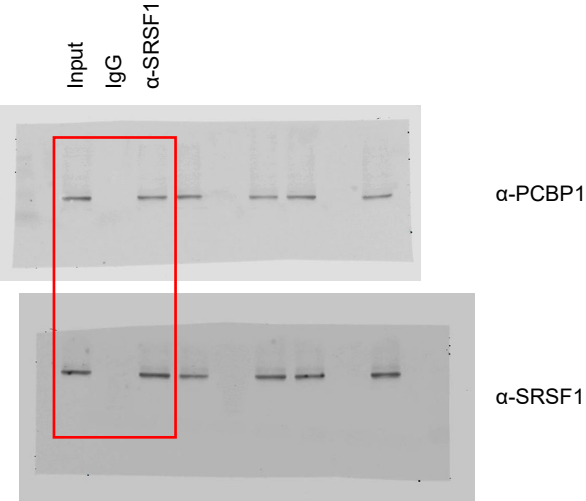

Supplement: Supplementary file 7 — Unprocessed western Blots for Supplementary Fig. 3d. [file 41594_2024_1292_MOESM7_ESM.pdf]

Unmodified Gels Fig. 2

b

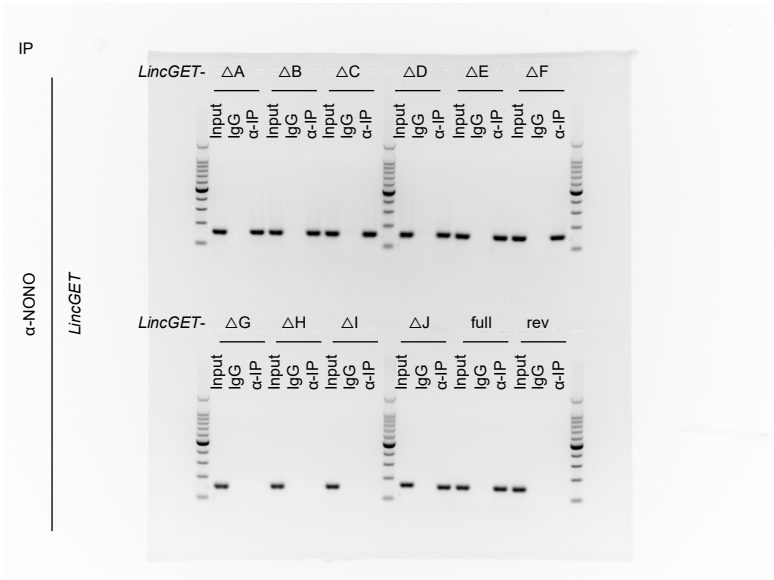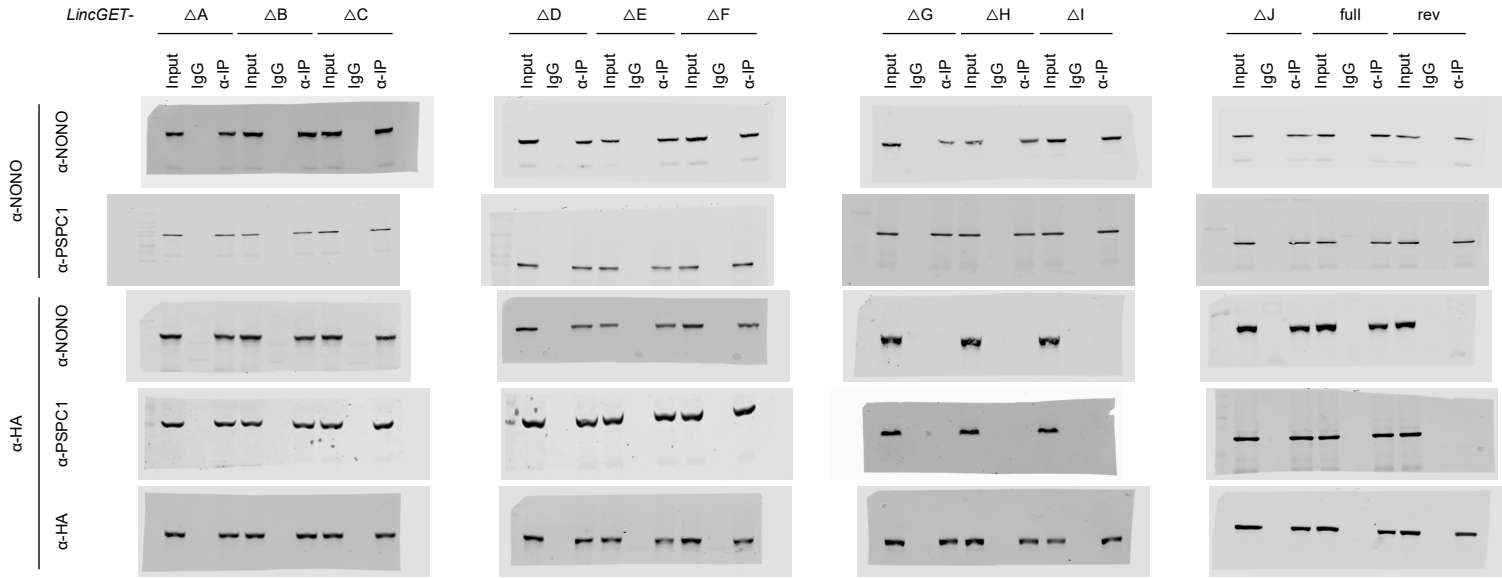

d

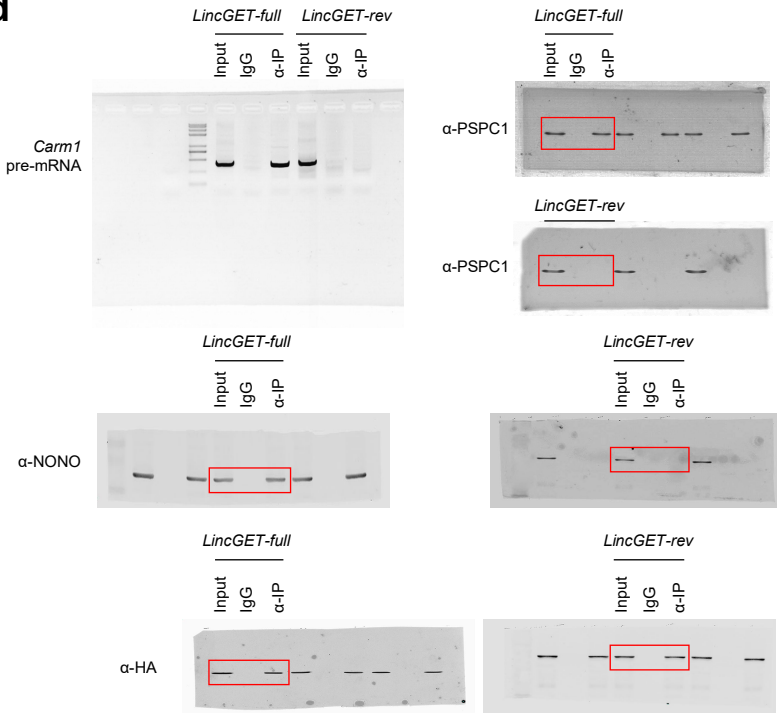

g

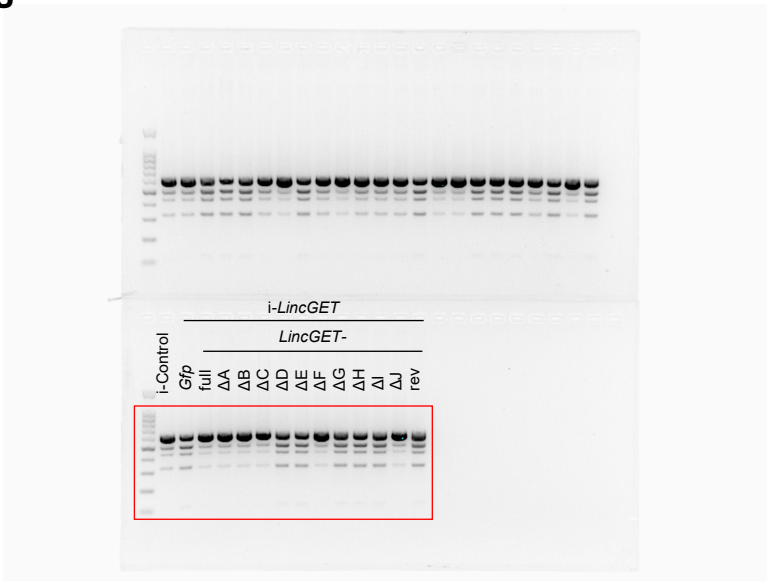

Supplement: Supplementary file 10 — Unprocessed Gels and Western Blots for Fig. 2b,d,g. [file 41594_2024_1292_MOESM10_ESM.pdf]

Unmodified Gels Fig. 3

e

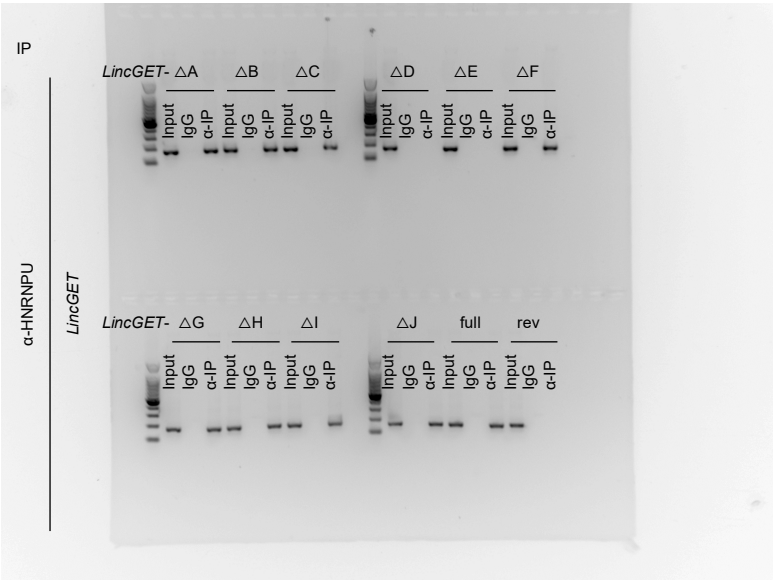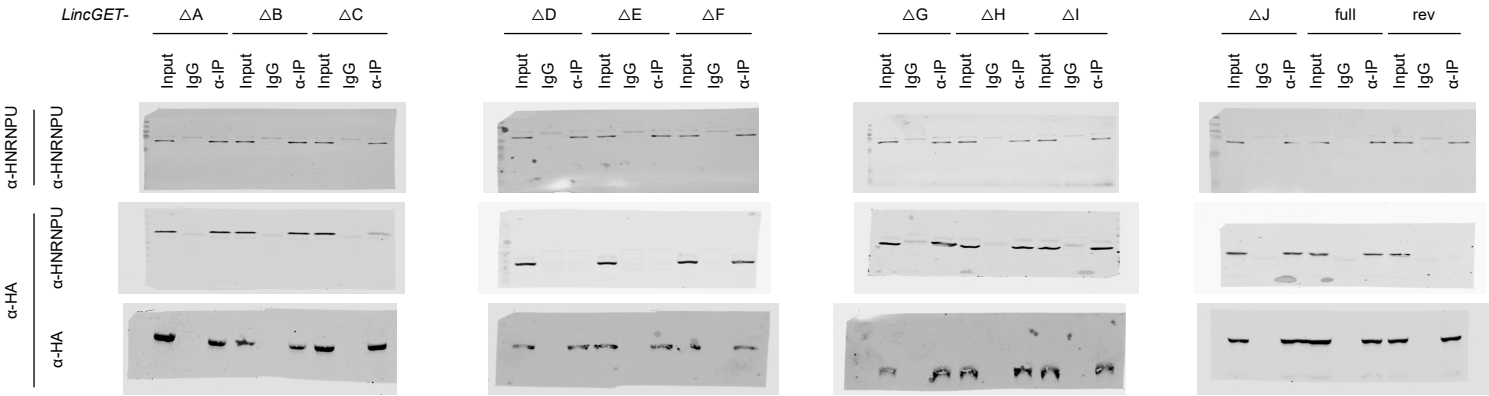

Supplement: Supplementary file 12 — Unprocessed Gels and Western Blots for Fig. 3e. [file 41594_2024_1292_MOESM12_ESM.pdf]

Unmodified Gels Fig. 4

**b**

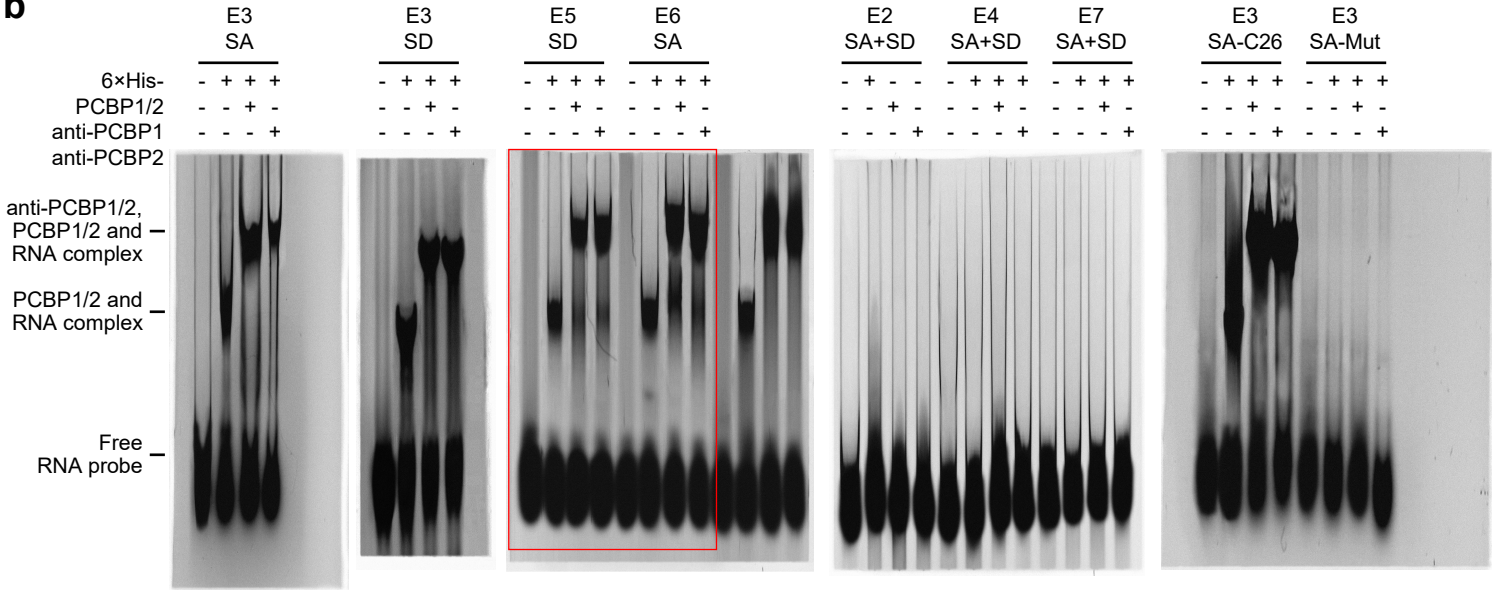

Supplement: Supplementary file 14 — Unprocessed REMSA Blots for Fig. 4b. [file 41594_2024_1292_MOESM14_ESM.pdf]

Unmodified Gels Fig. 5

g

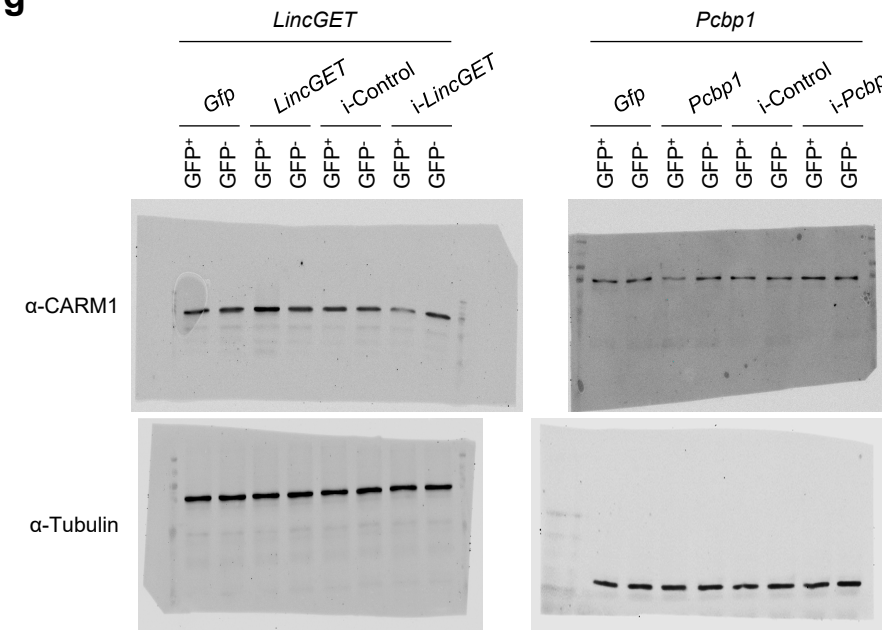

Supplement: Supplementary file 16 — Unprocessed Western Blots for Fig. 5g. [file 41594_2024_1292_MOESM16_ESM.pdf]

Unmodified Gels Extended Data Fig. 3

b

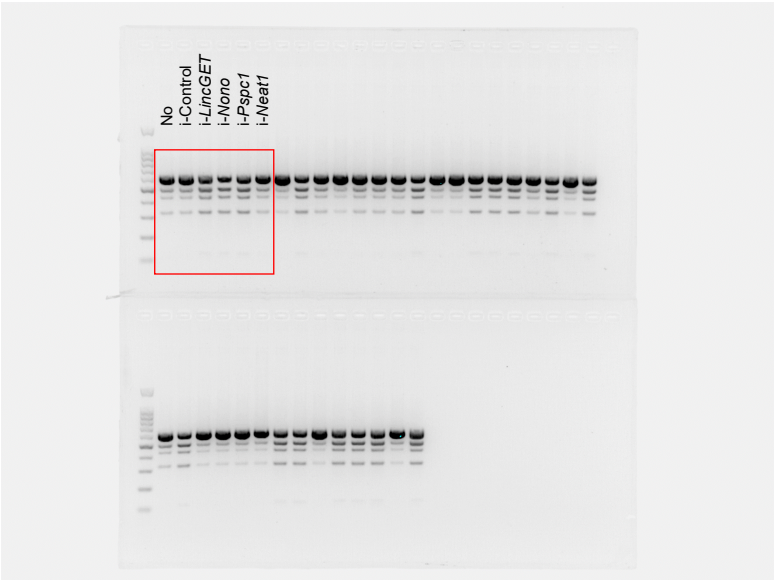

Supplement: Supplementary file 20 — Unprocessed Gels for Extended Data Fig. 3b. [file 41594_2024_1292_MOESM20_ESM.pdf]

**b**

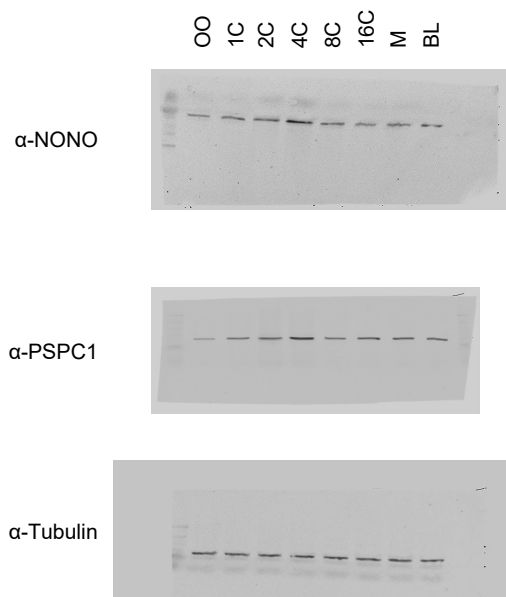

Supplement: Supplementary file 22 — Unprocessed Western Blots for Extended Data Fig. 4b. [file 41594_2024_1292_MOESM22_ESM.pdf]

Unmodified Gels Extended Data Fig. 5

e

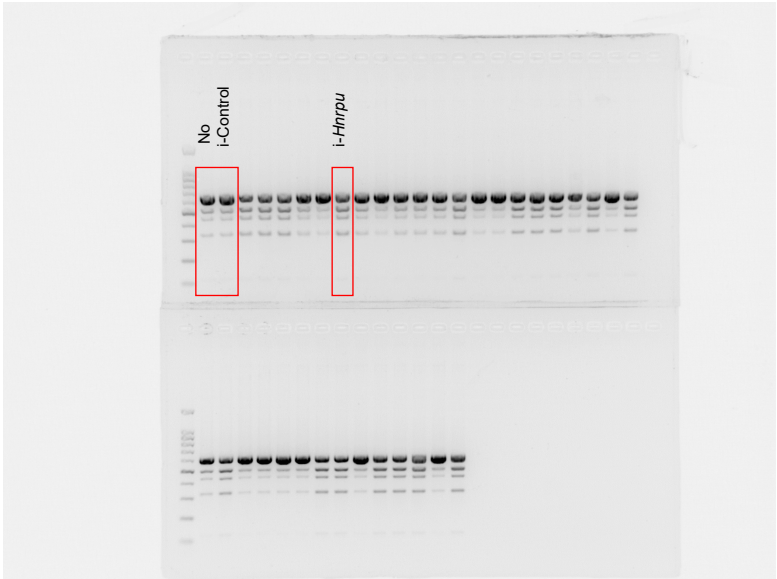

Supplement: Supplementary file 24 — Unprocessed Gels for Extended Data Fig. 5e. [file 41594_2024_1292_MOESM24_ESM.pdf]

Unmodified Gels Extended Data Fig. 7

**b**

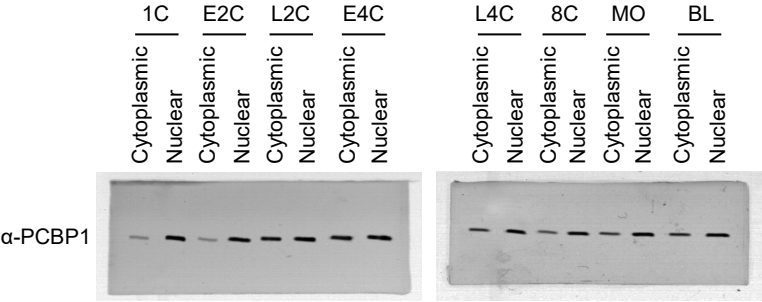

**c**

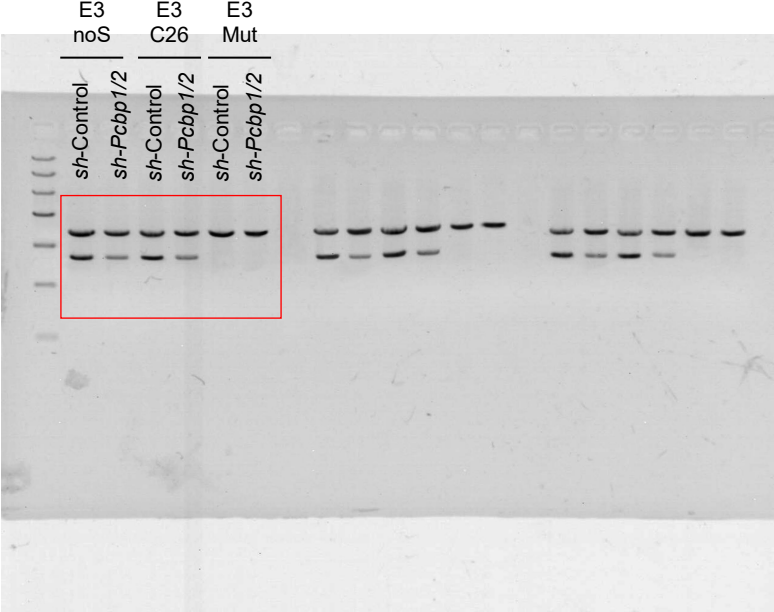

**e**

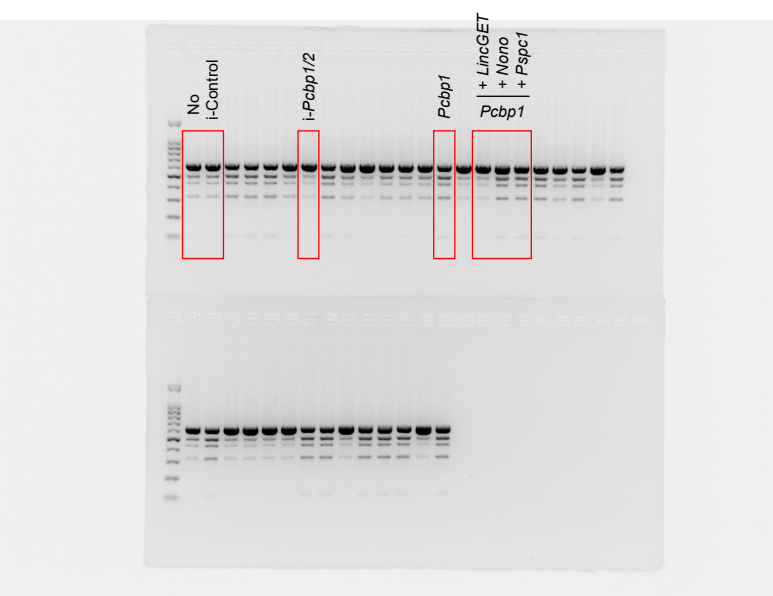

Supplement: Supplementary file 27 — Unprocessed Gels and western Blots for Extended Data Fig. 7b,c,e. [file 41594_2024_1292_MOESM27_ESM.pdf]

Unmodified Gels Extended Data Fig. 9

b

2C

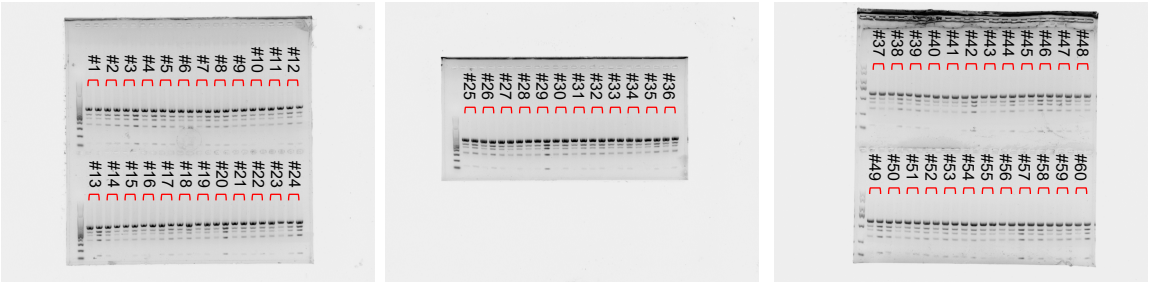

E3S  
E5S  
E6S  
E56S  
E3456S

c

4C

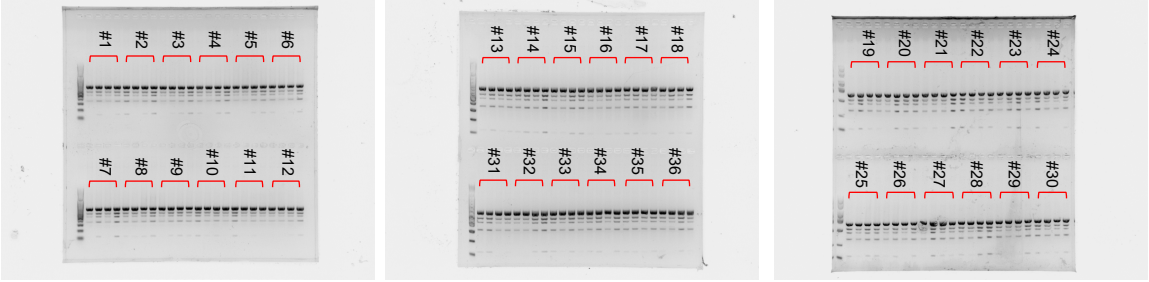

E3S  
E5S  
E6S  
E56S  
E3456S

4C

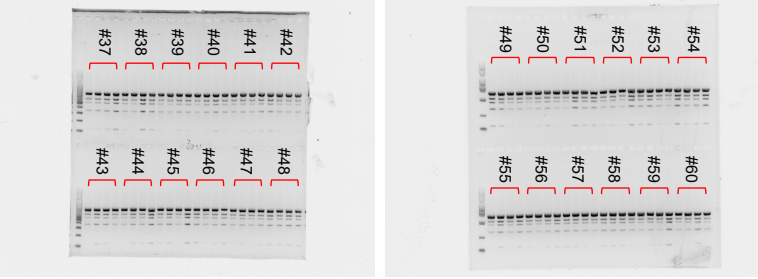

E3S  
E5S  
E6S  
E56S  
E3456S

Supplement: Supplementary file 30 — Unprocessed Gels for Extended Data Fig. 9b,c. [file 41594_2024_1292_MOESM30_ESM.pdf]

Unmodified Gels Extended Data Fig. 10

**b**

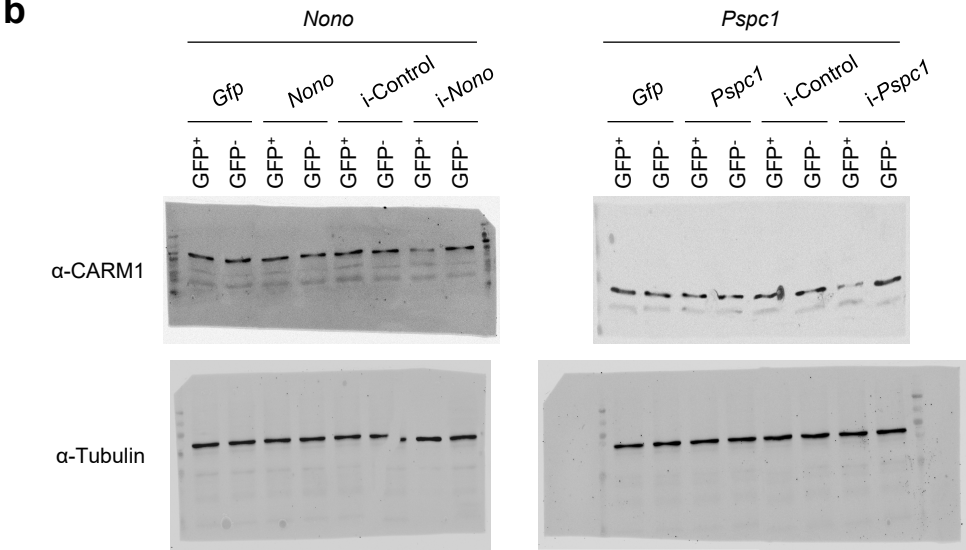

**c**

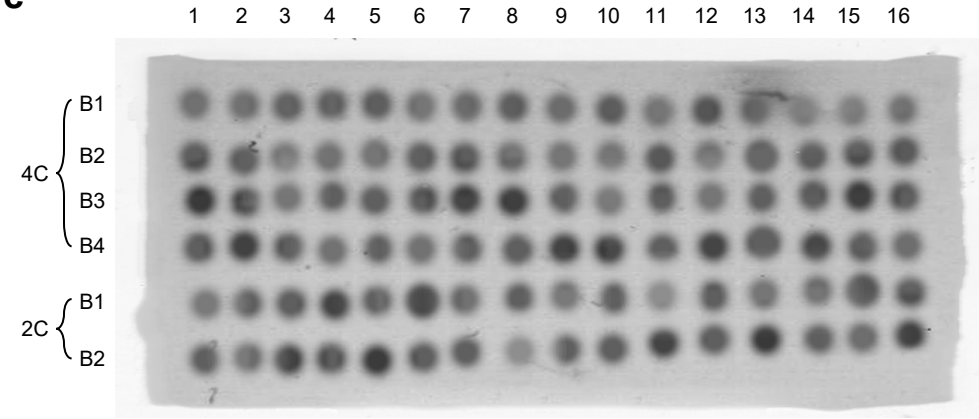

Supplement: Supplementary file 32 — Unprocessed western Blots and dot Blots for Extended Data Fig. 10b,c. [file 41594_2024_1292_MOESM32_ESM.pdf]
