# Supplementary material for: Alternative splicing of CARM1 regulated by LincGET-guided paraspeckles biases the first cell fate in mammalian early embryos
Source: Nat Struct Mol Biol. 2024 Apr 24;31(9):1341–54. doi: 10.1038/s41594-024-01292-9 (PMC11402786; doi:10.1038/s41594-024-01292-9)

Unmodified Gels Extended Data Fig. 2

b

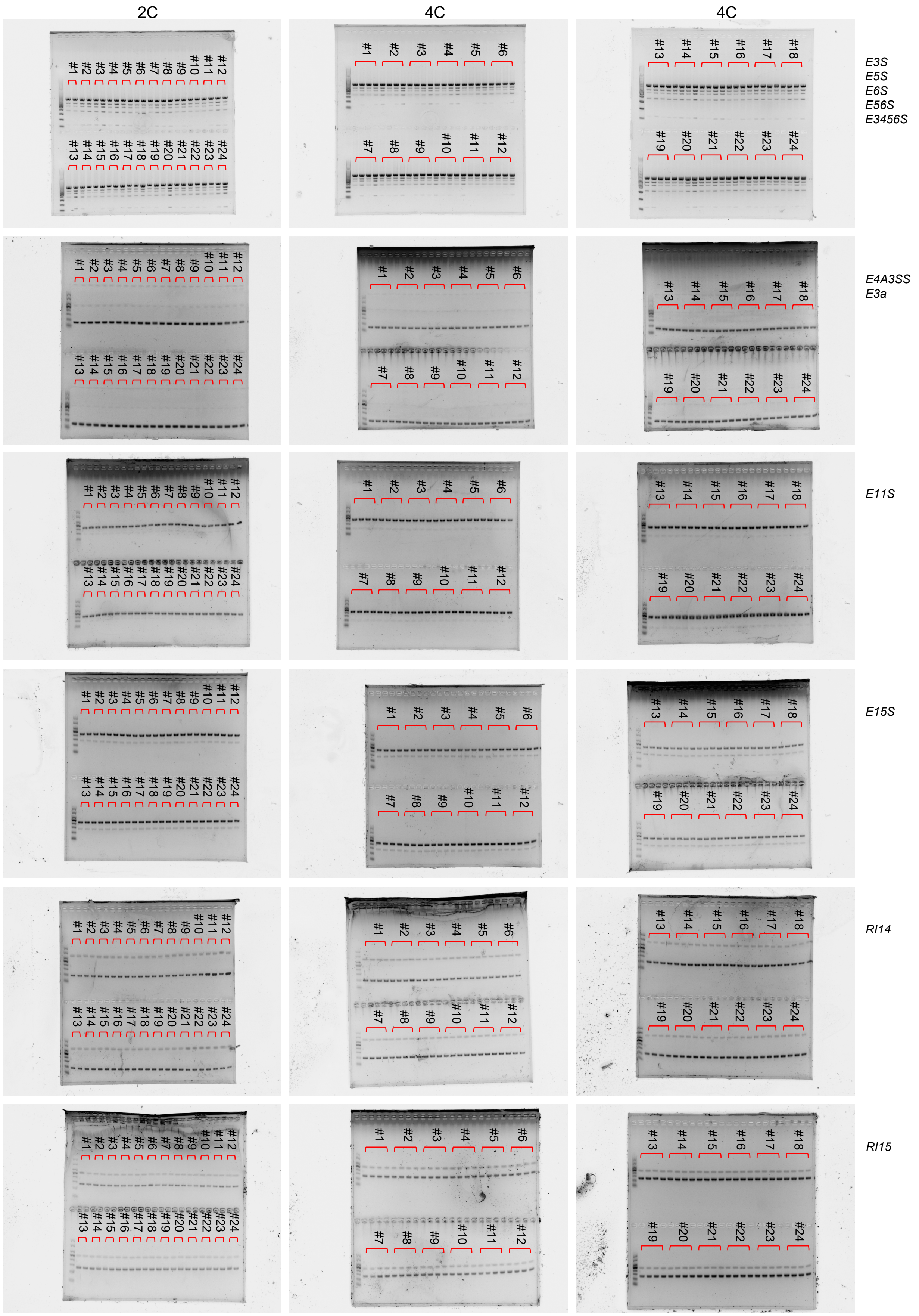

e

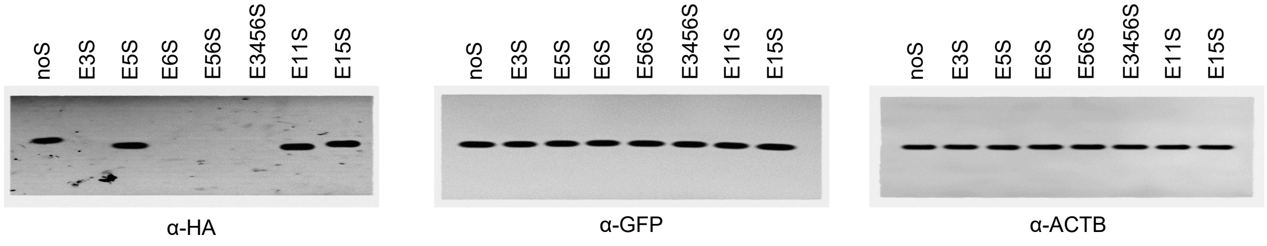

Supplement: Supplementary file 18 — Unprocessed Gels and Western Blots for Extended Data Fig. 2b,e. [file 41594_2024_1292_MOESM18_ESM.pdf]
